# Electrochemical Phosphorochalcogenations for the Synthesis of Phosphorochalcogenothioates

**DOI:** 10.1021/acs.joc.5c03080

**Published:** 2026-03-25

**Authors:** Indrajit Karmakar, Xiang-Wei Huang, Yu-Hao Chen, Chieh-An Cheng, Pei-Chi Kuo, Chin-Fa Lee

**Affiliations:** a Department of Chemistry, 34916 National Chung Hsing University, Taichung City 40227, Taiwan (R.O.C.); b i-Center for Advanced Science and Technology (iCAST), 34916 National Chung Hsing University, Taichung City 40227, Taiwan (R.O.C.); c Innovation and Development Center of Sustainable Agriculture (IDCSA), 34916 National Chung Hsing University, Taichung City 40227, Taiwan (R.O.C.)

## Abstract

We report a robust and environmentally conscious electrosynthetic protocol for the efficient construction of structurally diverse and biologically relevant phosphorochalcogenated compounds. This transformation proceeds through the electrochemical cross-coupling of phosphonothioates with the corresponding thiols or diaryl diselenides using inexpensive NaI or *^n^* Bu_4_NI as both a green electrolyte and redox mediator. The reactions are carried out in CH_3_CN at room temperature under a constant current of 10 mA with Pt/Pt electrodes. This newly developed method offers several notable advantages, including mild and energy-efficient conditions, transition-metal-free operation, short reaction times, moderate to high yields, broad substrate scope, large-scale applicability, operational simplicity, and overall environmental sustainability.

## Graphical abstract

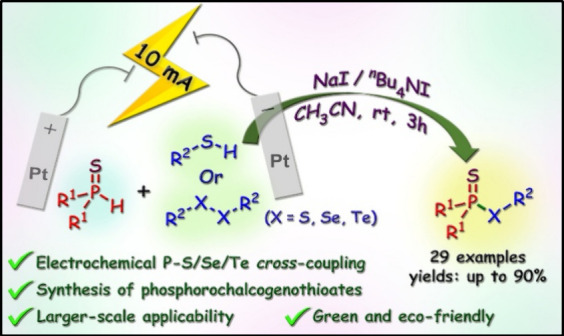

## 1 Introduction

Organophosphorus compounds represent a vital class of molecules in contemporary science, owing to their structural diversity and widespread utility in chemical synthesis, medicinal chemistry, and agricultural chemistry.^1^ Phosphorodithioates exhibit notable biological properties that make them valuable in both agricultural and pharmaceutical applications, serving as key components in pesticides and as functional groups in acetylcholinesterase inhibitors and antisense therapeutics.^2^ Phosphorodithioate-modified siRNA has demonstrated the potential to improve therapeutic efficacy in both in vitro and in vivo studies.^3^ Meanwhile, the notable bioactivity of organosulfur^4^ and organoselenium^5^ compounds has made them a central focus of research, encompassing studies on both natural sources and synthetic analogues in medicinal chemistry. Their wide-ranging applications encompass pharmaceuticals,^6^ agrochemicals,^7^ organic synthesis,^8^ and the design of functional and fluorescent materials,^9^ underscoring their essential role in modern chemical research and industrial innovation.^10^ Figure 1 illustrates several representative biologically active phosphorodithioate, phosphorothioate, and phosphoroselenoate derivatives.^11^

**Figure 1:**
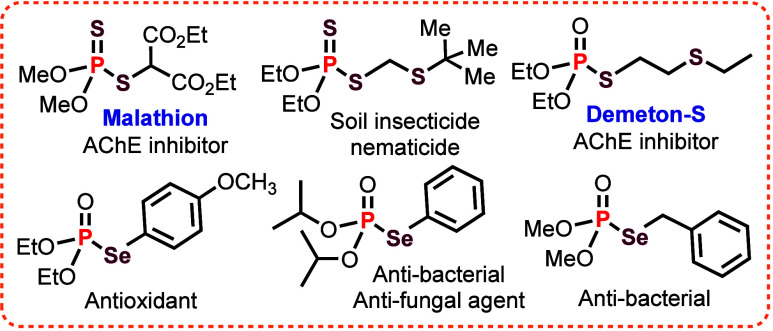
Biologically active phosphorodithioates, phosphorothioates, and phosphoroselenoates.

Cross-coupling reactions are indispensable tools for constructing complex molecular architectures with high efficiency, selectivity, and functional group tolerance. In this context, methodologies that enable the formation of P–S and P–Se bonds provide a powerful and versatile strategy for synthesizing organophosphorus-chalcogenide compounds. These transformations, often performed under mild and energy-efficient conditions, not only streamline the synthetic process but also enhance the biological properties of the resulting molecules, positioning them as promising candidates for medicinal and pharmaceutical applications.^12^ An organo­(thio)phosphorus compound containing sulfur, selenium, and phosphorus atoms was synthesized to investigate its distinct chemical and biological properties. The strategic incorporation of these heteroatoms imparts enhanced reactivity and potential multifunctionality. Such molecular frameworks are of considerable interest due to their broad applications in catalysis, medicinal chemistry, and materials science.^13^ Over the past few decades, various chalcogenation strategies for constructing phosphonothioates have been developed, encompassing nucleophilic, electrophilic, and radical pathways. In 2016, Xu et al.^14^ introduced a one-step method for synthesizing *S*-aryl phosphorothioates and *S*-aryl phosphorodithioates from aryl boronic acids, elemental sulfur, and P­(O)H compounds. This transformation proceeded efficiently under Cu­(OTf)_2_ catalysis with 2,2′-bipyridine as the ligand and triethylamine as an additive, affording the desired products in 20 h. Later, in 2022, Lee and co-workers^15^ developed a triethylamine-catalyzed, visible-light-mediated protocol enabling the synthesis of phosphorochalcogenoates, phosphorochalcogenothioates, and phosphinothioates within 16 h. More recently, Lee and co-workers^16^ reported a complementary approach for accessing phosphorochalcogenothioates under basic conditions using molecular oxygen as an external oxidant. Electrochemical organic synthesis has emerged as a powerful and sustainable approach in modern synthetic chemistry, enabling precise redox transformations under mild conditions, while minimizing the use of hazardous reagents. Its versatility and environmental friendliness make it increasingly valuable for constructing simple to complex molecules.^17^ To our delight,^18^ we have successfully developed an electrochemical phosphorochalcogenation method for the efficient synthesis of phosphorochalcogenothioates (**3**/**5/7**), overcoming the limitations of previously reported methodologies. This transformation proceeds via a direct coupling of substituted *O*,*O*-dialkyl phosphonothioates (**1**) with the corresponding thiols or diaryl diselenides or dipheny diteluride (**2**/**4/6**) in an undivided electrochemical cell. The reaction utilizes inexpensive and readily available NaI or *^n^* Bu_4_NI as the electrolyte and redox mediator in acetonitrile solvent under an ambient atmosphere. Performing the reaction with Pt/Pt electrodes and a constant direct current of 10 mA at room temperature for 3 h (Scheme 1) affords the desired products efficiently. This method offers several notable advantages: it accommodates a wide range of substrates, operates without the need for transition-metal catalysts or external oxidants, and proceeds smoothly at room temperature. The process is not only efficient—yielding products in good to excellent yields within a short time—but also scalable and environmentally friendly. Significantly, this work highlights the growing importance of electrochemical methods as a sustainable and versatile tool in modern organic synthesis.

**Scheme 1:**
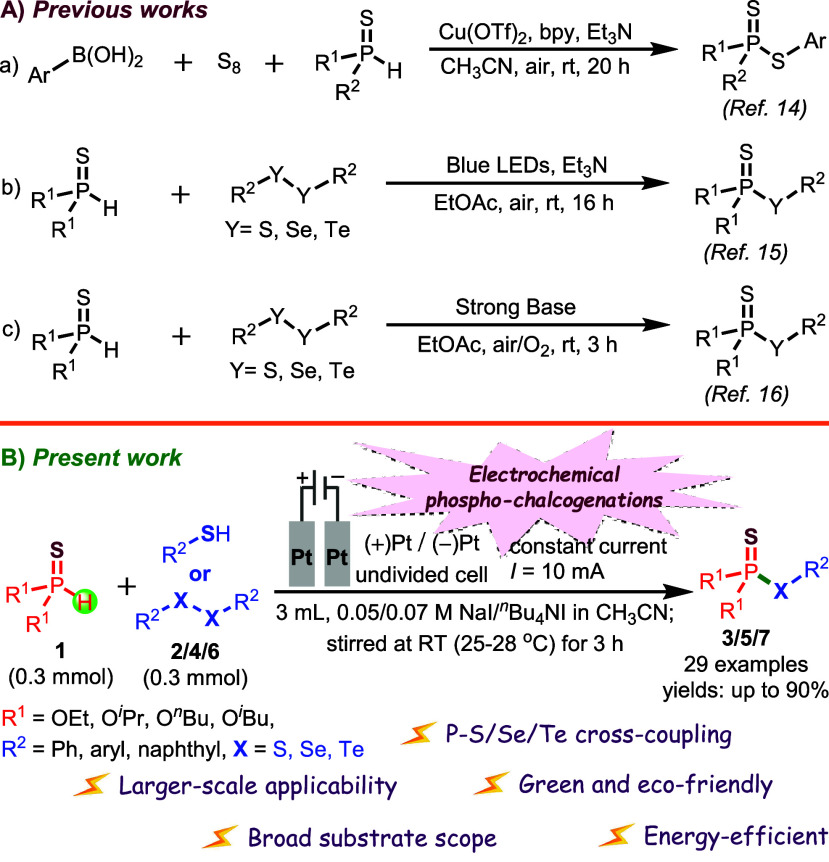
Electrochemical Synthesis of Phosphorochalcogenothioates: (A, a–c) Previous Works^14–16^ and (B) Present Work

## 2 Results and Discussion

To establish the optimal conditions for the electrochemical synthesis of phosphorochalcogenothioates via the chalcogenation of phosphonothioates, we began our investigation with a series of test reactions in which *O*,*O*-diethyl phosphonothioate (**1a**, 0.3 mmol) and benzenethiol (**2a**, 0.3 mmol) were employed as the dehydrogenative coupling partners. As an initial assessment, a mixture of **1a** and **2a** was stirred at room temperature under ambient conditions in acetonitrile (CH_3_CN), both without and with the application of a constant direct current (10 mA). In neither case was any conversion observed, even after 5 h (Table 1, entries 1–2). Since no product formation was observed under the initial conditions, we next evaluated the reaction of **1a** (0.3 mmol) with **2a** (0.3 mmol) in CH_3_CN (3 mL) containing NaI (0.05 M) as an electrolyte under a constant current of 10 mA, employing Pt/Pt electrodes in an undivided cell at room temperature. Notably, when the reaction was conducted for 3 h under different atmospheres, the desired product, *O*,*O*-diethyl *S*-phenyl phosphorodithioate (**3a**), was obtained in 83%, 83%, and 80% yield under air, O_2_, and N_2_, respectively (Table 1, entries 3–5). These results indicate that open-air conditions provide the most favorable atmosphere for this transformation. Next, varying the applied direct current in either direction resulted in decreased yields of **3a**. At 15 mA, the product was obtained in 81% yield, whereas reducing the current to 5 mA afforded only a 66% yield after 3 h (Table 1, entries 6 and 7). Subsequently, we investigated the influence of different electrode materials. However, alternative electrode pairs such as Pt/C, C/Pt, C/C, Pt/Cu, and Pt/Ni did not provide satisfactory results, affording **3a** in 61%, 67%, and 62% yields, respectively, while the Pt/Cu and Pt/Ni combinations delivered only trace amounts of product (Table 1, entries 8–12). We subsequently assessed the influence of the electrolyte concentration. An increase to 0.08 M delivered **3a** in 81% yield, while a decrease to 0.01 M reduced the yield to 66% (Table 1, entries 13–14). To assess the effect of solvent, additional reactions were performed in 1,2-dichloroethane (1,2-DCE), dimethyl sulfoxide (DMSO), and water while keeping all other parameters constant (10 mA current, Pt/Pt electrodes, 0.05 M NaI, open air, and room temperature). Under these conditions, **3a** was obtained in 43%, 41%, and trace yield, respectively, after 3 h (Table 1, entries 15–17). To further optimize the reaction conditions, the model reaction was examined by using various supporting electrolytes, including KI, NH_4_I, *^n^* Bu_4_NI, *^n^* Bu_4_NBr, *^n^* Bu_4_NCl, and *^n^* Bu_4_NBF_4_, each at a concentration of 0.05 M in 3 mL of acetonitrile. The reactions were conducted under galvanostatic conditions (Pt/Pt electrodes, 10 mA) in open air at ambient temperature (25–28 ^ο^C). Notably, only KI, *^n^* Bu_4_NCl, and *^n^* Bu_4_NBF_4_ furnished the desired product, giving **3a** in 52%, 25%, and 69% yield, respectively, whereas NH_4_I and *^n^* Bu_4_NI failed to produce **3a** even after 3 h (Table 1, entries 18–22). Ultimately, the optimized parameters for the electrochemical formation of phosphorochalcogenothioates were identified by employing *O*,*O*-diethyl phosphonothioate (**1a**, 0.3 mmol) in combination with benzenethiol (**2a**, 0.3 mmol) as the representative substrate pair. The transformation delivered the highest efficiency when conducted in an undivided electrochemical cell fitted with platinum plate electrodes at both the anodic and the cathodic positions. A 0.05 M NaI solution in CH_3_CN (3 mL) served as the electrolyte system, and electrolysis under open-air conditions at ambient temperature (25–28 ^ο^C) with a maintained direct current of 10 mA enabled effective product formation. Under these optimized conditions, *O*,*O*-diethyl *S*-phenyl phosphorodithioate (**3a**) was isolated in 83% yield after 3 h of electrolysis (Table 1, entry 3). A detailed overview of all experimental trials conducted for this electrosynthetic transformation is presented in Table 1. Comprehensive spectroscopic examination, including ^1^H NMR, ^13^C NMR, ^31^P NMR, and HRMS, was employed to verify the identity of compound **3a**. The resulting physical and spectral data closely matched the values previously described in the literature.^15^

**Table 1:**
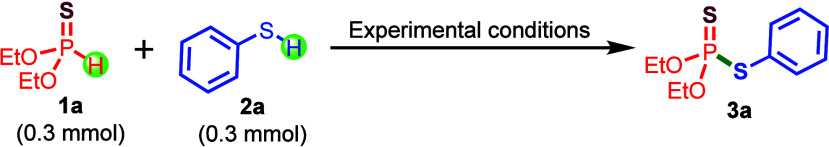
Optimization of Reaction Conditions for the Synthesis of *O*,*O*-Diethyl *S*-Phenyl Phosphorodithioate (**3a**)­^a^,^b^

**entry**	**electrolyte (M)**	**cell** (+/−)	**solvent** (5 mL)	**atmosphere**	**current (mA)**	**time (h)**	**yield (%)** ^a^,^b^
1			CH_3_CN	air		5	
2		Pt**/**Pt	CH_3_CN	air	10	5	
**3**	**NaI (0.05)**	**Pt/Pt**	**CH**_**3**_ **CN**	**air**	**10**	**3**	**83**
4	NaI (0.05)	Pt**/**Pt	CH_3_CN	O_2_	10	3	83
5	NaI (0.05)	Pt**/**Pt	CH_3_CN	N_2_	10	3	80
6	NaI (0.05)	Pt**/**Pt	CH_3_CN	air	15	3	81
7	NaI (0.05)	Pt**/**Pt	CH_3_CN	air	5	3	66
8	NaI (0.05)	Pt**/**C	CH_3_CN	air	10	3	71
9	NaI (0.05)	C**/**Pt	CH_3_CN	air	10	3	67
10	NaI (0.05)	C**/**C	CH_3_CN	air	10	3	62
11	NaI (0.05)	Pt**/**Cu	CH_3_CN	air	10	3	trace
12	NaI (0.05)	Pt**/**Ni	CH_3_CN	air	10	3	trace
13	NaI (0.08)	Pt**/**Pt	CH_3_CN	air	10	3	81
14	NaI (0.01)	Pt**/**Pt	CH_3_CN	air	10	3	66
15	NaI (0.05)	Pt**/**Pt	DCE	air	10	3	41
16	NaI (0.05)	Pt**/**Pt	DMSO	air	10	3	43
17	NaI (0.05)	Pt**/**Pt	H_2_O	air	10	3	trace
18	KI (0.05)	Pt**/**Pt	CH_3_CN	air	10	3	52
19	NH_4_I (0.05)	Pt**/**Pt	CH_3_CN	air	10	3	trace
20	*^n^* Bu_4_NI (0.05)	Pt**/**Pt	CH_3_CN	air	10	3	
21	*^n^* Bu_4_NBr (0.05)	Pt**/**Pt	CH_3_CN	air	10	3	25
22	*^n^* Bu_4_NBF_4_ (0.05)	Pt**/**Pt	CH_3_CN	air	10	3	69

a Reaction conditions: A mixture of *O*,*O*-diethyl phosphonothioate (**1a**; 0.3 mmol) and benzenethiol (**2a**; 0.3 mmol) dissolved in various electrolyte solutions and subjected to electrolysis at a constant current in an undivided cell with a 0.5 cm electrode gap at room temperature (25–28 ^ο^C). Electrode size: Pt, C, Cu, and Ni plates: 0.7 cm × 0.7 cm × 0.2 cm.

b Isolated yields. M = molarity.

Having established the optimized reaction conditions, we next investigated the substrate scope of this electrochemical cross-dehydrogenative phosphorochalcogenation. Reactions of *O*,*O*-diethyl phosphonothioate (**1a**, 0.3 mmol) with representative electron-donating aromatic thiols, including 4-methylbenzenethiol (**2b**, 0.3 mmol) and 4-methoxybenzenethiol (**2c**, 0.3 mmol), were conducted. Both reactions proceeded efficiently under the standard conditions, affording the corresponding products *O*,*O*-diethyl *S*-(*p*-tolyl) phosphorodithioate (**3b**) and *O*,*O*-diethyl *S*-(4-methoxyphenyl) phosphorodithioate (**3c**) in excellent yields of 75% and 80%, respectively, within 3 h (Table 2, compounds **3b**–**3c**). Encouraged by these results, the protocol was further applied to three additional cross-dehydrogenative phosphorochalcogenation reactions of *O*,*O*-diethyl phosphonothioate (**1a**, 0.3 mmol) with aromatic thiols bearing halogen substituents: 4-fluorobenzenethiol (**2d**, 0.3 mmol), 4-chlorobenzenethiol (**2e**, 0.3 mmol), and 4-bromobenzenethiol (**2f**, 0.3 mmol). All reactions proceeded smoothly under the optimized conditions, providing the corresponding products *O*,*O*-diethyl *S*-(4-fluorophenyl) phosphorodithioate (**3d**), *O*,*O*-diethyl *S*-(4-chlorophenyl) phosphorodithioate (**3e**), and *O*,*O*-diethyl *S*-(4-bromophenyl) phosphorodithioate (**3f**) in 62%, 85%, and 71% yield, respectively, within 3 h (Table 2, compounds **3d**–**3f**). To further evaluate the generality of this electrochemical protocol, reactions were carried out with heterocyclic and aliphatic thiols, including thiophene-2-thiol (**2g**, 0.3 mmol) and hexane-1-thiol (**2h**, 0.3 mmol), in combination with *O*,*O*-diethyl phosphonothioate (**1a**, 0.3 mmol) under the standard reaction conditions. Both reactions proceeded smoothly, furnishing the corresponding products *O*,*O*-diethyl *S*-(thiophen-2-yl) phosphorodithioate (**3g**) and *O*,*O*-diethyl *S*-hexyl phosphorodithioate (**3h**) in good yields of 51% and 58%, respectively, within 3 h (Table 2, compounds **3g**–**3h**). To further assess the general applicability of this methodology, we examined the reaction using a substituted phosphonothioate. *O*,*O*-Diisopropyl phosphonothioate (**1b**, 0.3 mmol) was coupled with phenylmethanethiol (**2i**, 0.3 mmol) under the optimized conditions. The reaction proceeded efficiently, yielding *S*-benzyl *O*,*O*-diisopropyl phosphorodithioate (**3i**) in an excellent 83% yield within 3 h (Table 2, compound **3i**). In a final series of experiments, we extended the methodology to two additional substituted phosphonothioates—*O*,*O*-dibutyl phosphonothioate (**1c**, 0.3 mmol) and *O*,*O*-diisobutyl phosphonothioate (**1d**, 0.3 mmol). These substrates were reacted with a set of aromatic thiols (**2b**, **2d**, **2e**, and **2f**; 0.3 mmol each), bearing substituents such as −CH_3_, −F, −Cl, and −Br, respectively. Under the optimized reaction conditions, five distinct phosphorodithioate products were obtained: *O*,*O*-dibutyl *S*-(*p*-tolyl) phosphorodithioate (**3j**), *O*,*O*-dibutyl *S*-(4-fluorophenyl) phosphorodithioate (**3k**), *O*,*O*-dibutyl *S*-(4-chlorophenyl) phosphorodithioate (**3l**), *O*,*O*-dibutyl *S*-(4-bromophenyl) phosphorodithioate (**3m**), and *O*,*O*-diisobutyl *S*-(*p*-tolyl) phosphorodithioate (**3n**). These transformations proceeded smoothly, delivering the desired products in good to excellent yields (60%–79%) within 3 h (Table 2, compounds **3j**–**3n**).

**Table 2:**
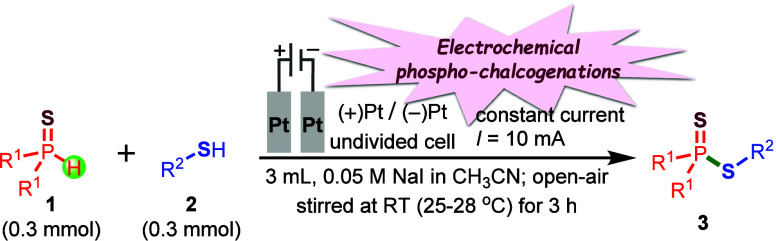

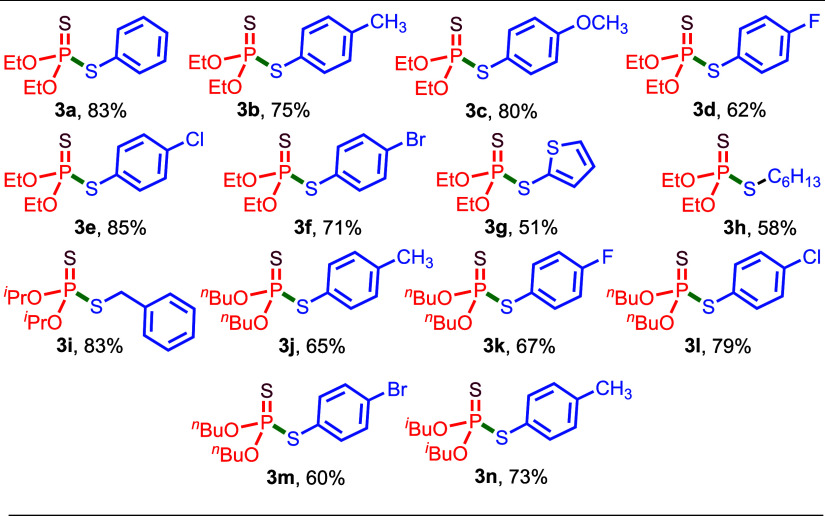
Substrate Scope for the Synthesis of Phosphorodithioate^a^,^b^

a Reaction conditions: A mixture of phosphonothioates (**1**; 0.3 mmol) and thiols (**2**; 0.3 mmol) dissolved in 0.05 M NaI in CH_3_CN (3 mL) at a constant current (*I* = 10 mA) in an undivided Pt/Pt cell with a 0.5 cm electrode gap at room temperature (25–28 ^ο^C) in open-air atmosphere for 3h.

b Isolated yields.

After successfully exploring the substrate scope of phosphorodithioate synthesis, we were motivated to extend the methodology toward the preparation of phosphoroselenothioates via an electrochemical approach. Initially, the reaction between *O*,*O*-diethyl phosphonothioate (**1a**, 0.3 mmol) and 1,2-diphenyldiselenide (**4a**, 0.3 mmol) was examined by using a Pt/Pt electrode pair under a constant direct current of 10 mA in the presence of various supporting electrolytes. Subsequently, the optimized parameters for the electrochemical synthesis of phosphoroselenothioates were established using the same substrate combination as a model system. The reaction exhibited the highest efficiency when conducted in an undivided electrochemical cell equipped with platinum plate electrodes at both the anode and cathode. A 0.07 M *^n^* Bu_4_NI solution in CH_3_CN (3 mL) served as the electrolyte medium, and electrolysis performed under open-air conditions at ambient temperature (25–28 ^ο^C) with a constant current of 10 mA efficiently afforded the desired product efficiently. Under these optimized conditions, *O*,*O*-diethyl *Se*-phenyl phosphoroselenothioate (**5a**) was isolated in 86% yield after 3 h of electrolysis (Table 3, entry 6). A comprehensive summary of all of the experimental trials performed for this electrosynthetic transformation is provided in Table 3.

**Table 3:**
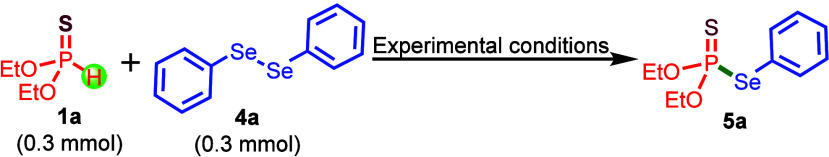
Optimization of Reaction Conditions for the Synthesis of *O*,*O*-Diethyl *Se*-Phenyl Phosphoroselenothioate (**5a**)

**entry**	**electrolyte (M)**	**cell** (+/−)	**solvent** (5 mL)	**atmosphere**	**current (mA)**	**time (h)**	**yield (%)** ^a^,^b^
1			CH_3_CN	air		5	
2		Pt**/**Pt	CH_3_CN	air	10	5	
3	NaI (0.07)	Pt**/**Pt	CH_3_CN	air	10	3	60
4	KI (0.07)	Pt**/**Pt	CH_3_CN	air	10	3	47
5	NH_4_I (0.07)	Pt**/**Pt	CH_3_CN	air	10	3	trace
**6**	^*n*^ **Bu**_**4**_ **NI (0.07)**	**Pt/Pt**	**CH**_**3**_ **CN**	**air**	**10**	**3**	**86**
7	*^n^* Bu_4_NBr (0.07)	Pt**/**Pt	CH_3_CN	air	10	3	27
8	*^n^* Bu_4_NBF_4_ (0.07)	Pt**/**Pt	CH_3_CN	air	10	3	66
9	*^n^* Bu_4_NI (0.07)	Pt**/**Pt	DCE	air	10	3	54
10	*^n^* Bu_4_NI (0.07)	Pt**/**Pt	DMSO	air	10	3	51
11	*^n^* Bu_4_NI (0.07)	Pt**/**Pt	H_2_O	air	10	3	trace
12^c^	*^n^* Bu_4_NI (0.07)	Pt**/**Pt	CH_3_CN	air	10	3	71

a Reaction conditions: A mixture of *O*,*O*-diethyl phosphonothioate (**1a**; 0.3 mmol) and 1,2-diphenyldiselane (**2a**; 0.3 mmol) dissolved in various electrolyte solutions and subjected to electrolysis at a constant current in an undivided cell with a 0.5 cm electrode gap at room temperature (25–28 ^ο^C). Electrode size: Pt, C, Cu, and Ni plates: 0.7 cm × 0.7 cm × 0.2 cm.

b Isolated yields.

c **2a**: 0.15 mmol. M = molarity.

With the optimized reaction conditions in hand, we next examined the substrate scope of this electrochemical synthesis of phosphoroselenothioates. *O*,*O*-Diethyl phosphonothioate (**1a**, 0.3 mmol) was reacted with a series of electron-donating and electron-withdrawing aromatic and heteroaromatic diaryl diselenides bearing −CH_3_, −OCH_3_, −F, −Cl, naphthyl, and thiophenyl substituents (0.3 mmol each, **4b**–**4g**). All reactions proceeded smoothly under the standard conditions, affording the corresponding products *O*,*O*-diethyl *Se*-(*p*-tolyl) phosphoroselenothioate (**5b**), *O*,*O*-diethyl *Se*-(4-methoxyphenyl) phosphoroselenothioate (**5c**), *O*,*O*-diethyl *Se*-(4-fluorophenyl) phosphoroselenothioate (**5d**), *Se*-(4-chlorophenyl) *O*,*O*-diethyl phosphoroselenothioate (**5e**), *O*,*O*-diethyl *Se*-(naphthalen-1-yl) phosphoroselenothioate (**5f**), and *O*,*O*-diethyl *Se*-(thiophen-2-yl) phosphoroselenothioate (**5g**) in good to excellent yields (69%–80%) within 3 h (Table 4, compounds **5b**–**5g**). Encouraged by these results, we next evaluated the generality of the method by employing various substituted phosphonothioates. Accordingly, *O*,*O*-diisopropyl phosphonothioate (**1b**, 0.3 mmol), *O*,*O*-dibutyl phosphonothioate (**1c**, 0.3 mmol), and *O*,*O*-diisobutyl phosphonothioate (**1d**, 0.3 mmol) were coupled with a range of electron-donating and electron-withdrawing diaryl diselenides under the optimized electrochemical conditions. All reactions proceeded efficiently, affording the corresponding phosphoroselenothioate products—*O*,*O*-diisopropyl *Se*-phenyl phosphoroselenothioate (**5h**), *O*,*O*-dibutyl *Se*-phenyl phosphoroselenothioate (**5i**), *O*,*O*-dibutyl *Se*-(*p*-tolyl) phosphoroselenothioate (**5j**), *O*,*O*-dibutyl *Se*-(4-fluorophenyl) phosphoroselenothioate (**5k**), *O*,*O*-dibutyl *Se*-(4-chlorophenyl) phosphoroselenothioate (**5l**), and *O*,*O*-diisobutyl *Se*-phenyl phosphoroselenothioate (**5m**)—in good to excellent isolated yields (68%–90%) within 3 h (Table 4, compounds **5h**–**5m**). Under the same optimized reaction conditions, we next investigated the substrate scope of this electrochemical phosphorochalcogenation using diphenyl ditelluride (**6**). Reactions of diphenyl ditelluride (**6**, 0.3 mmol) with representative phosphonothioates, including *O*,*O*-diisopropyl phosphonothioate (**1b**, 0.3 mmol) and *O*,*O*-dibutyl phosphonothioate (**1c**, 0.3 mmol), were conducted. Both reactions proceeded smoothly under the standard conditions, affording the corresponding products *O*,*O*-diisopropyl *Te*-phenyl phosphorotellurothioate (**7a**) and *O*,*O*-dibutyl *Te*-phenyl phosphorotellurothioate (**7b**), in yields of 62% and 76%, respectively, within 3 h (Table 4, compounds **7a** and **7b**). All synthesized compounds **3**, **5** and **7** (**3a**–**3n, 5a**–**5m**, and **7a**–**7b**; 29 derivatives in total) were purified by flash column chromatography (see Section 4). Each compound was fully characterized by comprehensive spectroscopic analysis, including ^1^H NMR, ^13^C NMR, ^31^P NMR (for **3a**, **5a**, and **7a**), ^19^F NMR (for fluorinated derivatives: **3d**, **3k**, **5d**, and **5k**), and HRMS (for new compounds) (see Section 4).

**Table 4:**
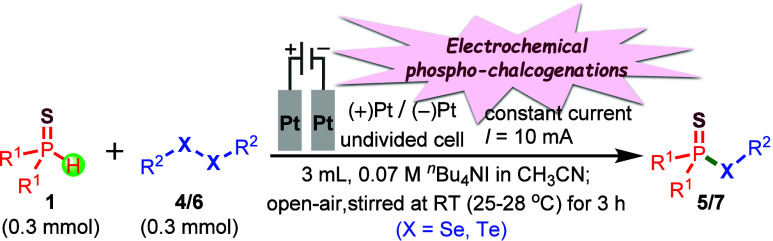

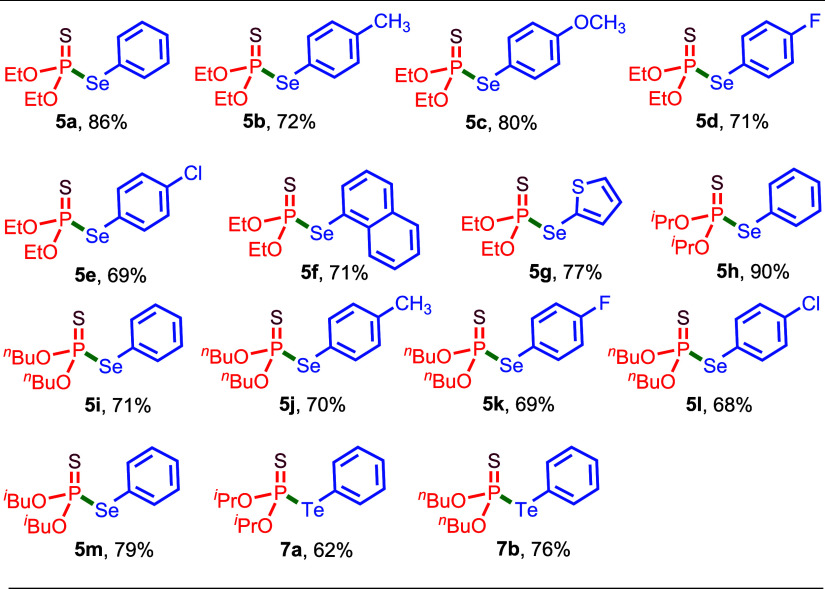
Substrate Scope for the Synthesis of Phosphoroselenothioate^a^,^b^

a Reaction conditions: A mixture of phosphonothioates (**1**; 0.3 mmol) and diaryl diselenides (**4/6**; 0.3 mmol) dissolved in 0.05 M *^n^* Bu_4_NI in CH_3_CN (3 mL) at a constant current (*I* = 10 mA) in an undivided Pt/Pt cell with a 0.5 cm electrode gap at room temperature (25–28 ^ο^C) in open-air atmosphere for 3 h.

b Isolated yields.

We further assessed the scalability of this electrochemical phosphorochalcogenation protocol by conducting reactions on a larger scale (3.0 and 5.0 mmol; 10-fold and 15-fold increase) using the model substrate pairs to synthesize compounds **3a** and **5a** (seeScheme [Fig sch2]). The reactions proceeded smoothly under the optimized conditions, affording **3a** in 76% and 75% yield and **5a** in 79% and 78% yield. Although the yields were slightly lower compared with those obtained on the 0.3 mmol scale, the process remained efficient, with only a modest extension in reaction time required. This demonstrates the method’s practical scalability and efficiency.

**Scheme 2:**
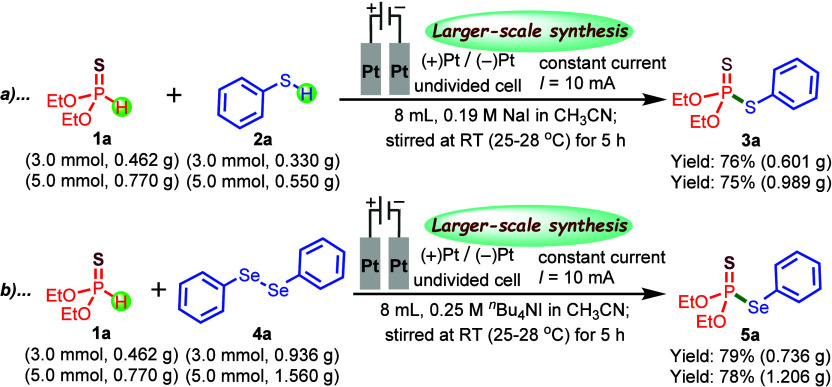
(a, b) Larger-Scale Synthetic Applications

At this stage, we sought to elucidate the possible mechanism underlying the electrochemical phosphorochalcogenation of substituted *O*,*O*-dialkyl phosphonothioates with the corresponding thiols or diaryl diselenides. To obtain a more detailed understanding of the reaction mechanism, we investigated the electrochemical phosphorochalcogenation between *O*,*O*-diethyl phosphonothioate (**1a**) and 1,2-diphenyldisulfane (**8a**) in place of the simple thiol (**2a**) under the optimized conditions. Remarkably, we isolated a nearly identical yield (81%) within the same reaction time, further supporting the proposed mechanistic rationale (Scheme 3a). Further mechanistic insights were obtained through a series of control experiments using the model reaction (Scheme 3b). When the reaction was carried out under the standard conditions in the presence of various radical-trapping agents, including TEMPO, BHT, and *p*-benzoquinone, the formation of target product **3a** was completely suppressed (Scheme 3b). These observations strongly suggest that the transformation proceeds through a radical pathway. To our delight, HRMS analysis confirmed the formation of the corresponding TEMPO and BHT adducts **9** and **10** (Scheme 3b), thereby providing additional support for the involvement of radical intermediates.

**Scheme 3:**
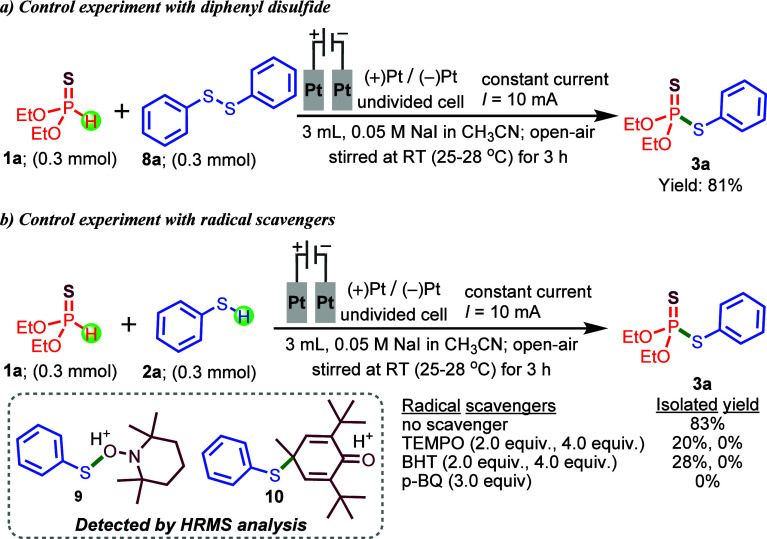
Control Experiments: (a) Diphenyl Disulfide and (b) Radical Scavengers

Based on literature support,^8a,19^ detailed control experiments, and the cyclic voltammetry studies of the reaction components (Figure 2), we propose a plausible mechanism for the electrochemical phosphorochalcogenation, as illustrated in Scheme 4. Cyclic voltammetry (Figure 2a–d) of model substrate **1a** showed no discernible oxidation peaks, whereas **2a** and **4a** exhibited an oxidation peak at +1.55 V (vs. Ag/Ag^+^) and +1.58 V (vs. Ag/Ag^+^), indicating a one-electron oxidation at the anode surface to generate thiol radical **2′/4′** (Scheme 4). In contrast, NaI displayed two distinct anodic peaks at +0.76 V and +0.98 V (vs. Ag/Ag^+^), and *^n^* Bu_4_NI shows anodic oxidation peaks at +0.81 V and +1.04 V (vs. Ag/Ag^+^), which confirms that these species undergo electrochemical oxidation (Scheme 4, Paths A and B). Upon oxidation, the iodide ion generates iodine radicals. The resulting thiol radical (**2′**) or aryl selenium radical (**4′**) then undergoes radical recombination with the iodine radical to form the corresponding intermediates **2′-I** or **4′-I**, respectively (Scheme 4, Paths A and B). From the cyclic voltammetry study, intermediates **2′-I** and **4′-I** exhibit strong oxidation peaks at +0.99 V and +1.03 V (vs Ag/Ag^+^), respectively. These signals provide clear evidence for the formation of the corresponding intermediate species (Figure 2a,b). On the other hand, the phosphonothioates (**1**) undergo tautomerization to form the corresponding species **1′** in acetonitrile. This highly reactive intermediate readily attacks **2′-I** or **4′-I**, leading to the elimination of iodide and a proton to afford the final products **3**/**5**. At the cathode surface, electrochemical reduction of protons generates molecular hydrogen (Scheme 4, Path A). In Method B, proton reduction similarly produces hydrogen, while an additional one-electron reduction of species **4″** regenerates the starting diaryl diselenide **4** (Scheme 4, Path B).

**Figure 2:**
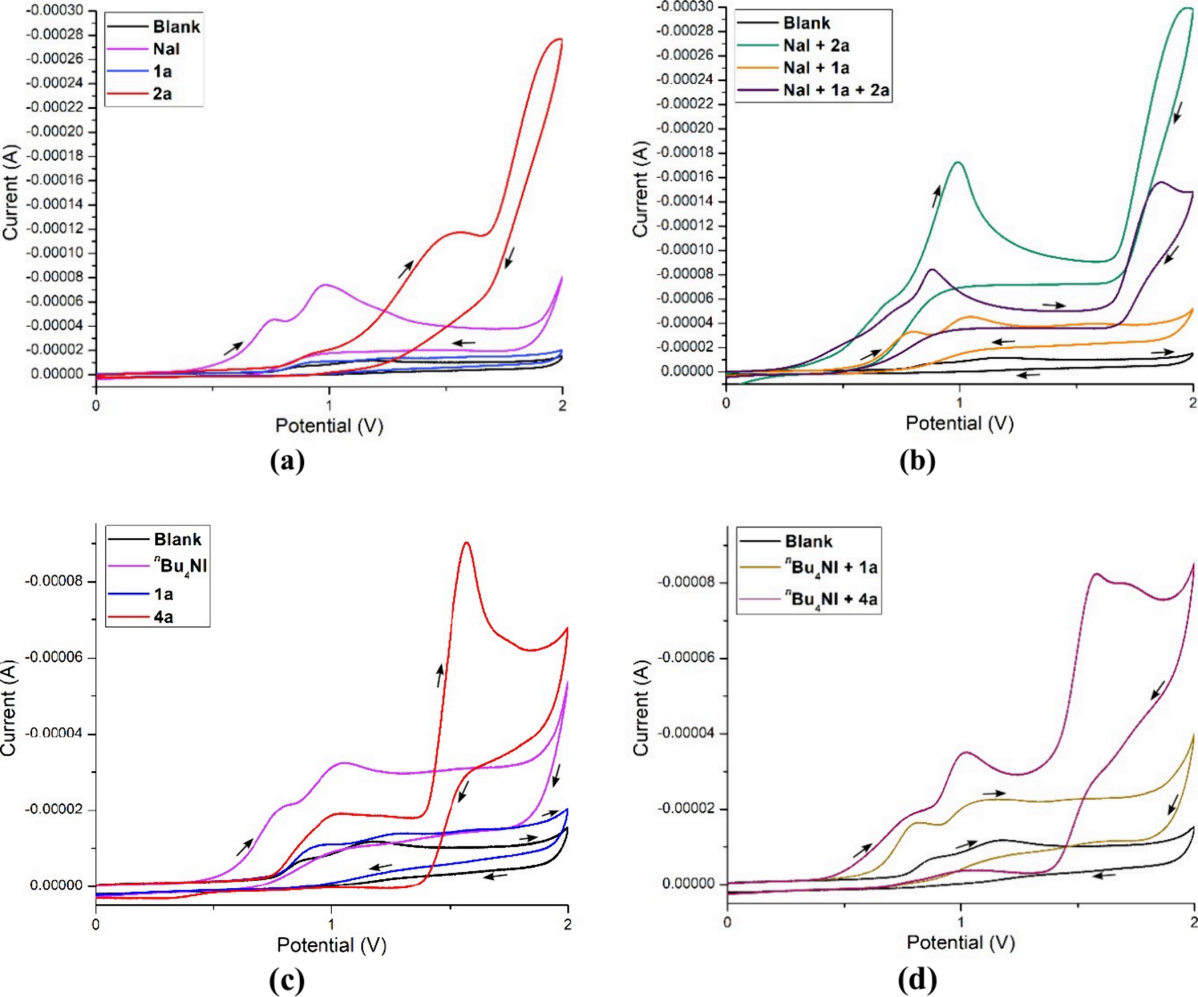
(a–d) Cyclic voltammetry diagrams (CV plotting convention: IUPAC; potential scan ranged: from 0 to +2.0 V; direction of scan: + direction; scan rate: 0.1 V/s).

**Scheme 4:**
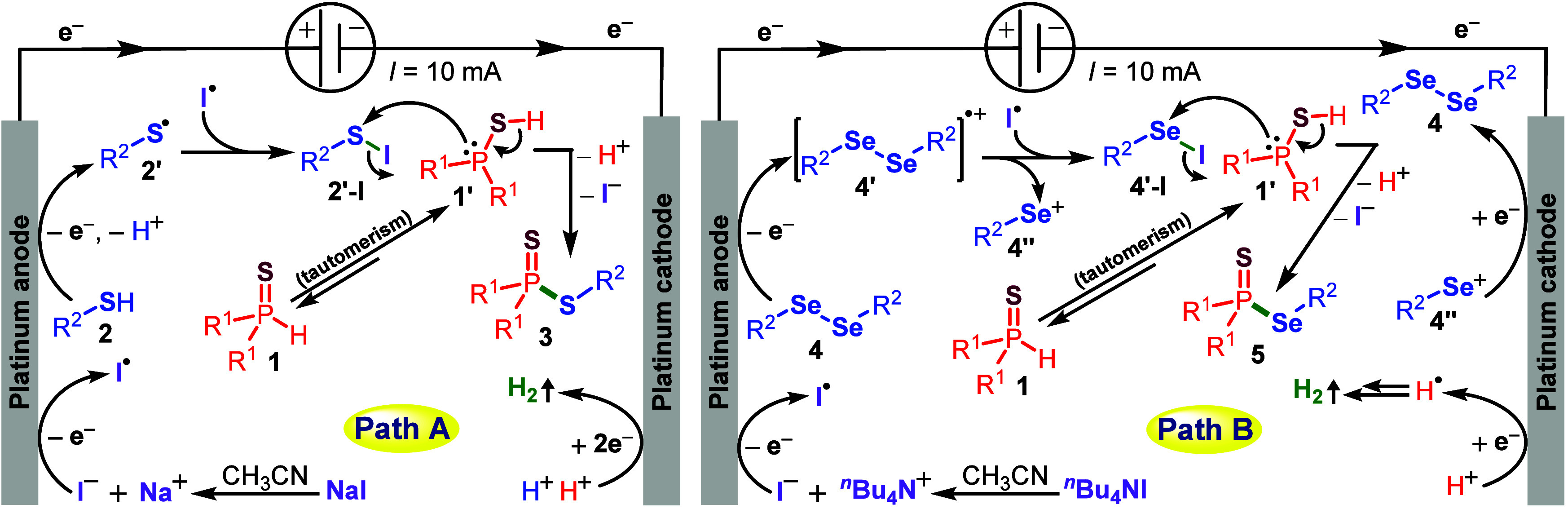
Proposed Mechanism for Electrochemical Phosphorochalcogenation

## 3 Conclusions

In summary, we have developed a robust and environmentally conscious electrosynthetic protocol for the efficient construction of structurally diverse and biologically relevant phosphorochalcogenated compounds. The method relies on the electrochemical cross-coupling of phosphonothioates with thiols or diaryl diselenides, enabled by inexpensive NaI or *^n^* Bu_4_NI serving as both a green electrolyte and a redox mediator. Conducted in CH_3_CN at room temperature under a constant current using Pt/Pt electrodes, this protocol offers multiple advantages, including mild and energy-efficient conditions, transition-metal-free operation, short reaction times, high to excellent yields, broad substrate scope, and large-scale applicability. These features collectively highlight the operational simplicity and environmental sustainability of this electrosynthetic approach, underscoring its potential value for future applications in synthetic and medicinal chemistry. Ongoing work in our laboratory is aimed at further expanding the scope of such sustainable electrochemical functionalization strategies.

## 4 Experimental Section

### 4.1 General Method

All solvents used in this study were distilled and dried prior to use following standard procedures. ^1^H, ^13^C, ^19^F, and ^31^P NMR spectra were recorded on a Varian Unity Inova-600 or a Varian Mercury-400 NMR spectrometer using CDCl_3_ as the solvent. Chemical shifts (δ) are reported in parts per million relative to the internal standard, TMS. Signal multiplicities are denoted as s (singlet), d (doublet), t (triplet), and m (multiplet), and coupling constants (*J*) are reported in Hz. Mass spectrometry analyses were carried out on a Jeol JMS-HX 110 spectrometer. Liquid chromatography (LC) analyses were performed using a SHIMADZU LC-2050C 3D system, while LC-mass spectrometry (LC-MS) data were collected on a DPiMS-2020. Liquid chromatography-tandem mass spectrometry (LC-MS/MS) was performed using an LC-8045 system. Protein analyses were conducted on a Waters SYNAPT HDMS Q-TOF mass spectrometer (ESI-Q-TOF). Cyclic voltammetry measurements were performed on a CHI Instruments 750A potentiostat using acetonitrile as the solvent. Melting points were determined using a Büchi 535 melting point apparatus and are reported as uncorrected. Thin-layer chromatography (TLC) was carried out on silica gel 60 F_254_ plates (Merck). Electrochemical reactions were conducted in an undivided cell equipped with platinum electrodes (IKA), with dimensions of 0.7 × 0.7 × 0.2 cm for small-scale reactions and 2.8 × 0.7 × 0.2 cm for larger-scale reactions. A GW Instek GPS-2303 laboratory DC power supply (350 W, 450 VA, 50/60 Hz) was used as the power source.

### 4.2 General Procedure for the Synthesis of Compounds **3**/**5**/**7**

An oven-dried 10 mL glass vessel was sequentially charged with the substituted *O*,*O*-dialkyl phosphonothioates (**1**, 0.3 mmol, 1.0 equiv), the corresponding thiols or diaryl diselenides or diphenyl ditelluride (**2**/**4/6**, 0.3 mmol, 1.0 equiv), and 3 mL of a 0.05/0.07 M NaI/*^n^* Bu_4_NI (0.15 mmol, 0.5 equiv./0.20 mmol, 0.7 equiv) electrolyte solution in acetonitrile (CH_3_CN, 3 mL), along with a magnetic stir bar. Platinum plates (working dimensions: 0.7 × 0.7 × 0.2 cm) were used as both the anode and cathode, positioned 0.5 cm apart to form an undivided electrochemical cell. A constant direct current of 10 mA was applied to the stirred reaction mixture at room temperature for 3 h. Reaction progress was monitored periodically by thin-layer chromatography (TLC). Upon completion, 20 mL of a 3:1 (v/v) ethyl acetate/water mixture was added, and the mixture was transferred to a separatory funnel and shaken vigorously. The organic layer was separated, dried over anhydrous sodium sulfate, and concentrated under reduced pressure. The crude product was purified by column chromatography using EtOAc–hexane mixtures as eluents to afford desired products **3**/**5/7** (**3a**–**3n, 5a**–**5m**, and **7a**–**7b**). In total, 29 derivatives were synthesized and fully characterized by detailed spectroscopic analysis, including ^1^H NMR, ^13^C NMR, ^31^P NMR (for **3a**, **5a,** and **7a**), ^19^F NMR (for **3d**, **3k**, **5d**, and **5k**), and HRMS (for new compounds).

### 4.3 Physical and Spectral Data of the Synthesized Compounds **3**, **5**, and **7** Are Given Below

#### 4.3.1 *O*,*O*-Diethyl *S*-Phenyl Phosphorodithioate (**3a**)^15^

Following the general procedure, using *O*,*O*-diethyl phosphonothioate (**1a**; 0.3 mmol, 0.047 g), thiophenol (**2a**, 0.3 mmol, 0.033 g), NaI (0.05 M, 0.023 g), and 3 mL of CH_3_CN then purified by column chromatography (SiO_2_, 10–15% ethyl acetate in hexanes) to provide **3a** as a colorless oil; yield: 83% (0.065 g; 0.3 mmol scale). ^1^H NMR (CDCl_3_, 400 MHz): δ = 7.52 (d, *J* = 6.4 Hz, 2H), 7.37 (t, *J* = 6.4 Hz, 3H), 4.31–4.14 (m, 4H), 1.33–1.29 (m, 6H) ppm. ^13^C­{^1^H} NMR (CDCl_3_, 100 MHz): δ = 134.9 (d, *J*_CP_ = 5 Hz, 2*C*), 129.4 (d, *J*_CP_ = 2 Hz, 3*C*), 128.4 (d, *J*_CP_ = 7 Hz), 64.3 (d, *J*_CP_ = 6 Hz), 15.9 (d, *J*_CP_ = 9 Hz) ppm. ^31^P NMR (CDCl_3_, 162 MHz): δ = 88.68 ppm. HRMS (EI) calcd for C_10_H_15_O_2_PS_2_H^+^ [M + H]^+^ *m*/*z* 263.0324, found *m*/*z* 263.0321.

#### 4.3.2 *O*,*O*-Diethyl *S*-(*p*-Tolyl) Phosphorodithioate (**3b**)^15^

Following the general procedure, using *O*,*O*-diethyl phosphonothioate (**1a**; 0.3 mmol, 0.047 g), 4-methylbenzenethiol (**2b**, 0.3 mmol, 0.038 g), NaI (0.05 M, 0.023 g), and 3 mL of CH_3_CN then purified by column chromatography (SiO_2_, 10–15% ethyl acetate in hexanes) to provide **3b** as a colorless oil; yield: 75% (0.062 g; 0.3 mmol scale). ^1^H NMR (CDCl_3_, 400 MHz): δ = 7.38 (dd, *J* = 8.4 and 2.0 Hz, 2H), 7.16 (d, *J* = 8.0 Hz, 2H), 4.29–4.11 (m, 4H), 2.34 (s, 3H), 1.31 (t, *J* = 7.2 Hz, 6H) ppm. ^13^C­{^1^H} NMR (CDCl_3_, 100 MHz): δ = 139.6 (d, *J*_CP_ = 3 Hz), 134.8 (d, *J*_CP_ = 4 Hz, 2*C*), 130.1 (d, *J*_CP_ = 3 Hz, 2*C*), 124.6 (d, *J*_CP_ = 7 Hz), 64.1 (d, *J*_CP_ = 6 Hz, 2*C*), 21.3, 15.8 (d, *J*_CP_ = 8 Hz, 2*C*) ppm.

#### 4.3.3 *O*,*O*-Diethyl *S*-(4-Methoxyphenyl) Phosphorodithioate (**3c**)^16b^

Following the general procedure, using *O*,*O*-diethyl phosphonothioate (**1a**; 0.3 mmol, 0.047 g), 4-methoxybenzenethiol (**2c**, 0.3 mmol, 0.042 g), NaI (0.05 M, 0.023 g), and 3 mL of CH_3_CN then purified by column chromatography (SiO_2_, 10–15% ethyl acetate in hexanes) to provide **3c** as a colorless oil; yield: 80% (0.070 g; 0.3 mmol scale). ^1^H NMR (CDCl_3_, 400 MHz): δ = 7.43–7.39 (m, 2H), 6.89–6.86 (m, 2H), 4.28–4.08 (m, 4H), 3.80 (s, 3H), 1.33–1.29 (m, 6H) ppm. ^13^C­{^1^H} NMR (CDCl_3_, 100 MHz): δ = 160.8 (d, *J*_CP_ = 4 Hz), 136.6 (d, *J*_CP_ = 5 Hz, 2*C*), 118.6 (d, *J*_CP_ = 7 Hz), 114.9 (d, *J*_CP_ = 3 Hz, 2*C*), 64.2 (d, *J*_CP_ = 6 Hz, 2*C*), 55.4, 15.89 (d, *J*_CP_ = 9 Hz, 2*C*) ppm.

#### 4.3.4 *O*,*O*-Diethyl *S*-(4-Fluorophenyl) Phosphorodithioate (**3d**)^15^

Following the general procedure, using *O*,*O*-diethyl phosphonothioate (**1a**; 0.3 mmol, 0.047 g), 4-fluorobenzenethiol (**2d**, 0.3 mmol, 0.039 g), NaI (0.05 M, 0.023 g), and 3 mL of CH_3_CN then purified by column chromatography (SiO_2_, 10–15% ethyl acetate in hexanes) to provide **3d** as a colorless oil; yield: 62% (0.052 g; 0.3 mmol scale). ^1^H NMR (CDCl_3_, 400 MHz): δ = 7.52–7.46 (m, 2H), 7.09–7.03 (m, 2H), 4.29–4.11 (m, 4H), 1.33–1.29 (m, 6H) ppm. ^13^C­{^1^H} NMR (CDCl_3_, 100 MHz): δ = 163.6 (dd, *J*_CF and CP_ = 249 and 4 Hz), 137.1 (dd, *J*_CF and CP_ = 8 and 4 Hz, 2*C*), 123.6 (dd, *J*_CF and CP_ = 8 and 4 Hz), 116.6 (dd, *J*_CF and CP_ = 22 and 3 Hz, 2*C*), 64.4 (d, *J*_CP_ = 6 Hz), 15.9 (d, *J*_CP_ = 9 Hz) ppm. ^19^F NMR (CDCl_3_, 376 MHz): δ = −111.45 ppm.

#### 4.3.5 *S*-(4-Chlorophenyl) *O*,*O*-Diethyl Phosphorodithioate (**3e**)^16b^

Following the general procedure, using *O*,*O*-diethyl phosphonothioate (**1a**; 0.3 mmol, 0.047 g), 4-chlorobenzenethiol (**2e**, 0.3 mmol, 0.044 g), NaI (0.05 M, 0.023 g), and 3 mL of CH_3_CN then purified by column chromatography (SiO_2_, 10–15% ethyl acetate in hexanes) to provide **3e** as a colorless oil; yield: 85% (0.076 g; 0.3 mmol scale). ^1^H NMR (CDCl_3_, 400 MHz): δ = 7.47–7.43 (m, 2H), 7.35–7.32 (m, 2H), 4.29–4.11 (m, 4H), 1.34–1.30 (m, 6H) ppm. ^13^C­{^1^H} NMR (CDCl_3_, 100 MHz): δ = 136.1 (d, *J*_CP_ = 4 Hz, 2*C*), 135.9 (d, *J*_CP_ = 4 Hz), 129.6 (d, *J*_CP_ = 3 Hz, 2*C*), 126.9 (d, *J*_CP_ = 8 Hz), 64.5 (d, *J*_CP_ = 6 Hz), 15.9 (d, *J*_CP_ = 9 Hz) ppm.

#### 4.3.6 *S*-(4-Bromophenyl) *O*,*O*-Diethyl Phosphorodithioate (**3f**)^16b^

Following the general procedure, using *O*,*O*-diethyl phosphonothioate (**1a**; 0.3 mmol, 0.047 g), 4-bromobenzenethiol (**2f**, 0.3 mmol, 0.057 g), NaI (0.05 M, 0.023 g), and 3 mL of CH_3_CN then purified by column chromatography (SiO_2_, 10–15% ethyl acetate in hexanes) to provide **3f** as a colorless oil; yield: 71% (0.073 g; 0.3 mmol scale). ^1^H NMR (CDCl_3_, 400 MHz): δ = 7.49 (d, *J* = 8.4 Hz, 2H), 7.37 (dd, *J* = 8.8 and 2.0 Hz, 2H), 4.29–4.11 (m, 4H), 1.32 (t, *J* = 7.2 Hz, 6H) ppm. ^13^C­{^1^H} NMR (CDCl_3_, 100 MHz): δ = 136.3 (d, *J*_CP_ = 5 Hz, 2*C*), 132.6 (d, *J*_CP_ = 3 Hz, 2*C*), 127.6 (d, *J*_CP_ = 8 Hz), 124.2 (d, *J*_CP_ = 4 Hz), 64.6 (d, *J*_CP_ = 6 Hz, 2*C*), 15.9 (d, *J*_CP_ = 8 Hz, 2*C*) ppm.

#### 4.3.7 *O*,*O*-Diethyl *S*-(Thiophen-2-yl) Phosphorodithioate (**3g**)^15^

Following the general procedure, using *O*,*O*-diethyl phosphonothioate (**1a**; 0.3 mmol, 0.047 g), thiophene-2-thiol (**2g**, 0.3 mmol, 0.035 g), NaI (0.05 M, 0.023 g), and 3 mL of CH_3_CN then purified by column chromatography (SiO_2_, 10–15% ethyl acetate in hexanes) to provide **3g** as a colorless oil; yield: 51% (0.041 g; 0.3 mmol scale). ^1^H NMR (CDCl_3_, 400 MHz): δ = 7.46–7.44 (m, 1H), 7.19 (td, *J* = 3.6 and 1.2 Hz, 1H), 7.04 (dd, *J* = 5.2 and 3.6 Hz, 1H), 4.32–4.16 (m, 4H), 1.37–1.34 (m, 6H) ppm. ^13^C­{^1^H} NMR (CDCl_3_, 100 MHz): δ = 136.2 (d, *J*_CP_ = 6 Hz, 2*C*), 131.4 (d, *J*_CP_ = 5 Hz, 2*C*), 127.9 (d, *J*_CP_ = 4 Hz, 2*C*), 125.3 (d, *J*_CP_ = 9 Hz), 64.6 (d, *J*_CP_ = 5 Hz, 2*C*), 15.9 (d, *J*_CP_ = 9 Hz, 2*C*) ppm.

#### 4.3.8 *O*,*O*-Diethyl *S*-Hexyl Phosphorodithioate (**3h**)^16b^

Following the general procedure, using *O*,*O*-diethyl phosphonothioate (**1a**; 0.3 mmol, 0.047 g), hexane-1-thiol (**2h**, 0.3 mmol, 0.036 g), NaI (0.05 M, 0.023 g), and 3 mL of CH_3_CN then purified by column chromatography (SiO_2_, 10–15% ethyl acetate in hexanes) to provide **3h** as a colorless oil; yield: 58% (0.057 g; 0.3 mmol scale). ^1^H NMR (CDCl_3_, 400 MHz): δ = 4.23–4.05 (m, 4H), 2.86–2.79 (m, 2H), 1.67–1.60 (m, 2H), 1.40–1.23 (m, 13H), 0.88–0.84 (m, 3H) ppm. ^13^C­{^1^H} NMR (CDCl_3_, 100 MHz): δ = 63.8 (d, *J*_CP_ = 6 Hz, 2*C*), 33.6 (d, *J*_CP_ = 4 Hz), 31.3, 30.4 (d, *J*_CP_ = 6 Hz), 28.4, 22.5, 15.9 (d, *J*_CP_ = 8 Hz, 2*C*), 14.1 ppm.

#### 4.3.9 *S*-Benzyl *O*,*O*-Diisopropyl Phosphorodithioate (**3i**)^16b^

Following the general procedure, using *O,O*-diisopropyl phosphonothioate (**1b**; 0.3 mmol, 0.055 g), phenylmethanethiol (**2i**, 0.3 mmol, 0.038 g), NaI (0.05 M, 0.023 g), and 3 mL of CH_3_CN then purified by column chromatography (SiO_2_, 10–15% ethyl acetate in hexanes) to provide **3i** as a colorless oil; yield: 83% (0.076 g; 0.3 mmol scale). ^1^H NMR (CDCl_3_, 400 MHz): δ = 7.36–7.23 (m, 5H), 4.85–4.76 (m, 2H), 4.12 (d, *J* = 14.4 Hz, 2H), 1.33–1.28 (m, 12H) ppm. ^13^C­{^1^H} NMR (CDCl_3_, 100 MHz): δ = 137.2 (d, *J*_CP_ = 6 Hz), 129.2 (2*C*), 128.7 (2*C*), 127.6, 73.6 (d, *J*_CP_ = 7 Hz), 38.1 (d, *J*_CP_ = 4 Hz, 2*C*), 23.8 (d, *J*_CP_ = 5 Hz), 23.5 (d, *J*_CP_ = 5 Hz, 2*C*) ppm.

#### 4.3.10 *O*,*O*-Dibutyl *S*-(*p*-Tolyl) Phosphorodithioate (**3j**)^16b^

Following the general procedure, using *O,O*-dibutyl phosphonothioate (**1c**; 0.3 mmol, 0.063 g), 4-methylbenzenethiol (**2b**, 0.3 mmol, 0.038 g), NaI (0.05 M, 0.023 g), and 3 mL of CH_3_CN then purified by column chromatography (SiO_2_, 10–15% ethyl acetate in hexanes) to provide **3j** as a colorless oil; yield: 65% (0.065 g; 0.3 mmol scale). ^1^H NMR (CDCl_3_, 400 MHz): δ = 7.39 (dd, *J* = 8.4 and 2.4 Hz, 2H), 7.16 (d, *J* = 8.0 Hz, 2H), 4.20–4.04 (m, 4H), 2.35 (d, *J* = 2.0 Hz, 3H), 1.67–1.60 (m, 4H), 1.40–1.31 (m, 4H), 0.92–0.89 (m, 6H) ppm. ^13^C­{^1^H} NMR (CDCl_3_, 100 MHz): δ = 139.6 (d, *J*_CP_ = 3 Hz), 134.8 (d, *J*_CP_ = 4 Hz, 2*C*), 130.1 (d, *J*_CP_ = 3 Hz, 2*C*), 124.7 (d, *J*_CP_ = 7 Hz), 67.9 (d, *J*_CP_ = 6 Hz, 2*C*), 32.0 (d, *J*_CP_ = 8 Hz, 2*C*), 21.3 (d, *J*_CP_ = 10 Hz, 2*C*), 18.8 (d, *J*_CP_ = 8 Hz, 2*C*), 13.7 ppm.

#### 4.3.11 *O*,*O*-Dibutyl *S*-(4-Fluorophenyl) Phosphorodithioate (**3k**)

Following the general procedure, using *O,O*-dibutyl phosphonothioate (**1c**; 0.3 mmol, 0.063 g), 4-fluorobenzenethiol (**2d**, 0.3 mmol, 0.039 g), NaI (0.05 M, 0.023 g), and 3 mL of CH_3_CN then purified by column chromatography (SiO_2_, 10–15% ethyl acetate in hexanes) to provide **3k** as a colorless oil; yield: 67% (0.068 g; 0.3 mmol scale). ^1^H NMR (CDCl_3_, 400 MHz): δ = 7.51–7.47 (m, 2H), 7.07–7.03 (m, 2H), 4.19–4.03 (m, 4H), 1.67–1.59 (m, 4H), 1.39–1.30 (m, 4H), 0.92–0.89 (m, 6H) ppm. ^13^C­{^1^H} NMR (CDCl_3_, 100 MHz): δ = 163.8 (dd, *J*_CP_ = 250 and 3 Hz), 137.0 (dd, *J*_CP_ = 8 and 4 Hz, 2*C*), 123.7 (dd, *J*_CP_ = 9 and 3 Hz), 116.5 (dd, *J*_CP_ = 22 and 3 Hz, 2*C*), 68.2 (d, *J*_CP_ = 7 Hz, 2*C*), 32.0 (d, *J*_CP_ = 8 Hz, 2*C*), 18.9 (2*C*), 13.7 (2*C*) ppm. HRMS (EI) calcd for C_14_H_22_FO_2_PS_2_H^+^ [M + H]^+^ *m*/*z* 337.0856, found *m*/*z* 337.0850.

#### 4.3.12 *O*,*O*-Dibutyl *S*-(4-Chlorophenyl) Phosphorodithioate (**3l**)^16b^

Following the general procedure, using *O,O*-dibutyl phosphonothioate (**1c**; 0.3 mmol, 0.063 g), 4-chlorobenzenethiol (**2e**, 0.3 mmol, 0.044 g), NaI (0.05 M, 0.023 g), and 3 mL of CH_3_CN then purified by column chromatography (SiO_2_, 10–15% ethyl acetate in hexanes) to provide **3l** as a colorless oil; yield: 79% (0.084 g; 0.3 mmol scale). ^1^H NMR (CDCl_3_, 400 MHz): δ = 7.46–7.42 (m, 2H), 7.34–7.31 (m, 2H), 4.20–4.03 (m, 4H), 1.67–1.59 (m, 4H), 1.39–1.30 (m, 4H), 0.93–0.89 (m, 6H) ppm. ^13^C­{^1^H} NMR (CDCl_3_, 100 MHz): δ = 136.1 (d, *J*_CP_ = 5 Hz, 2*C*), 135.8 (d, *J*_CP_ = 4 Hz), 129.5 (d, *J*_CP_ = 3 Hz, 2*C*), 127.0 (d, *J*_CP_ = 8 Hz), 68.2 (d, *J*_CP_ = 7 Hz, 2*C*), 32.0 (d, *J*_CP_ = 8 Hz, 2*C*), 18.9 (2*C*), 13.72 (2*C*) ppm.

#### 4.3.13 *S*-(4-Bromophenyl) *O*,*O*-Dibutyl Phosphorodithioate (**3m**)^16b^

Following the general procedure, using *O,O*-dibutyl phosphonothioate (**1c**; 0.3 mmol, 0.063 g), 4-bromobenzenethiol (**2f**, 0.3 mmol, 0.057 g), NaI (0.05 M, 0.023 g), and 3 mL of CH_3_CN then purified by column chromatography (SiO_2_, 10–15% ethyl acetate in hexanes) to provide **3m** as a colorless oil; yield: 60% (0.072 g; 0.3 mmol scale). ^1^H NMR (CDCl_3_, 400 MHz): δ = 7.49–7.45 (m, 2H), 7.39–7.35 (m, 2H), 4.19–4.03 (m, 4H), 1.67–1.59 (m, 4H), 1.39–1.30 (m, 4H), 0.91 (t, *J* = 7.6 Hz, 6H) ppm. ^13^C­{^1^H} NMR (CDCl_3_, 100 MHz): δ = 136.3 (d, *J*_CP_ = 5 Hz, 2*C*), 132.5 (d, *J*_CP_ = 3 Hz, 2*C*), 127.6 (d, *J*_CP_ = 8 Hz), 124.0 (d, *J*_CP_ = 4 Hz), 68.2 (d, *J*_CP_ = 7 Hz, 2*C*), 32.0 (d, *J*_CP_ = 8 Hz, 2*C*), 18.9 (2*C*), 13.7 (2*C*) ppm.

#### 4.3.14 *O*,*O*-Diisobutyl *S*-(*p*-Tolyl) Phosphorodithioate (**3n**)

Following the general procedure, using *O*,*O*-diisobutyl phosphonothioate (**1d**; 0.3 mmol, 0.063 g), 4-methylbenzenethiol (**2b**, 0.3 mmol, 0.038 g), NaI (0.05 M, 0.023 g), and 3 mL of CH_3_CN then purified by column chromatography (SiO_2_, 10–15% ethyl acetate in hexanes) to provide **3n** as a colorless oil; yield: 73% (0.073 g; 0.3 mmol scale). ^1^H NMR (CDCl_3_, 400 MHz): δ = 7.41–7.38 (m, 2H), 7.16–7.13 (m, 2H), 3.96–3.80 (m, 4H), 2.34 (d, *J* = 2.4 Hz, 3H), 1.99–1.89 (m, 2H), 0.91 (d, *J* = 2.0 Hz, 6H), 0.89 (d, *J* = 2.0 Hz, 6H) ppm. ^13^C­{^1^H} NMR (CDCl_3_, 100 MHz): δ = 139.6 (d, *J*_CP_ = 3 Hz), 134.9 (d, *J*_CP_ = 5 Hz, 2*C*), 130.1 (d, *J*_CP_ = 3 Hz, 2*C*), 124.7 (d, *J*_CP_ = 8 Hz), 74.0 (d, *J*_CP_ = 7 Hz, 2*C*), 28.9 (d, *J*_CP_ = 8 Hz, 2*C*), 21.4, 18.93 (4*C*) ppm. HRMS (EI) calcd for C_15_H_25_O_2_PS_2_H^+^ [M + H]^+^ *m*/*z* 333.1106, found *m*/*z* 333.1102.

#### 4.3.15 *O*,*O*-Diethyl *Se*-phenyl Phosphoroselenothioate (**5a**)^15^

Following the general procedure, using *O*,*O*-diethyl phosphonothioate (**1a**; 0.3 mmol, 0.047 g), 1,2-diphenyldiselane (**4a**, 0.3 mmol, 0.094 g), *^n^* Bu_4_NI (0.07 M, 0.075 g), and 3 mL of CH_3_CN then purified by column chromatography (SiO_2_, 10–15% ethyl acetate in hexanes) to provide **5a** as a colorless oil; yield: 86% (0.080 g; 0.3 mmol scale). ^1^H NMR (CDCl_3_, 400 MHz): δ = 7.62–7.59 (m, 2H), 7.39–7.31 (m, 3H), 4.29–4.11 (m, 4H), 1.33–1.29 (m, 6H) ppm. ^13^C­{^1^H} NMR (CDCl_3_, 100 MHz): δ = 135.7 (d, *J*_CP_ = 4 Hz, 2*C*), 129.5 (d, *J*_CP_ = 3 Hz, 2*C*), 129.1 (d, *J*_CP_ = 3 Hz), 126.6 (d, *J*_CP_ = 8 Hz), 64.1 (d, *J*_CP_ = 6 Hz, 2*C*), 15.8 (d, *J*_CP_ = 9 Hz, 2*C*) ppm. ^31^P NMR (CDCl_3_, 162 MHz): δ = 81.61 ppm. HRMS (EI) calcd for C_10_H_15_O_2_PSSeH^+^ [M + H]^+^ *m*/*z* 310.9768, found *m*/*z* 310.9765.

#### 4.3.16 *O*,*O*-Diethyl *Se*-(*p*-tolyl) Phosphoroselenothioate (**5b**)^16a^

Following the general procedure, using *O*,*O*-diethyl phosphonothioate (**1a**; 0.3 mmol, 0.047 g), 1,2-di-*p*-tolyldiselane (**4b**, 0.3 mmol, 0.102 g), *^n^* Bu_4_NI (0.07 M, 0.075 g), and 3 mL of CH_3_CN then purified by column chromatography (SiO_2_, 10–15% ethyl acetate in hexanes) to provide **5b** as a colorless oil; yield: 72% (0.070 g; 0.3 mmol scale). ^1^H NMR (CDCl_3_, 400 MHz): δ = 7.49–7.46 (m, 2H), 7.14 (d, *J* = 7.6 Hz, 2H), 4.29–4.11 (m, 4H), 2.35 (d, *J* = 2.0 Hz, 3H), 1.33–1.29 (m, 6H) ppm. ^13^C­{^1^H} NMR (CDCl_3_, 100 MHz): δ = 139.4 (d, *J*_CP_ = 3 Hz), 135.8 (d, *J*_CP_ = 3 Hz, 2*C*), 130.4 (d, *J*_CP_ = 2 Hz, 2*C*), 122.9 (d, *J*_CP_ = 8 Hz), 64.1 (d, *J*_CP_ = 5 Hz, 2*C*), 21.4, 15.8 (d, *J*_CP_ = 9 Hz, 2*C*) ppm.

#### 4.3.17 *O*,*O*-Diethyl *Se*-(4-methoxyphenyl) Phosphoroselenothioate (**5c**)^15^

Following the general procedure, using *O*,*O*-diethyl phosphonothioate (**1a**; 0.3 mmol, 0.047 g), 1,2-bis­(4-methoxyphenyl)­diselane (**4c**, 0.3 mmol, 0.112 g), *^n^* Bu_4_NI (0.07 M, 0.075 g), and 3 mL of CH_3_CN then purified by column chromatography (SiO_2_, 10–15% ethyl acetate in hexanes) to provide **5c** as a colorless oil; yield: 80% (0.081 g; 0.3 mmol scale). ^1^H NMR (CDCl_3_, 400 MHz): δ = 7.50–7.46 (m, 2H), 6.86–6.82 (m, 2H), 4.25–4.08 (m, 4H), 3.77 (s, 3H), 1.31–1.28 (m, 6H) ppm. ^13^C­{^1^H} NMR (CDCl_3_, 100 MHz): δ = 160.4 (d, *J*_CP_ = 2 Hz), 137.4 (d, *J*_CP_ = 4 Hz, 2*C*), 116.6 (d, *J*_CP_ = 9 Hz), 115.1 (d, *J*_CP_ = 2 Hz, 2*C*), 63.9 (d, *J*_CP_ = 6 Hz, 2*C*), 55.3, 15.7 (d, *J*_CP_ = 8 Hz, 2*C*) ppm.

#### 4.3.18 *O*,*O*-Diethyl *Se*-(4-fluorophenyl) Phosphoroselenothioate (**5d**)^16a^

Following the general procedure, using *O*,*O*-diethyl phosphonothioate (**1a**; 0.3 mmol, 0.047 g), 1,2-bis­(4-fluorophenyl)­diselane (**4d**, 0.3 mmol, 0.104 g), *^n^* Bu_4_NI (0.07 M, 0.075 g), and 3 mL of CH_3_CN then purified by column chromatography (SiO_2_, 10–15% ethyl acetate in hexanes) to provide **5d** as a colorless oil; yield: 71% (0.070 g; 0.3 mmol scale). ^1^H NMR (CDCl_3_, 400 MHz): δ = 7.59–7.56 (m, 2H), 7.06–7.00 (m, 2H), 4.28–4.10 (m, 4H), 1.34–1.30 (m, 6H) ppm. ^13^C­{^1^H} NMR (CDCl_3_, 100 MHz): δ = 163.6 (dd, *J*_CF and CP_ = 248 and 3 Hz), 137.9 (dd, *J*_CF and CP_ = 8 and 4 Hz, 2*C*), 121.4 (dd, *J*_CF and CP_ = 9 and 3 Hz), 116.8 (dd, *J*_CF and CP_ = 22 and 3 Hz, 2*C*), 64.3 (d, *J*_CP_ = 6 Hz, 2*C*), 15.8 (d, *J*_CP_ = 8 Hz, 2*C*) ppm. ^19^F NMR (CDCl_3_, 376 MHz): δ = −111.45 ppm.

#### 4.3.19 *Se*-(4-Chlorophenyl) *O*,*O*-Diethyl Phosphoroselenothioate (**5e**)^16a^

Following the general procedure, using *O*,*O*-diethyl phosphonothioate (**1a**; 0.3 mmol, 0.047 g), 1,2-bis­(4-chlorophenyl)­diselane (**4e**, 0.3 mmol, 0.114 g), *^n^* Bu_4_NI (0.07 M, 0.075 g), and 3 mL of CH_3_CN then purified by column chromatography (SiO_2_, 10–15% ethyl acetate in hexanes) to provide **5e** as a colorless oil; yield: 69% (0.071 g; 0.3 mmol scale). ^1^H NMR (CDCl_3_, 400 MHz): δ = 7.55–7.51 (m, 2H), 7.32–7.29 (m, 2H), 4.29–4.10 (m, 4H), 1.34–1.30 (m, 6H) ppm. ^13^C­{^1^H} NMR (CDCl_3_, 100 MHz): δ = 137.1 (d, *J*_CP_ = 4 Hz, 2*C*), 135.7 (d, *J*_CP_ = 3 Hz), 129.8 (d, *J*_CP_ = 2 Hz, 2*C*), 124.8 (d, *J*_CP_ = 9 Hz), 64.3 (d, *J*_CP_ = 6 Hz, 2*C*), 15.8 (d, *J*_CP_ = 8 Hz, 2*C*) ppm.

#### 4.3.20 *O*,*O*-Diethyl *Se*-(naphthalen-1-yl) Phosphoroselenothioate (**5f**)^16a^

Following the general procedure, using *O*,*O*-diethyl phosphonothioate (**1a**; 0.3 mmol, 0.047 g), 1,2-di­(naphthalen-1-yl)­diselane (**4f**, 0.3 mmol, 0.124 g), *^n^* Bu_4_NI (0.07 M, 0.075 g), and 3 mL of CH_3_CN then purified by column chromatography (SiO_2_, 10–15% ethyl acetate in hexanes) to provide **5f** as a colorless oil; yield: 71% (0.077 g; 0.3 mmol scale). ^1^H NMR (CDCl_3_, 400 MHz): δ = 8.47 (d, *J* = 8.4 Hz, 1H), 7.95–7.90 (m, 2H), 7.85 (d, *J* = 8.4 Hz, 1H), 7.60–7.50 (m, 2H), 7.45–7.41 (m, 1H), 4.24–4.05 (m, 4H), 1.20–1.17 (m, 6H) ppm. ^13^C­{^1^H} NMR (CDCl_3_, 100 MHz): δ = 136.7 (d, *J*_CP_ = 4 Hz), 134.9 (d, *J*_CP_ = 3 Hz), 134.4 (d, *J*_CP_ = 2 Hz), 130.6 (d, *J*_CP_ = 4 Hz), 128.7, 128.3, 127.1, 126.6, 126.2 (d, *J*_CP_ = 9 Hz), 126.0 (d, *J*_CP_ = 4 Hz), 64.3 (d, *J*_CP_ = 5 Hz), 15.7 (d, *J*_CP_ = 9 Hz) ppm.

#### 4.3.21 *O*,*O*-Diethyl *Se*-(thiophen-2-yl) Phosphoroselenothioate (**5g**)

Following the general procedure, using *O*,*O*-diethyl phosphonothioate (**1a**; 0.3 mmol, 0.047 g), 1,2-di­(thiophen-2-yl)­diselane (**4g**, 0.3 mmol, 0.097 g), *^n^* Bu_4_NI (0.07 M, 0.075 g), and 3 mL of CH_3_CN then purified by column chromatography (SiO_2_, 10–15% ethyl acetate in hexanes) to provide **5g** as a colorless oil; yield: 77% (0.073 g; 0.3 mmol scale). ^1^H NMR (CDCl_3_, 400 MHz): δ = 7.48–7.47 (m, 1H), 7.24–7.22 (m, 1H), 7.05–7.02 (m, 1H), 4.31–4.15 (m, 4H), 1.38–1.34 (m, 6H) ppm. ^13^C­{^1^H} NMR (CDCl_3_, 100 MHz): δ = 137.1 (d, *J*_CP_ = 5 Hz), 132.2 (d, *J*_CP_ = 4 Hz), 128.4 (d, *J*_CP_ = 3 Hz), 119.9 (d, *J*_CP_ = 10 Hz), 64.4 (d, *J*_CP_ = 5 Hz, 2*C*), 15.8 (d, *J*_CP_ = 8 Hz, 2*C*) ppm. HRMS (EI) calcd for C_8_H_13_O_2_PS_2_SeH^+^ [M + H]^+^ *m*/*z* 316.9333, found *m*/*z* 316.9329.

#### 4.3.22 *O*,*O*-Diisopropyl *Se*-phenyl Phosphoroselenothioate (**5h**)^16a^

Following the general procedure, using *O,O*-diisopropyl phosphonothioate (**1b**; 0.3 mmol, 0.055 g), 1,2-diphenyldiselane (**4a**, 0.3 mmol, 0.094 g), *^n^* Bu_4_NI (0.07 M, 0.075 g), and 3 mL of CH_3_CN then purified by column chromatography (SiO_2_, 10–15% ethyl acetate in hexanes) to provide **5h** as a colorless oil; yield: 90% (0.091 g; 0.3 mmol scale). ^1^H NMR (CDCl_3_, 400 MHz): δ = 7.66–7.63 (m, 2H), 7.37–7.29 (m, 3H), 4.94–4.81 (m, 2H), 1.33 (d, *J* = 6.4 Hz, 6H), 1.27 (d, *J* = 6.4 Hz, 6H) ppm. ^13^C­{^1^H} NMR (CDCl_3_, 100 MHz): δ = 135.4 (d, *J*_CP_ = 4 Hz, 2*C*), 129.4 (d, *J*_CP_ = 2 Hz, 2*C*), 128.8 (d, *J*_CP_ = 3 Hz), 127.5 (d, *J*_CP_ = 8 Hz), 73.9 (d, *J*_CP_ = 7 Hz, 2*C*), 23.9 (d, *J*_CP_ = 4 Hz, 2*C*), 23.4 (d, *J*_CP_ = 5 Hz, 2*C*) ppm.

#### 4.3.23 *O*,*O*-Dibutyl *Se*-phenyl Phosphoroselenothioate (**5i**)^15^

Following the general procedure, using *O,O*-dibutyl phosphonothioate (**1c**; 0.3 mmol, 0.063 g), 1,2-diphenyldiselane (**4a**, 0.3 mmol, 0.094 g), *^n^* Bu_4_NI (0.07 M, 0.075 g), and 3 mL of CH_3_CN then purified by column chromatography (SiO_2_, 10–15% ethyl acetate in hexanes) to provide **5i** as a colorless oil; yield: 71% (0.078 g; 0.3 mmol scale). ^1^H NMR (CDCl_3_, 400 MHz): δ = 7.62–7.59 (m, 2H), 7.39–7.30 (m, 3H), 4.21–4.03 (m, 4H), 1.67–1.60 (m, 4H), 1.39–1.30 (m, 4H), 0.92–0.88 (m, 6H) ppm. ^13^C­{^1^H} NMR (CDCl_3_, 100 MHz): δ = 135.7 (d, *J*_CP_ = 4 Hz, 2*C*), 129.5 (d, *J*_CP_ = 3 Hz, 2*C*), 129.0 (d, *J*_CP_ = 2 Hz), 126.7 (d, *J*_CP_ = 9 Hz), 67.9 (d, *J*_CP_ = 6 Hz, 2*C*), 31.9 (d, *J*_CP_ = 8 Hz, 2*C*), 18.9 (2*C*), 13.7 (2*C*) ppm.

#### 4.3.24 *O*,*O*-Dibutyl *Se*-(*p*-tolyl) Phosphoroselenothioate (**5j**)

Following the general procedure, using *O,O*-dibutyl phosphonothioate (**1c**; 0.3 mmol, 0.063 g), 1,2-di-p-tolyldiselane (**4b**, 0.3 mmol, 0.102 g), *^n^* Bu_4_NI (0.07 M, 0.075 g), and 3 mL of CH_3_CN then purified by column chromatography (SiO_2_, 10–15% ethyl acetate in hexanes) to provide **5j** as a colorless oil; yield: 70% (0.080 g; 0.3 mmol scale). ^1^H NMR (CDCl_3_, 400 MHz): δ = 7.49–7.46 (m, 2H), 7.13 (d, *J* = 8.0 Hz, 2H), 4.20–4.03 (m, 4H), 2.35 (d, *J* = 2.0 Hz, 3H), 1.67–1.60 (m, 4H), 1.41–1.29 (m, 4H), 0.92–0.89 (m, 6H) ppm. ^13^C­{^1^H} NMR (CDCl_3_, 100 MHz): δ = 139.3 (d, *J*_CP_ = 3 Hz), 135.8 (d, *J*_CP_ = 4 Hz, 2*C*), 130.3 (d, *J*_CP_ = 3 Hz, 2*C*), 123.0 (d, *J*_CP_ = 9 Hz), 67.8 (d, *J*_CP_ = 7 Hz, 2*C*), 31.9 (d, *J*_CP_ = 9 Hz, 2*C*), 21.4, 18.9 (2*C*), 13.8 (2*C*) ppm. HRMS (EI) calcd for C_15_H_25_O_2_PSSeH^+^ [M + H]^+^ *m*/*z* 381.0551, found *m*/*z* 381.0547.

#### 4.3.25 *O*,*O*-Dibutyl *Se*-(4-fluorophenyl) Phosphoroselenothioate (**5k**)

Following the general procedure, using *O,O*-dibutyl phosphonothioate (**1c**; 0.3 mmol, 0.063 g), 1,2-bis­(4-fluorophenyl)­diselane (**4d**, 0.3 mmol, 0.104 g), *^n^* Bu_4_NI (0.07 M, 0.075 g), and 3 mL of CH_3_CN then purified by column chromatography (SiO_2_, 10–15% ethyl acetate in hexanes) to provide **5k** as a colorless oil; yield: 69% (0.081 g; 0.3 mmol scale). ^1^H NMR (CDCl_3_, 400 MHz): δ = 7.59–7.55 (m, 2H), 7.04–6.99 (m, 2H), 4.19–4.02 (m, 4H), 1.67–1.60 (m, 4H), 1.41–1.29 (m, 4H), 0.92–0.89 (m, 6H) ppm. ^13^C­{^1^H} NMR (CDCl_3_, 100 MHz): δ = 163.5 (dd, *J*_CF and CP_ = 248 and 3 Hz), 137.9 (dd, *J*_CF and CP_ = 9 and 4 Hz, 2*C*), 121.4 (dd, *J*_CF and CP_ = 9 and 3 Hz), 116.7 (dd, *J*_CF and CP_ = 22 and 3 Hz, 2*C*), 68.0, 67.98 (d, *J*_CP_ = 7 Hz, 2*C*), 32.0, 31.9 (d, *J*_CP_ = 8 Hz, 2*C*), 18.9 (2*C*), 13.7 (2*C*) ppm. ^19^F NMR (CDCl_3_, 376 MHz): δ = −111.60 ppm. HRMS (EI) calcd for C_14_H_22_FO_2_PSSeH^+^ [M + H]^+^ *m*/*z* 385.0300, found *m*/*z* 385.0296.

#### 4.3.26 *O*,*O*-Dibutyl *Se*-(4-chlorophenyl) Phosphoroselenothioate (**5l**)^16a^

Following the general procedure, using *O,O*-dibutyl phosphonothioate (**1c**; 0.3 mmol, 0.063 g), 1,2-bis­(4-chlorophenyl)­diselane (**4e**, 0.3 mmol, 0.114 g), *^n^* Bu_4_NI (0.07 M, 0.075 g), and 3 mL of CH_3_CN then purified by column chromatography (SiO_2_, 10–15% ethyl acetate in hexanes) to provide **5l** as a colorless oil; yield: 68% (0.082 g; 0.3 mmol scale). ^1^H NMR (CDCl_3_, 400 MHz): δ = 7.55–7.51 (m, 2H), 7.31–7.28 (m, 2H), 4.20–4.02 (m, 4H), 1.67–1.60 (m, 4H), 1.40–1.30 (m, 4H), 0.93–0.89 (m, 6H) ppm. ^13^C­{^1^H} NMR (CDCl_3_, 100 MHz): δ = 137.0 (d, *J*_CP_ = 4 Hz, 2*C*), 135.6 (d, *J*_CP_ = 4 Hz), 129.7 (d, *J*_CP_ = 2 Hz, 2*C*), 124.9 (d, *J*_CP_ = 9 Hz), 68.1 (d, *J*_CP_ = 6 Hz, 2*C*), 31.9 (d, *J*_CP_ = 9 Hz, 2*C*), 18.9 (2*C*), 13.7 (2*C*) ppm.

#### 4.3.27 *O*,*O*-Diisobutyl *Se*-phenyl Phosphoroselenothioate (**5m**)^15^

Following the general procedure, using *O*,*O*-diisobutyl phosphonothioate (**1d**; 0.3 mmol, 0.063 g), 1,2-diphenyldiselane (**4a**, 0.3 mmol, 0.094 g), *^n^* Bu_4_NI (0.07 M, 0.075 g), and 3 mL of CH_3_CN then purified by column chromatography (SiO_2_, 10–15% ethyl acetate in hexanes) to provide **5m** as a colorless oil; yield: 79% (0.087 g; 0.3 mmol scale). ^1^H NMR (CDCl_3_, 400 MHz): δ = 7.63–7.60 (m, 2H), 7.38–7.29 (m, 3H), 3.97–3.79 (m, 4H), 1.98–1.88 (m, 2H), 0.91–0.88 (m, 12H) ppm. ^13^C­{^1^H} NMR (CDCl_3_, 100 MHz): δ = 135.8 (d, *J*_CP_ = 4 Hz, 2*C*), 129.4 (d, *J*_CP_ = 2 Hz, 2*C*), 129.0 (d, *J*_CP_ = 3 Hz), 126.6 (d, *J*_CP_ = 8 Hz), 73.9 (d, *J*_CP_ = 7 Hz, 2*C*), 28.9 (d, *J*_CP_ = 9 Hz, 2*C*), 18.9 (4*C*) ppm.

#### 4.3.28 *O*,*O*-Diisopropyl *Te*-phenyl Phosphorotellurothioate (**7a**)^15^

Following the general procedure, using *O,O*-diisopropyl phosphonothioate (**1b**; 0.3 mmol, 0.055 g), diphenyl ditelluride (**6**, 0.3 mmol, 0.123 g), *^n^* Bu_4_NI (0.07 M, 0.075 g), and 3 mL of CH_3_CN then purified by column chromatography (SiO_2_, 10–15% ethyl acetate in hexanes) to provide **7a** as a colorless oil; yield: 62% (0.072 g; 0.3 mmol scale). ^1^H NMR (CDCl_3_, 400 MHz): δ = 7.87–7.84 (m, 2H), 7.38–7.37 (m, 1H), 7.29–7.25 (m, 2H), 4.93–4.80 (m, 2H), 1.34 (d, *J* = 6.0 Hz, 6H), 1.28 (d, *J* = 6.0 Hz, 6H) ppm. ^13^C­{^1^H} NMR (CDCl_3_, 100 MHz): δ = 139.6 (d, *J*_CP_ = 4 Hz, 2*C*), 129.7 (d, *J*_CP_ = 2 Hz, 2*C*), 128.9 (d, *J*_CP_ = 2 Hz), 114.5 (d, *J*_CP_ = 7 Hz), 114.44, 73.4 (d, *J*_CP_ = 7 Hz, 2*C*), 24.0 (d, *J*_CP_ = 4 Hz, 2*C*), 23.4 (d, *J*_CP_ = 5 Hz, 2*C*) ppm. ^31^P NMR (CDCl_3_, 162 MHz): δ = 50.24 ppm.

#### 4.3.29 *O*,*O*-Dibutyl *Te*-phenyl Phosphorotellurothioate (**7b**)^15^

Following the general procedure, using *O,O*-dibutyl phosphonothioate (**1c**; 0.3 mmol, 0.063 g), diphenyl ditelluride (**6**, 0.3 mmol, 0.123 g), *^n^* Bu_4_NI (0.07 M, 0.075 g), and 3 mL of CH_3_CN then purified by column chromatography (SiO_2_, 10–15% ethyl acetate in hexanes) to provide **7b** as a colorless oil; yield: 76% (0.095 g; 0.3 mmol scale). ^1^H NMR (CDCl_3_, 400 MHz): δ = 7.84–7.82 (m, 2H), 7.39–7.35 (m, 1H), 7.29–7.25 (m, 2H), 4.16–3.99 (m, 4H), 1.68–1.61 (m, 4H), 1.39–1.30 (m, 4H), 0.92–0.88 (m, 6H) ppm. ^13^C­{^1^H} NMR (CDCl_3_, 100 MHz): δ = 139.9 (d, *J*_CP_ = 3 Hz, 2*C*), 129.8 (d, *J*_CP_ = 2 Hz, 2*C*), 129.1 (d, *J*_CP_ = 2 Hz), 113.7 (d, *J*_CP_ = 7 Hz), 67.6 (d, *J*_CP_ = 6 Hz, 2*C*), 31.8 (d, *J*_CP_ = 9 Hz, 2*C*), 19.00, 13.76 ppm.

## Supplementary Material



## Data Availability

The data underlying this study are available in the published article and its Supporting Information.

## Notes

Experimental procedures, experimental setup pictures, and scanned copies of ^1^H NMR, ^13^C NMR for all compounds (**3a**–**3n, 5a**–**5m** and **7a**–**7b**; a total of 29 derivatives), ^19^F NMR (for **3d**, **3k**, **5d**, and **5k**), ^31^P NMR spectra (for **3a** and **5a**), HRMS (**3a**, **3k**, **3n**, **5a**, **5g**, **5j**, and **5k**), and cyclic voltammograms (PDF)
