# Supplementary material for: Electrochemical Phosphorochalcogenations for the Synthesis of Phosphorochalcogenothioates
Source: J Org Chem. 2026 Mar 25;91(14):4962–75. doi: 10.1021/acs.joc.5c03080 (PMC13077701; doi:10.1021/acs.joc.5c03080)
Supplement: Supplementary file 1 [file jo5c03080_si_001.pdf]

Supporting Information

Electrochemical Phosphorochalcogenations for the Synthesis of Phosphorochalcogenothioates

Indrajit Karmakar,[a] Xiang-Wei Huang,[a] Yu-Hao Chen,[a] Chieh-An Cheng,[a] Pei-Chi Kuo,[a] Chin-Fa Lee*[a,b,c]

[a]*Department of Chemistry, National Chung Hsing University, Taichung City 40227, Taiwan (R.O.C.)*
[b]*i-Center for Advanced Science and Technology (iCAST), National Chung Hsing University, Taichung City 40227, Taiwan (R.O.C.)*
[c]*Innovation and Development Center of Sustainable Agriculture (IDCSA), National Chung Hsing University, Taichung City 40227, Taiwan (R.O.C.)*

*Corresponding author. E-mail: cfalee@dragon.nchu.edu.tw

ORCID: http://orcid.org/0000-0003-0735-5691

Table of Contents

1. General……………………..……………………..………………………..…..……………..…....…S1

2. Pictorial views of the experimental Setup (**Figure 1**)……………………..………………………………....S1

3. General Procedure for the Synthesis of Compounds **3/5/7**……………………………………………....S2

4. Large-scale synthesis of compounds **3a** and **5a**……………………………………….………...…S2

5. General procedure for cyclic voltammetry……………………………………………………………. **S2-S3**

6. Cyclic voltammograms of **1a, 2a, 4a, NaI, ^{n}Bu$_4$NI** and other mixtures (**Figure 2**)………………...……….**S3**

7. Scanned copies of ^{1}H NMR, ^{13}C NMR, ^{31}P NMR (for **3a** and **5a**), ^{19}F NMR (for **3d**, **3k**, **5d**, and **5k**) spectra for all the synthesized dibenzoselenophenes **3/5/7** (**3a-3n**, **5a-5m**, **7a-7b**) and HRMS spectra of **3a**, **3k**, **3n**, **5a**, **5g**, **5j**, **5k**, **adducts 9** and **adduct 10** (**Figure S3 – S75**) ……………….………............................**S4-S76**

1. General. All solvents used in this study were distilled and dried prior to use, following standard procedures. ^{1}H, ^{13}C, ^{19}F, and ^{31}P NMR spectra were recorded on a Varian Unity Inova-600 or a Varian Mercury-400 NMR spectrometer using $CDCl_3$ as the solvent. Chemical shifts (δ) are reported in ppm relative to the internal standard, TMS. Signal multiplicities are denoted as s (singlet), d (doublet), t (triplet), and m (multiplet), and coupling constants (*J*) are reported in Hz. Mass spectrometry analyses were carried out on a Jeol JMS-HX 110 spectrometer. Liquid chromatography (LC) analyses were performed using a SHIMADZU LC-2050C 3D system, while LC-mass spectrometry (LC-MS) data were collected on a DPiMS-2020. Liquid chromatography-tandem mass spectrometry (LC-MS/MS) was performed using an LC-8045 system. Protein analyses were conducted on a Waters SYNAPT HDMS Q-TOF mass spectrometer (ESI-Q-TOF). Cyclic voltammetry measurements were performed on a CHI Instruments 750A potentiostat using acetonitrile as the solvent. Melting points were determined using a Büchi 535 melting point apparatus and are reported as uncorrected. Thin-layer chromatography (TLC) was carried out on silica gel 60 F_{254} plates (Merck). Electrochemical reactions were conducted in an undivided cell equipped with platinum electrodes (IKA), with dimensions of 0.7 × 0.7 × 0.2 cm for small-scale reactions and 2.8 × 0.7 × 0.2 cm for larger-scale reactions. A GW Instek GPS-2303 laboratory DC power supply (350 W, 450 VA, 50/60 Hz) was used as the power source.

2. Pictorial views of the experimental Setup

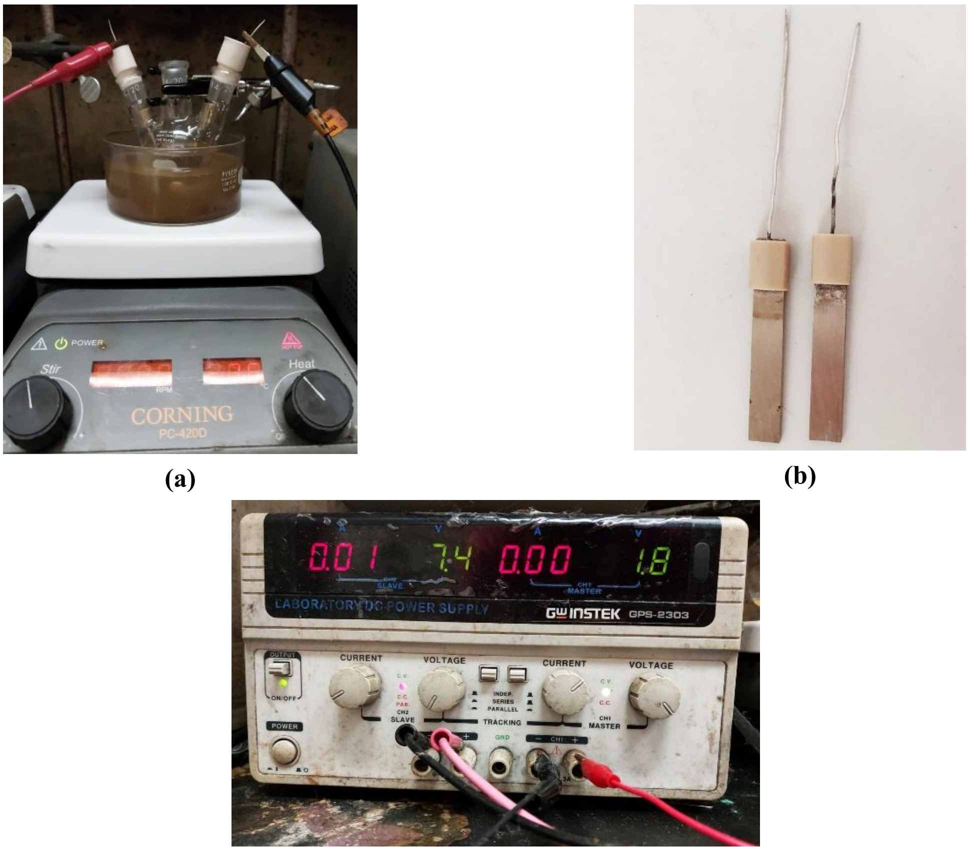

(a) (b)

(C)

Figure S1: (a) Larger-scale experimental setup; (b) IKA made Platinum/Platinum plate electrodes; (c) GPS-2303 laboratory DC power supply (350 W, 450 VA, 50/60 Hz)

3. General Procedure for the Synthesis of Compounds 3/5/7

An oven-dried 10 mL glass vessel was sequentially charged with the substituted *O*,*O*-dialkyl phosphonothioates (**1**, 0.3 mmol), the corresponding thiols or diaryl diselenides or diphenyl ditelluride (**2**/**4**/**6**, 0.3 mmol), and 3 mL of a 0.05/0.07 M NaI/$^{n}Bu_4NI$ electrolyte solution in acetonitrile (CH_3CN), along with a magnetic stir bar. Platinum plates (working dimensions: 0.7 × 0.7 × 0.2 cm) were used as both the anode and cathode, positioned 0.5 cm apart to form an undivided electrochemical cell. A constant direct current of 10 mA was applied to the stirred reaction mixture at room temperature for 3 h. Reaction progress was monitored periodically by thin-layer chromatography (TLC). Upon completion, 20 mL of a 3:1 (*v*/*v*) ethyl acetate/water mixture was added, and the mixture was transferred to a separatory funnel and shaken vigorously. The organic layer was separated, dried over anhydrous sodium sulfate, and concentrated under reduced pressure. The crude product was purified by column chromatography using EtOAc-hexane mixtures as eluents to afford the desired products **3**/**5**/**7** (**3a**-**3n**, **5a**-**5m** and **7a**-**7b**). In total, 29 derivatives were synthesized and fully characterized by detailed spectroscopic analysis, including ^{1}H NMR, ^{13}C NMR, ^{31}P NMR (for **3a**, **5a**, and **7a**), ^{19}F NMR (for **3d**, **3k**, **5d**, and **5k**), and HRMS (for unknown compounds).

4. Larger-scale synthesis of Compounds 3a and 5a

An oven-dried 50 mL glass vessel was sequentially charged with the substituted *O*,*O*-diethyl phosphonothioate (**1a**, 3.0 mmol and 5.0 mmol, 0.462 g and 0.770 g), the corresponding benzenethiol (**2a**, 3.0 mmol and 5.0 mmol, 0.330 g and 0.550 g)/diphenyldiselane (**4a**, 3.0 mmol and 5.0 mmol, 0.936 g and 1.560 g), and 8 mL of a 0.19/0.25 M NaI (0.230 g)/$^{n}Bu_4NI$ (0.750 g) electrolyte solution in acetonitrile (CH_3CN), along with a magnetic stir bar. Platinum plates (working dimensions: 2.8 × 0.7 × 0.2 cm) were used as both the anode and cathode, positioned 0.5 cm apart to form an undivided electrochemical cell. A constant direct current of 10 mA was applied to the stirred reaction mixture at room temperature for 5 h. Reaction progress was monitored periodically by thin-layer chromatography (TLC). Upon completion, 60 mL of a 3:1 (*v*/*v*) ethyl acetate/water mixture was added, and the mixture was transferred to a separatory funnel and shaken vigorously. The organic layer was separated, dried over anhydrous sodium sulfate, and concentrated under reduced pressure. The crude product was purified by column chromatography using EtOAc-hexane mixtures as eluents to afford the desired products *O*,*O*-diethyl *S*-phenyl phosphorodithioate (**3a**) and *O*,*O*-diethyl *Se*-phenyl phosphoroselenothioate (**5a**) with 76% and 75% (0.601 g and 0.989 g) and 79% and 78% (0.736 g and 1.206 g) yields.

5. General procedure for cyclic voltammetry

Electrochemical tests were performed on a CHI Instruments 750A potentiostat using acetonitrile as solvent. A standard cyclic voltammetric (CV) experiment based on a three-electrode system is conducted. The working electrode utilizes BAS glassy carbon (3 mm diameter), while the reference and auxiliary electrodes employ Ag/AgCl (saturated) and a platinum wire, respectively. Potentials are reported vs. Ag/AgCl (saturated). The working electrode was polished with 0.03 μm aluminium on felt pads (Buehler) before each experiment. The reference is Ag/Ag^{+} electrode, and 10 mL of electrolyte solution containing 300 mg $^{n}Bu_4NBF_4$ in CH_3CN was poured into the electrochemical cell in all experiments. 3 mg of each of the samples, such as **1a**, **2a**, **4a**, **NaI** and **$^{n}Bu_4NI$** and other mixtures, was used for the purpose.

CV plotting convention: IUPAC

Working electrode: BAS glassy carbon (3 mm diameter)

Counter electrode: Platinum wire

Reference electrodes: Ag/Ag^+ electrode

Temperature: Room temperature

Starting point: 0.0

Direction of scan: + Direction

Potential scan ranged: From 0 to +2.0 V

Scan rate: 0.1 V/s

Solvent: CH_3CN (10 mL)

Electrolyte: ^{n}Bu_4NBF_4 (0.09 M) in 10 mL CH_3CN

NaI: 0.002 M in 10 mL CH_3CN

^{n}Bu_4NI: 0.001 M in 10 mL CH_3CN

1a: 0.002 M in 10 mL CH_3CN

2a: 0.003 M in 10 mL CH_3CN

4a: 0.001 M in 10 mL CH_3CN

6. Cyclic voltammograms of 1a, 2a, 4a, NaI, ^{n}Bu_4NI and other mixtures:

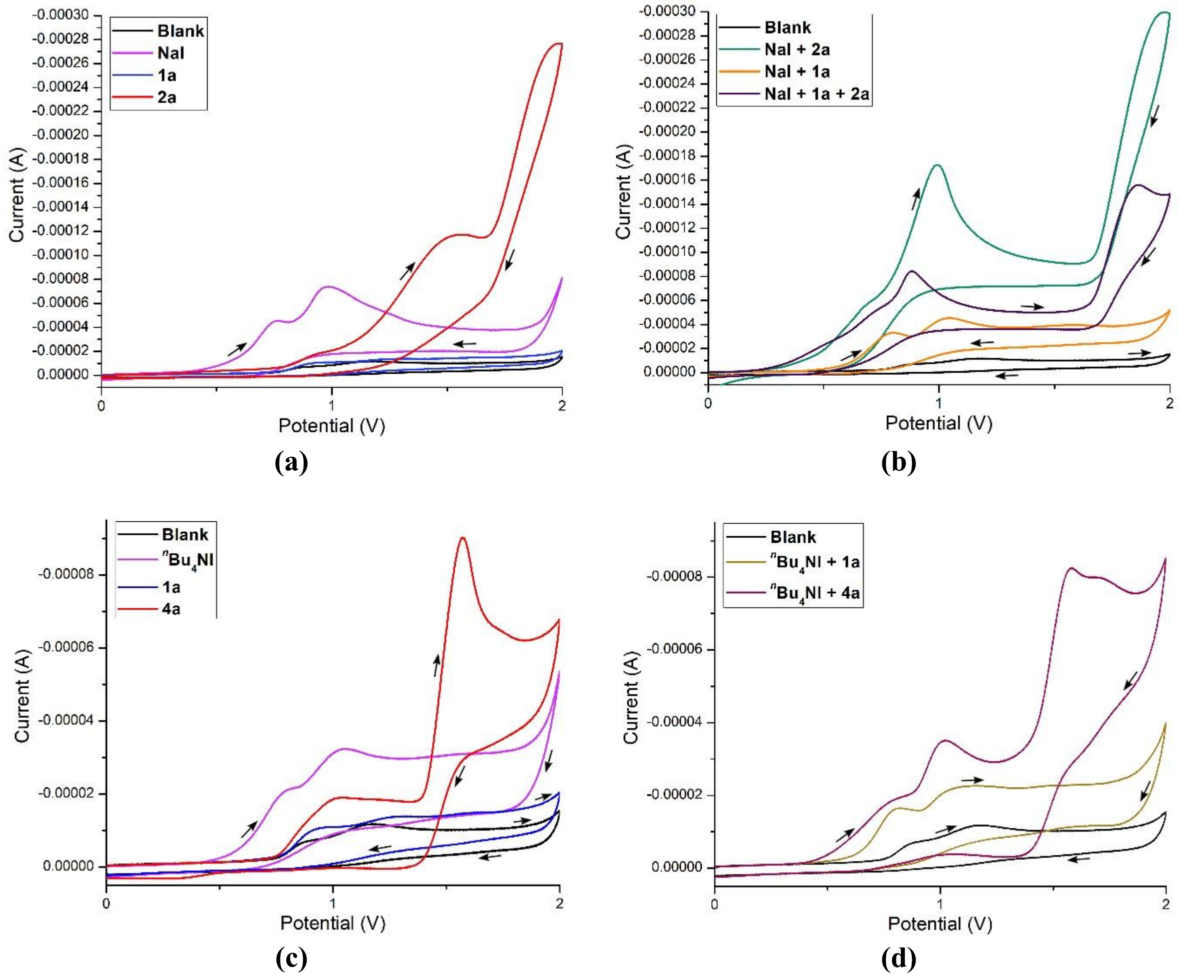

Figure S2. Cyclic voltammetry

7. Scanned copies of ^{1}H NMR, ^{13}C NMR, ^{31}P NMR (for 3a, 5a, and 7a), ^{19}F NMR (for 3d, 3k, 5d, and 5k) spectra for all the synthesized dibenzoselenophenes 3/5/7 (3a-3n, 5a-5m, and 7a-7b) and HRMS spectra of 3a, 3k, 3n, 5a, 5g, 5j, 5k, adducts 9 and adduct 10 (Figure S3 – S75)

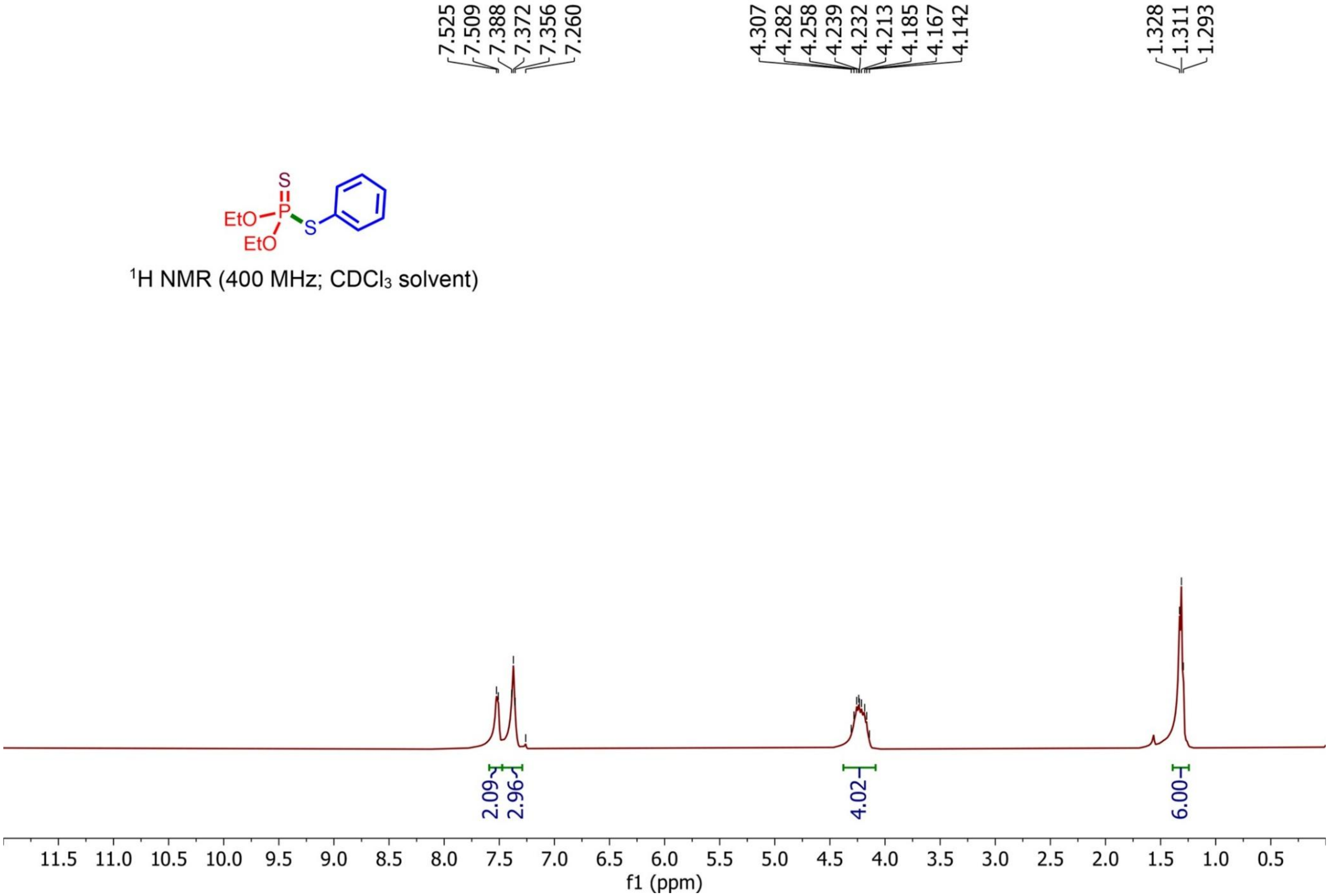

Figure S3. ^{1}H NMR spectrum of *O*,*O*-diethyl *S*-phenyl phosphorodithioate (**3a**)

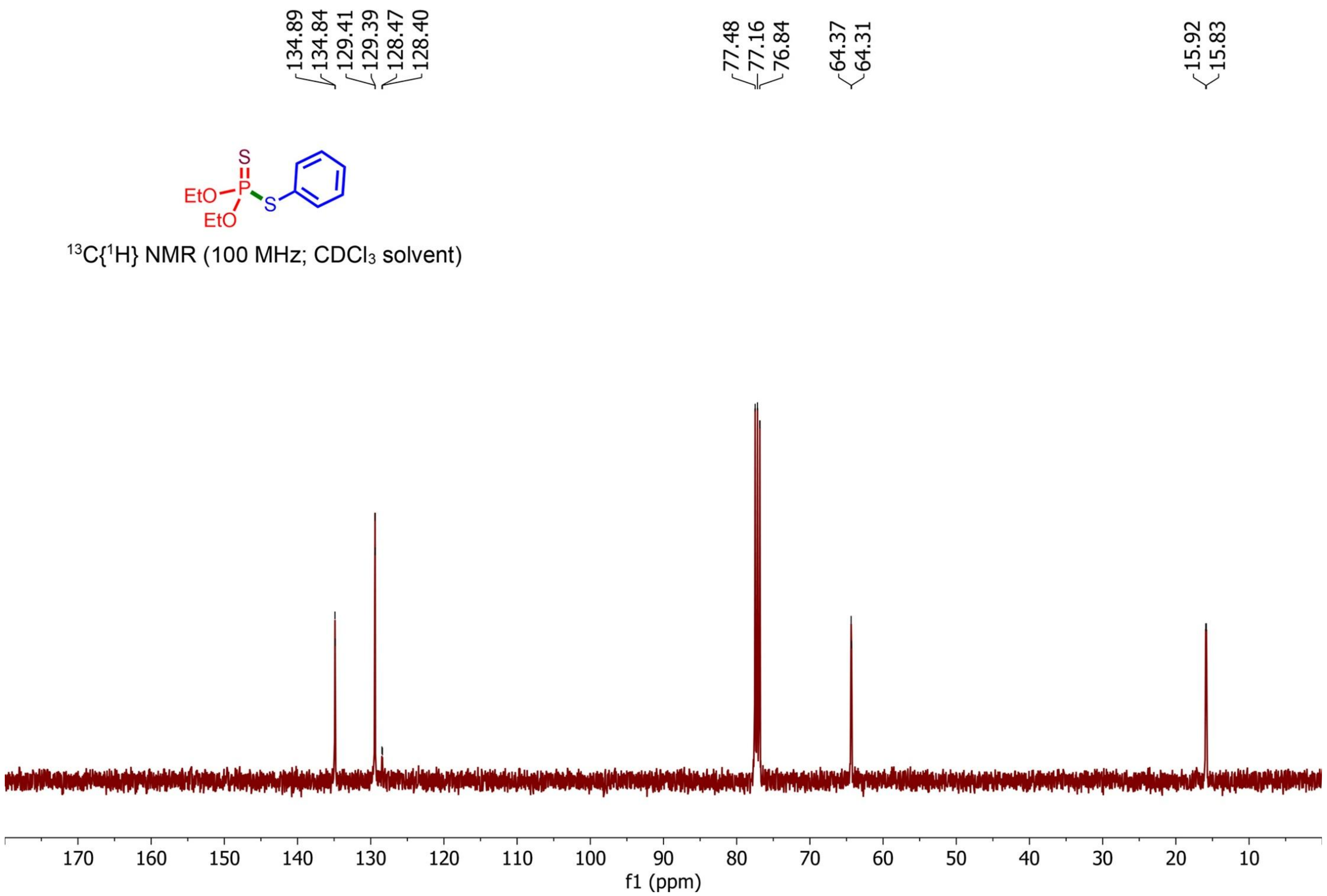

Figure S4. $^{13}C\{^1H\}$ NMR spectrum of *O*,*O*-diethyl *S*-phenyl phosphorodithioate (**3a**)

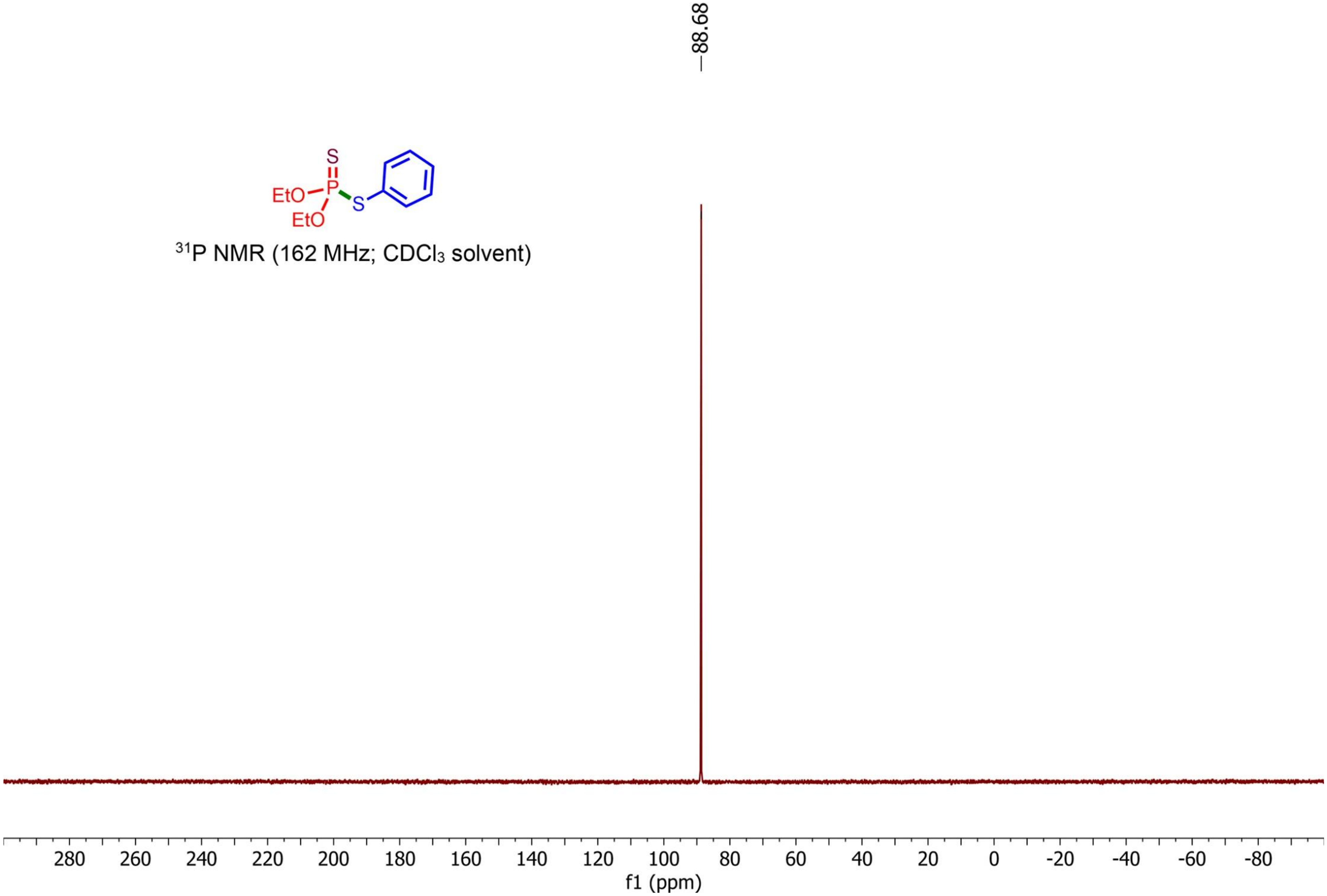

Figure S5. ^{31}P NMR spectrum of *O*,*O*-diethyl *S*-phenyl phosphorodithioate (**3a**)

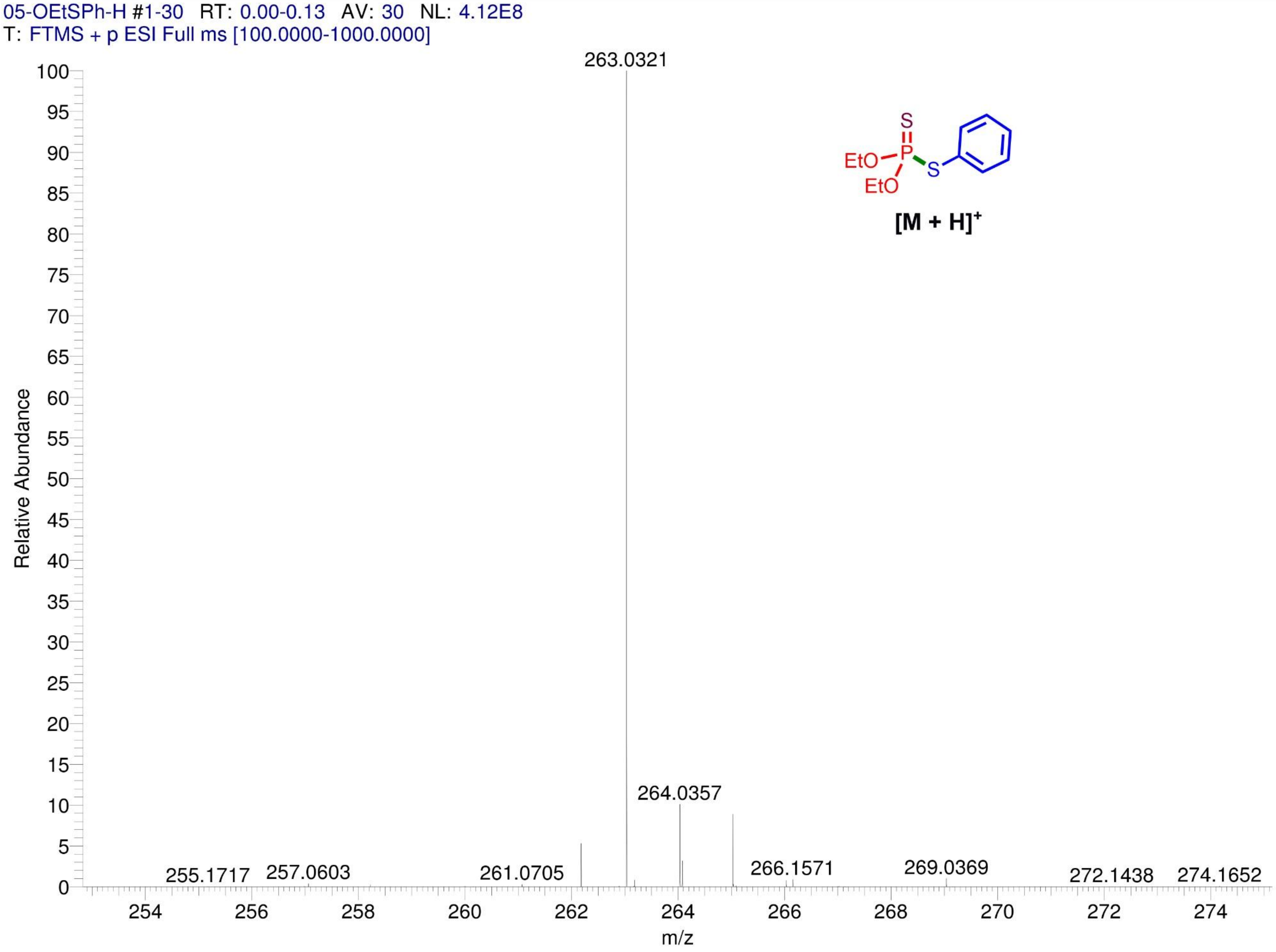

Figure S6. HRMS spectrum of *O*,*O*-diethyl *S*-phenyl phosphorodithioate (**3a**)

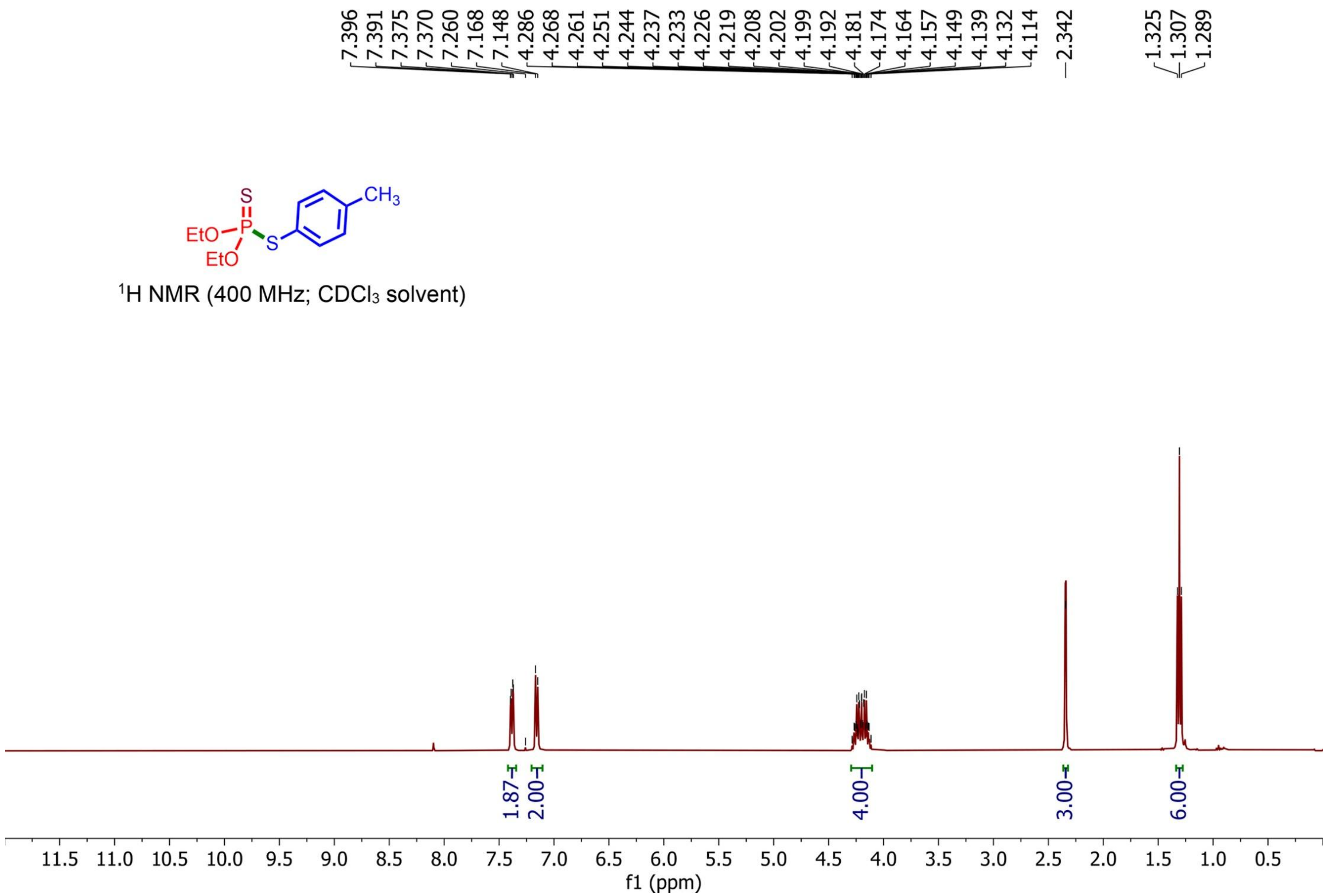

Figure S7. ^{1}H NMR spectrum of *O*,*O*-diethyl *S*-(*p*-tolyl) phosphorodithioate (**3b**)

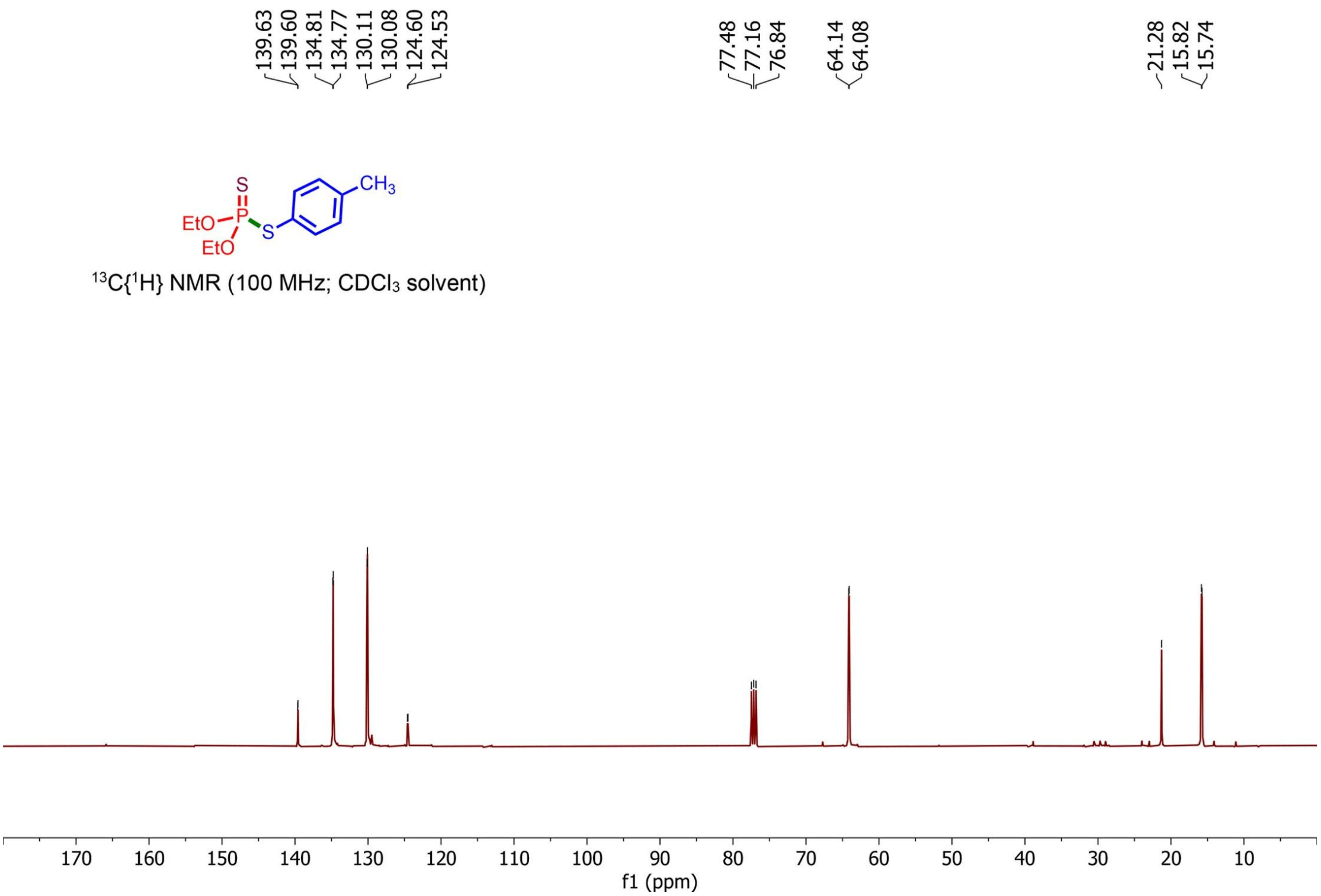

Figure S8. $^{13}C\{^{1}H\}$ NMR spectrum of *O*,*O*-diethyl *S*-(*p*-tolyl) phosphorodithioate (**3b**)

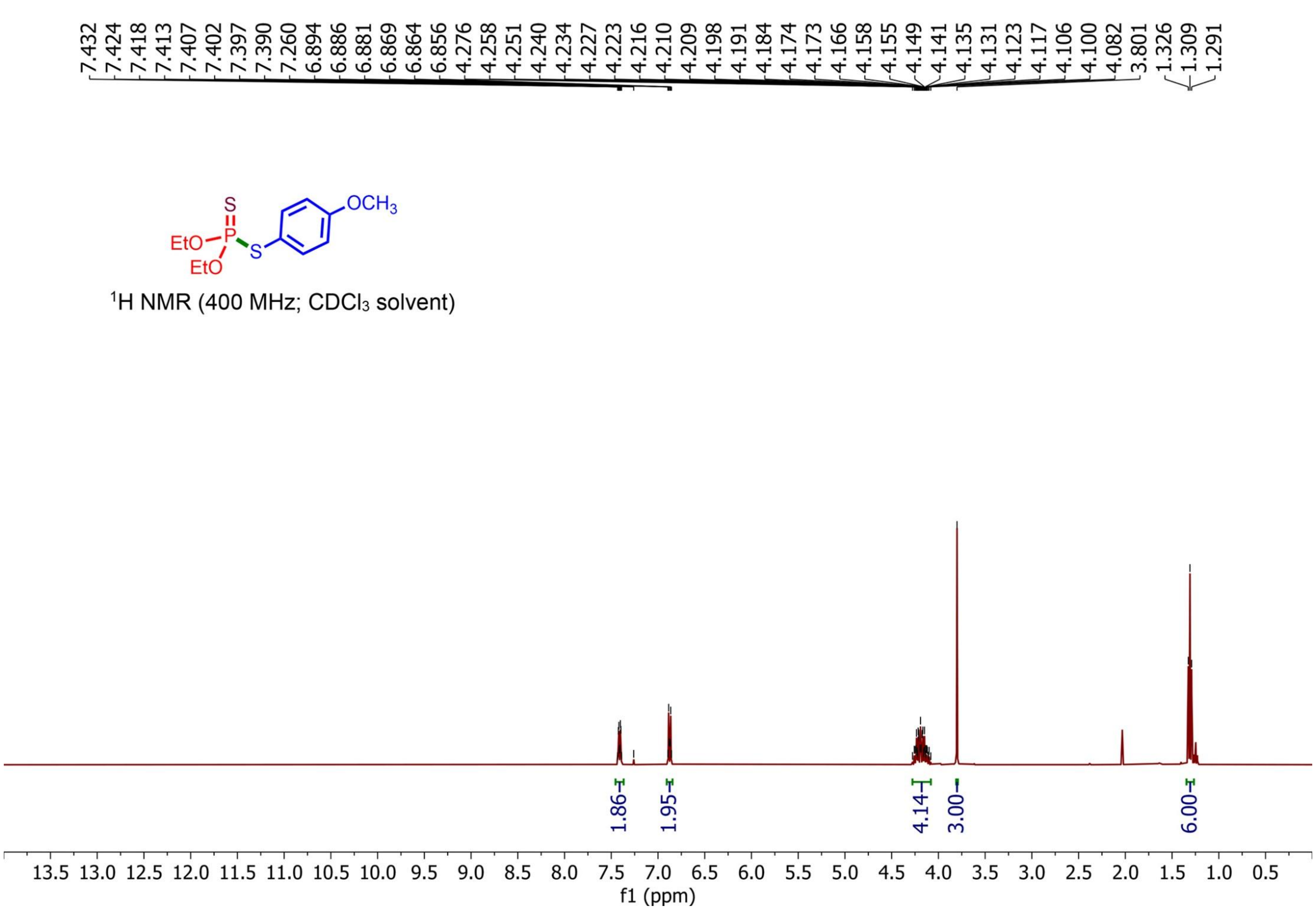

Figure S9. ^{1}H NMR spectrum of *O*,*O*-diethyl *S*-(4-methoxyphenyl) phosphorodithioate (**3c**)

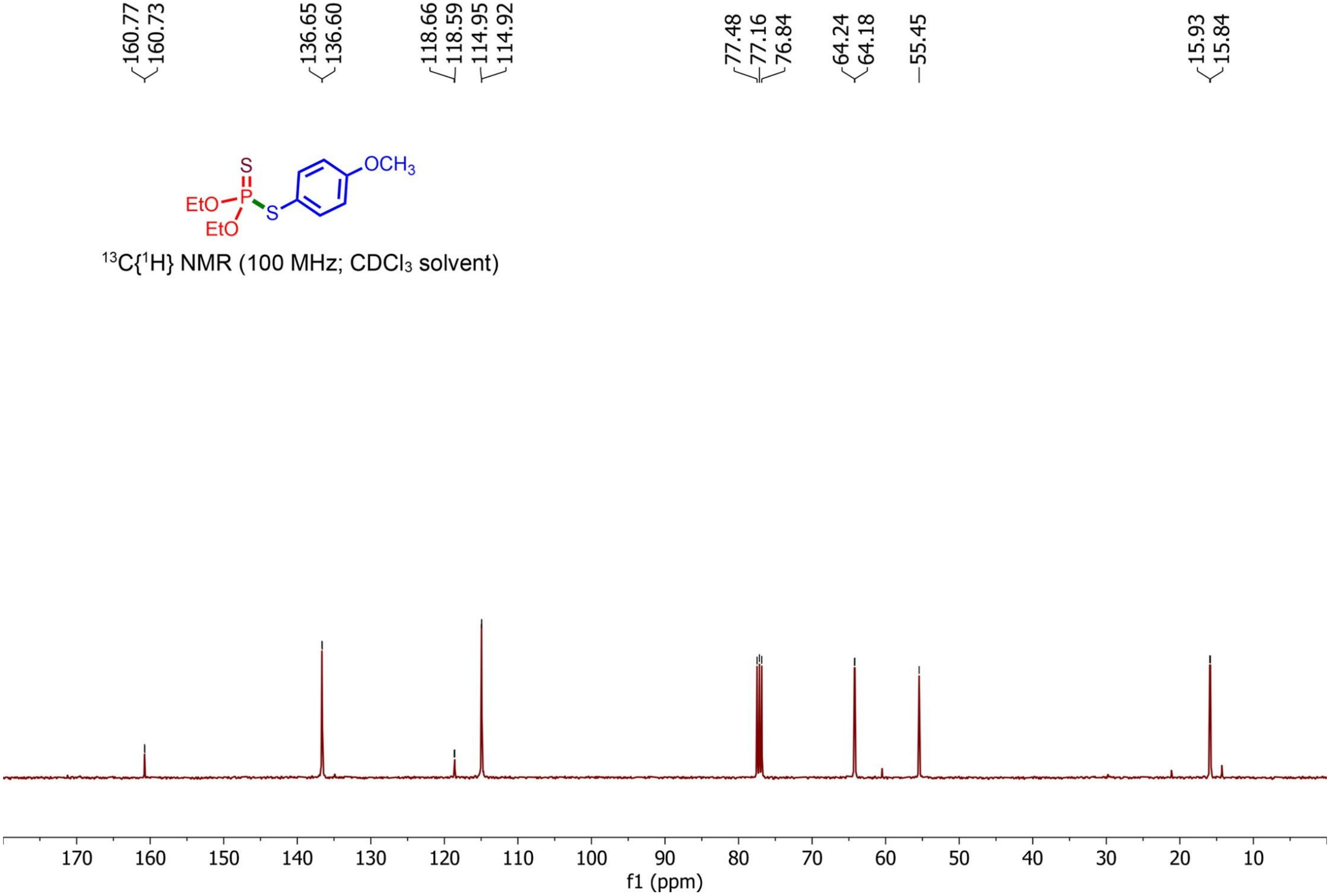

Figure S10. $^{13}C\{^{1}H\}$ NMR spectrum of *O*,*O*-diethyl *S*-(4-methoxyphenyl) phosphorodithioate (**3c**)

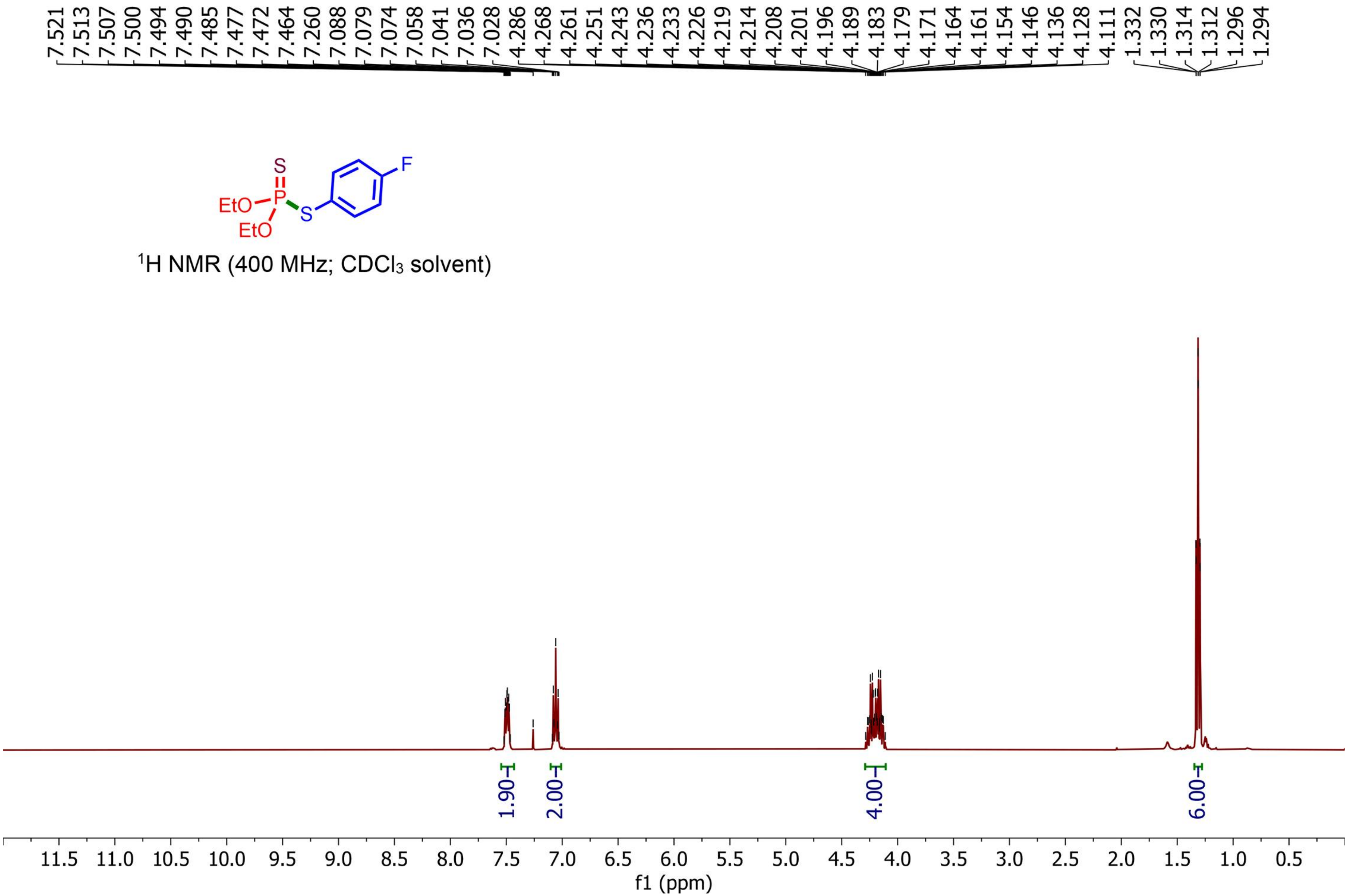

Figure S11. ^{1}H NMR spectrum of *O*,*O*-diethyl *S*-(4-fluorophenyl) phosphorodithioate (**3d**)

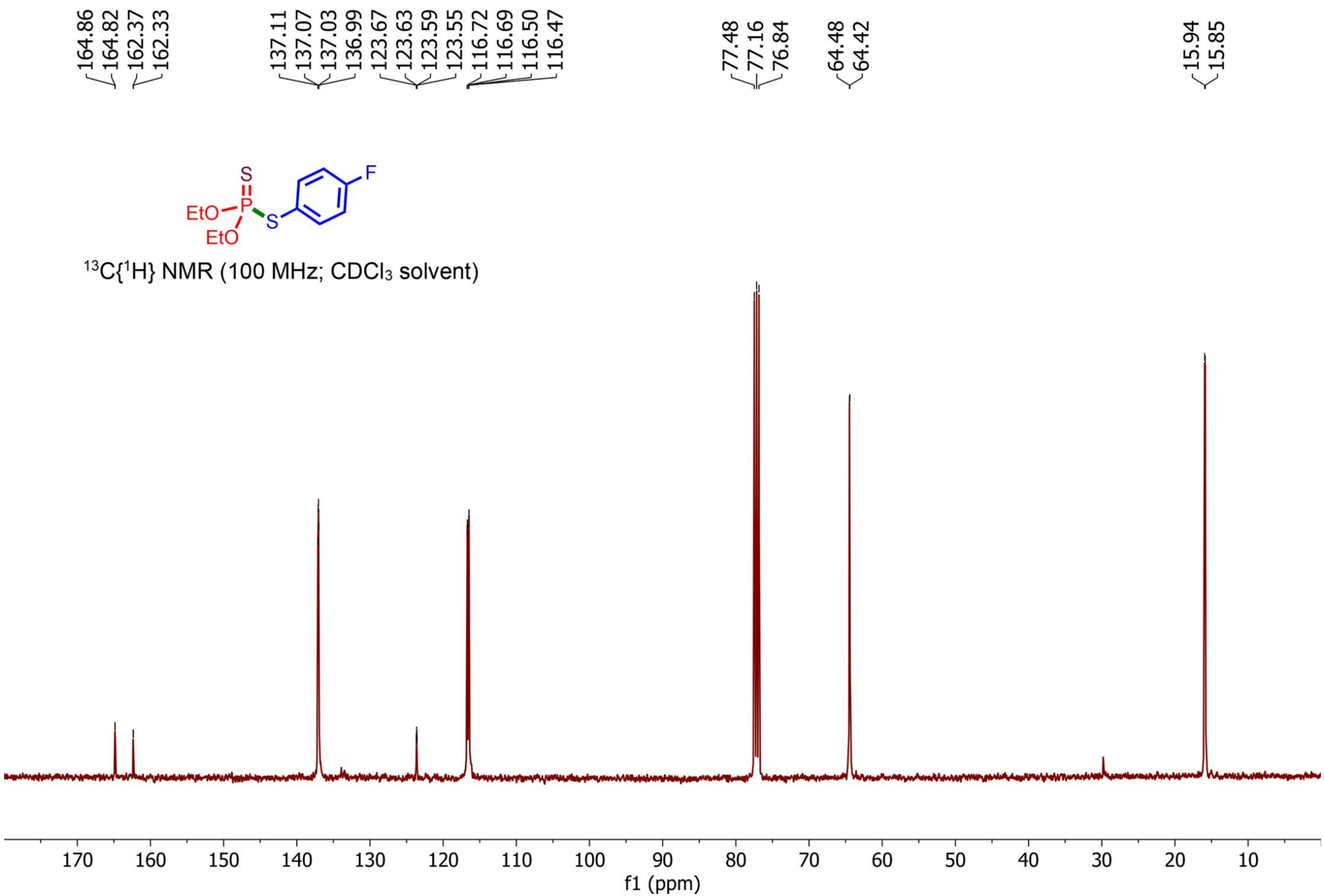

Figure S12. $^{13}C\{^{1}H\}$ NMR spectrum of *O*,*O*-diethyl *S*-(4-fluorophenyl) phosphorodithioate (**3d**)

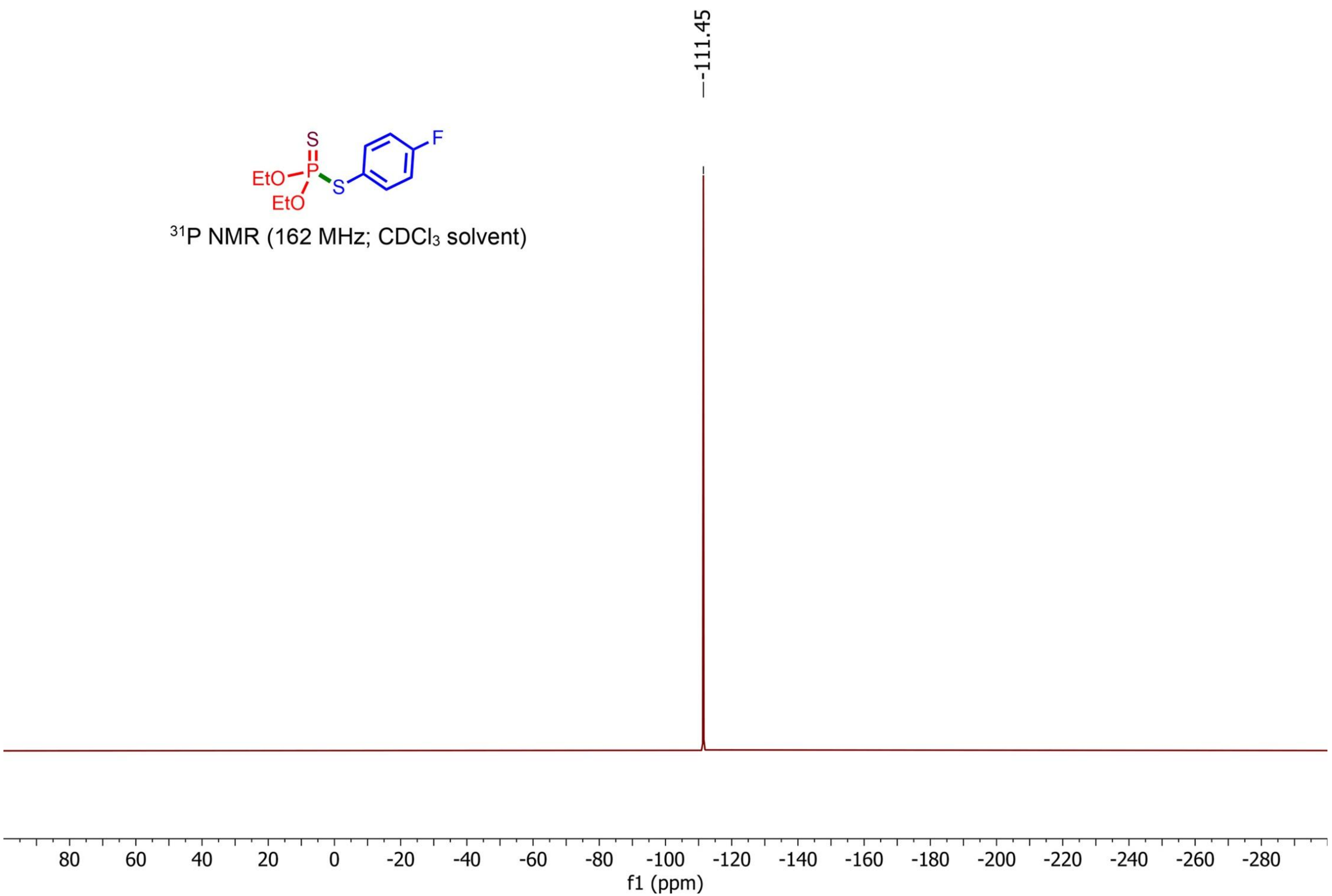

Figure S13. ^{19}F NMR spectrum of *O*,*O*-diethyl *S*-(4-fluorophenyl) phosphorodithioate (**3d**)

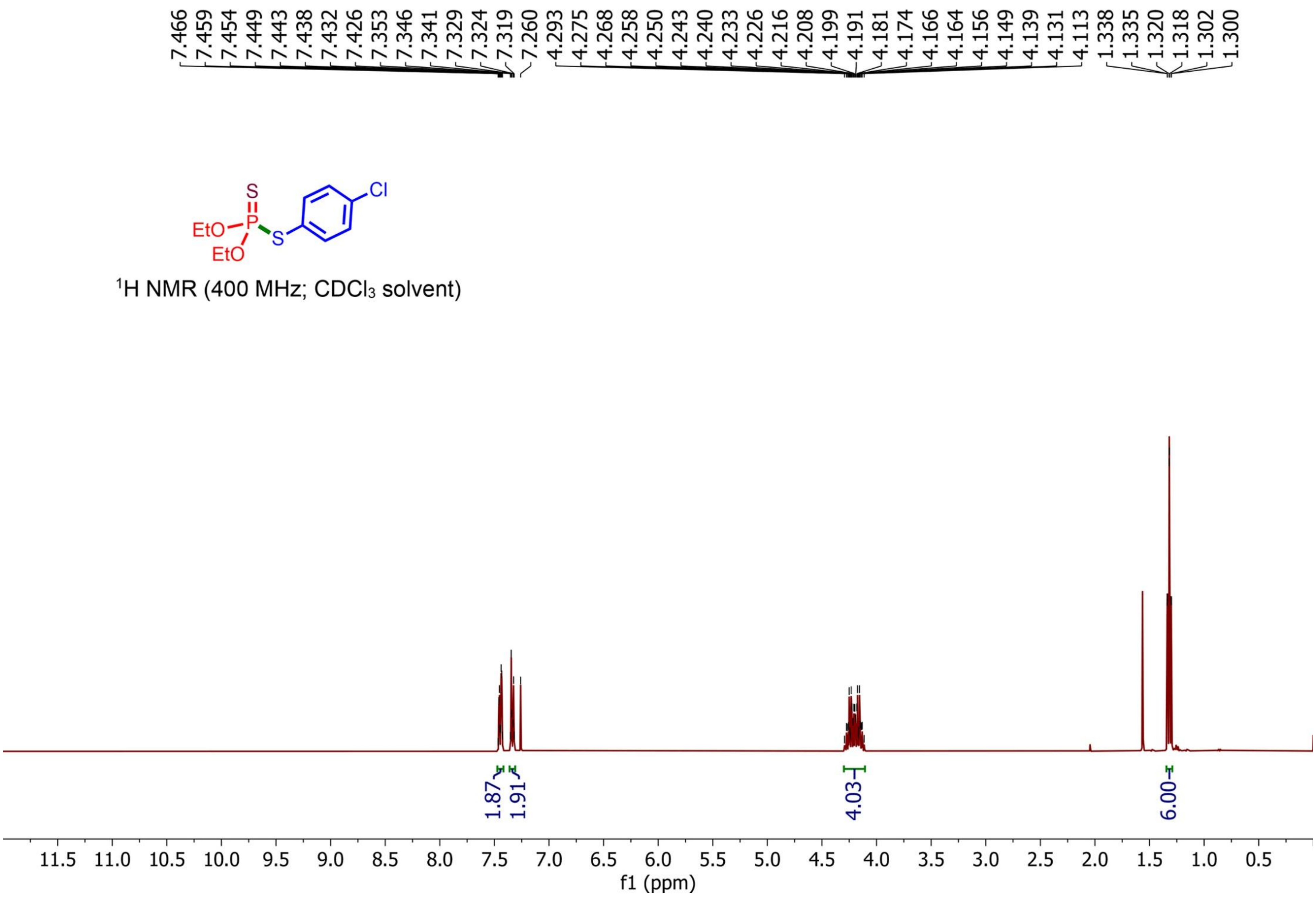

Figure S14. ^{1}H NMR spectrum of *S*-(4-chlorophenyl) *O*,*O*-diethyl phosphorodithioate (**3e**)

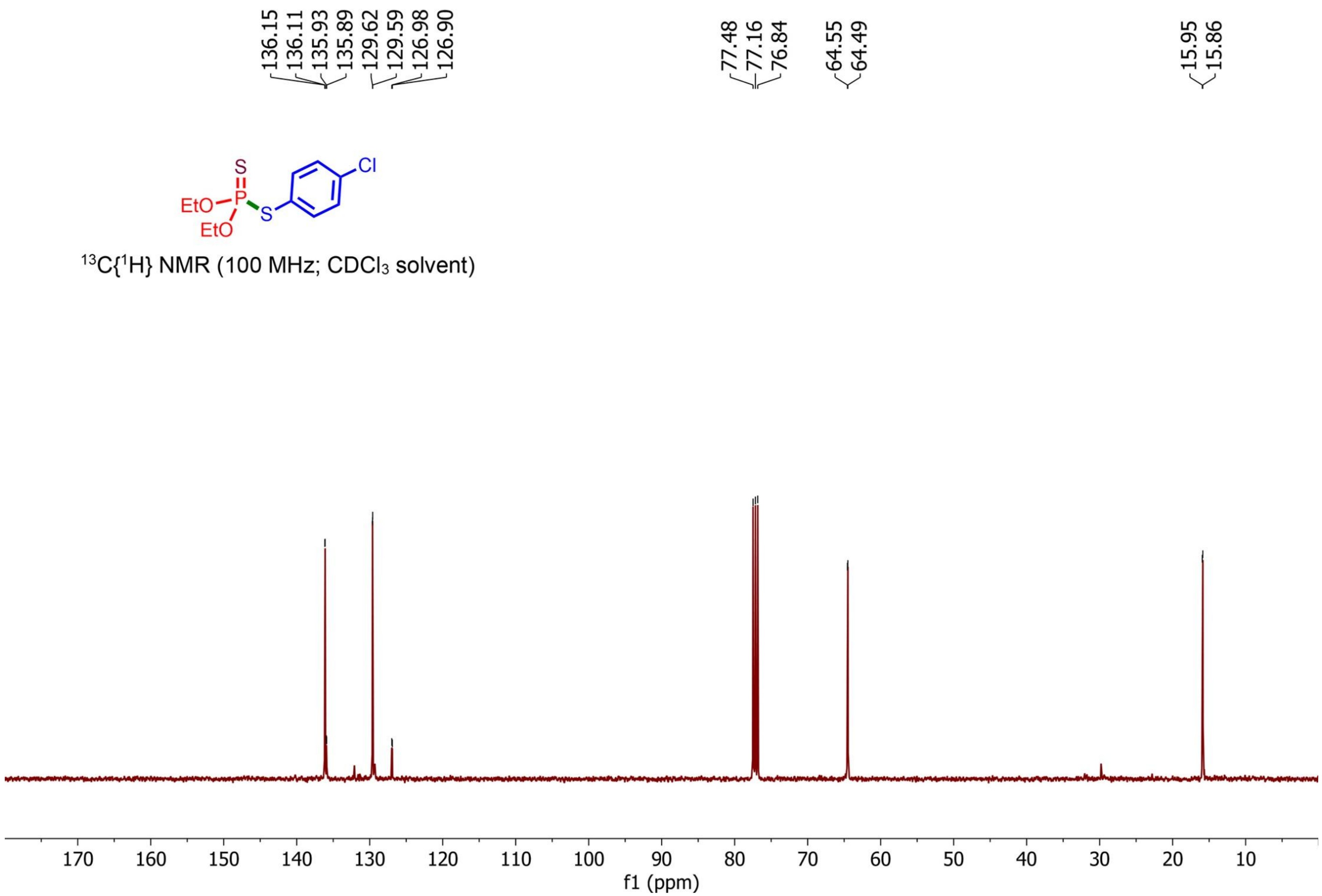

Figure S15. $^{13}C\{^{1}H\}$ NMR spectrum of *S*-(4-chlorophenyl) *O*,*O*-diethyl phosphorodithioate (**3e**)

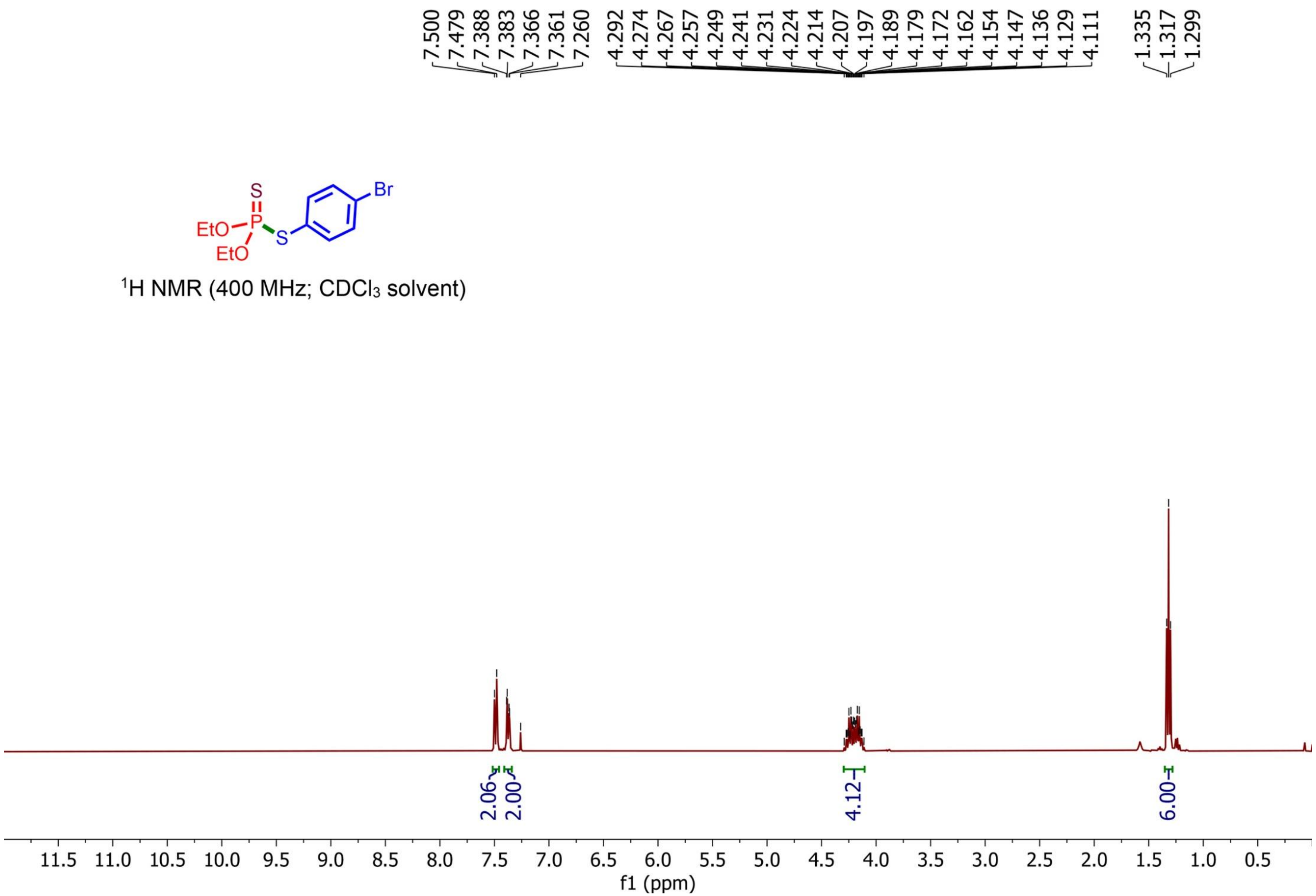

Figure S16. ^{1}H NMR spectrum of *S*-(4-bromophenyl) *O*,*O*-diethyl phosphorodithioate (**3f**)

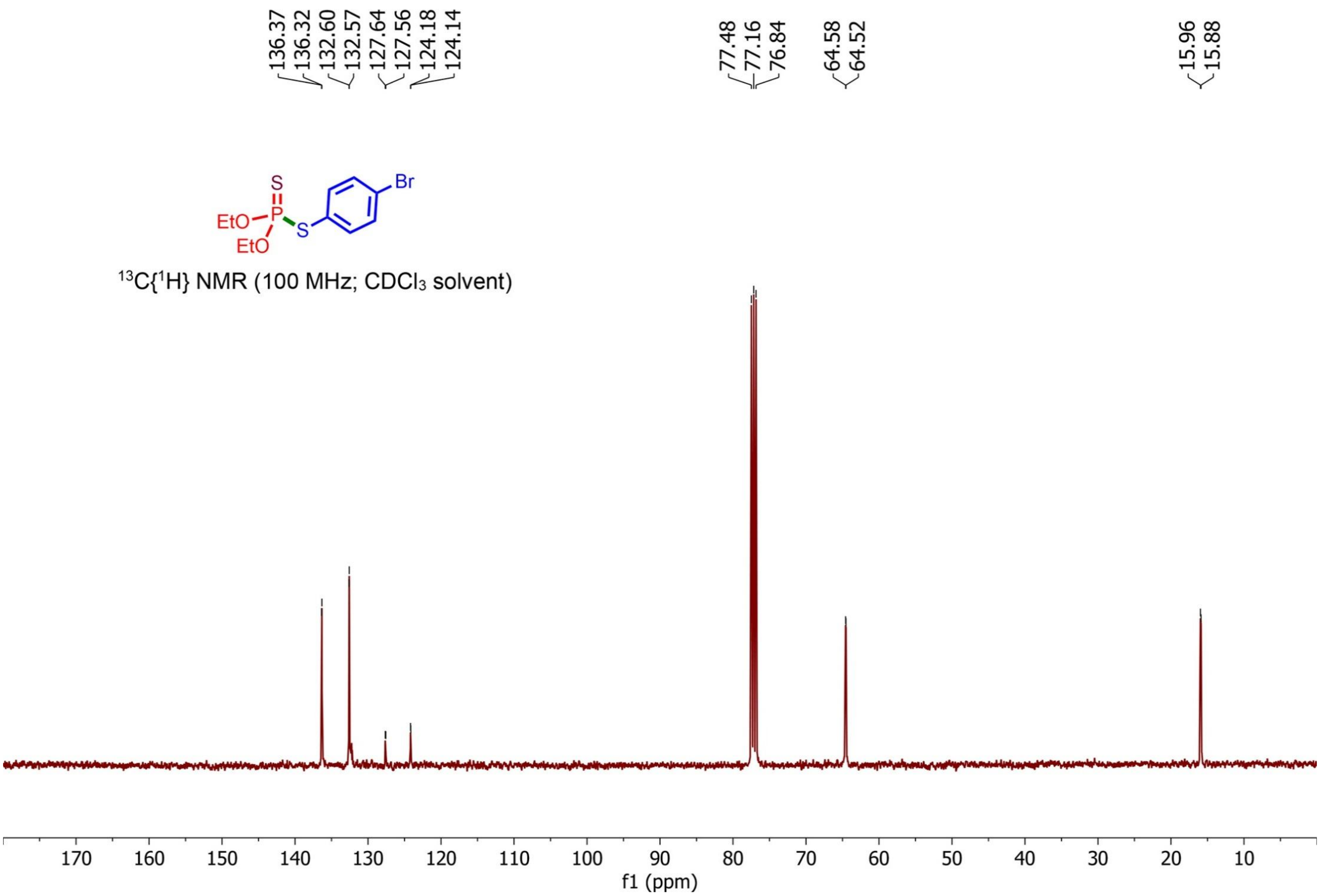

Figure S17. $^{13}C\{^1H\}$ NMR spectrum of *S*-(4-bromophenyl) *O*,*O*-diethyl phosphorodithioate (**3f**)

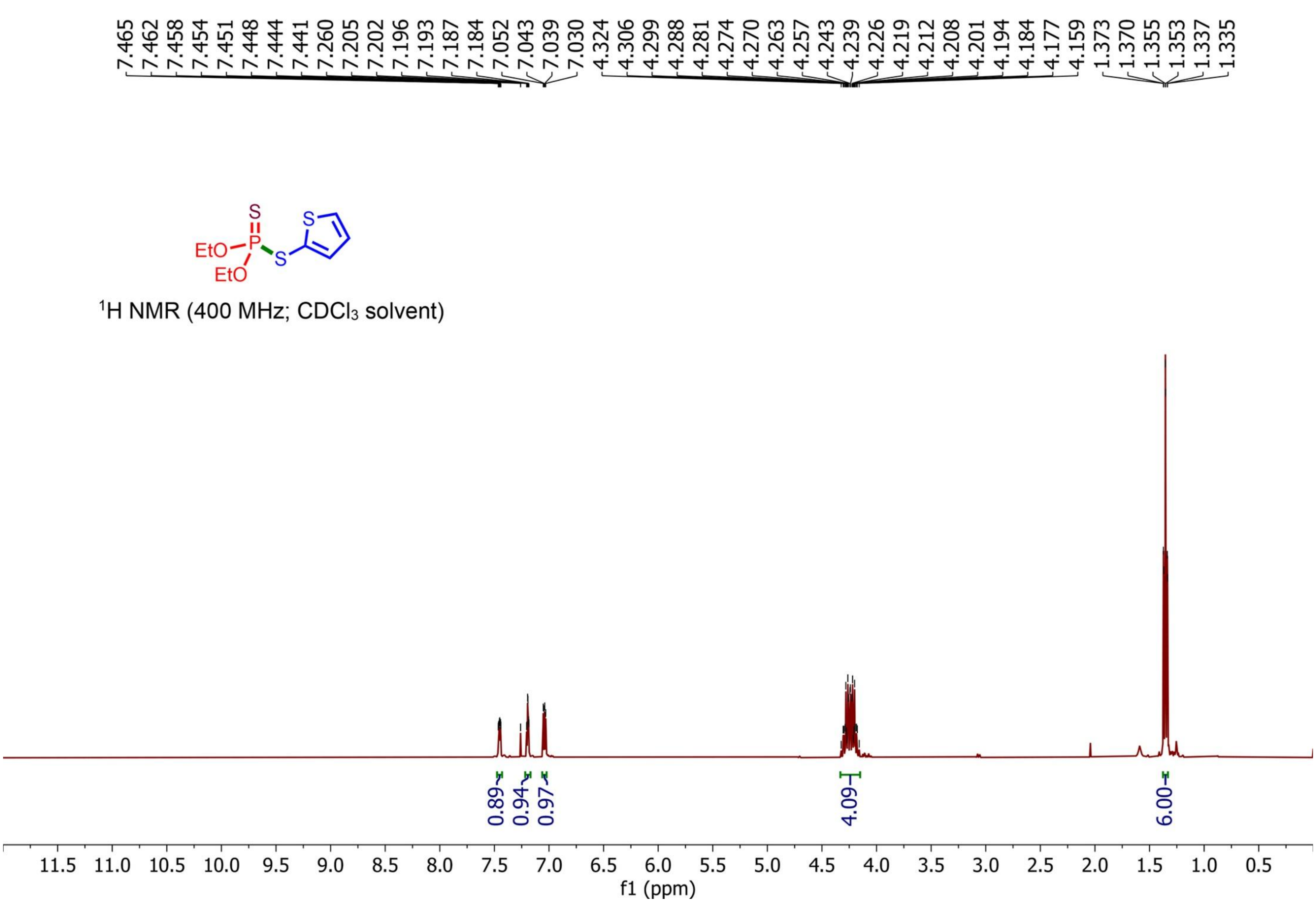

Figure S18. ^{1}H NMR spectrum of *O*,*O*-diethyl *S*-(thiophen-2-yl) phosphorodithioate (**3g**)

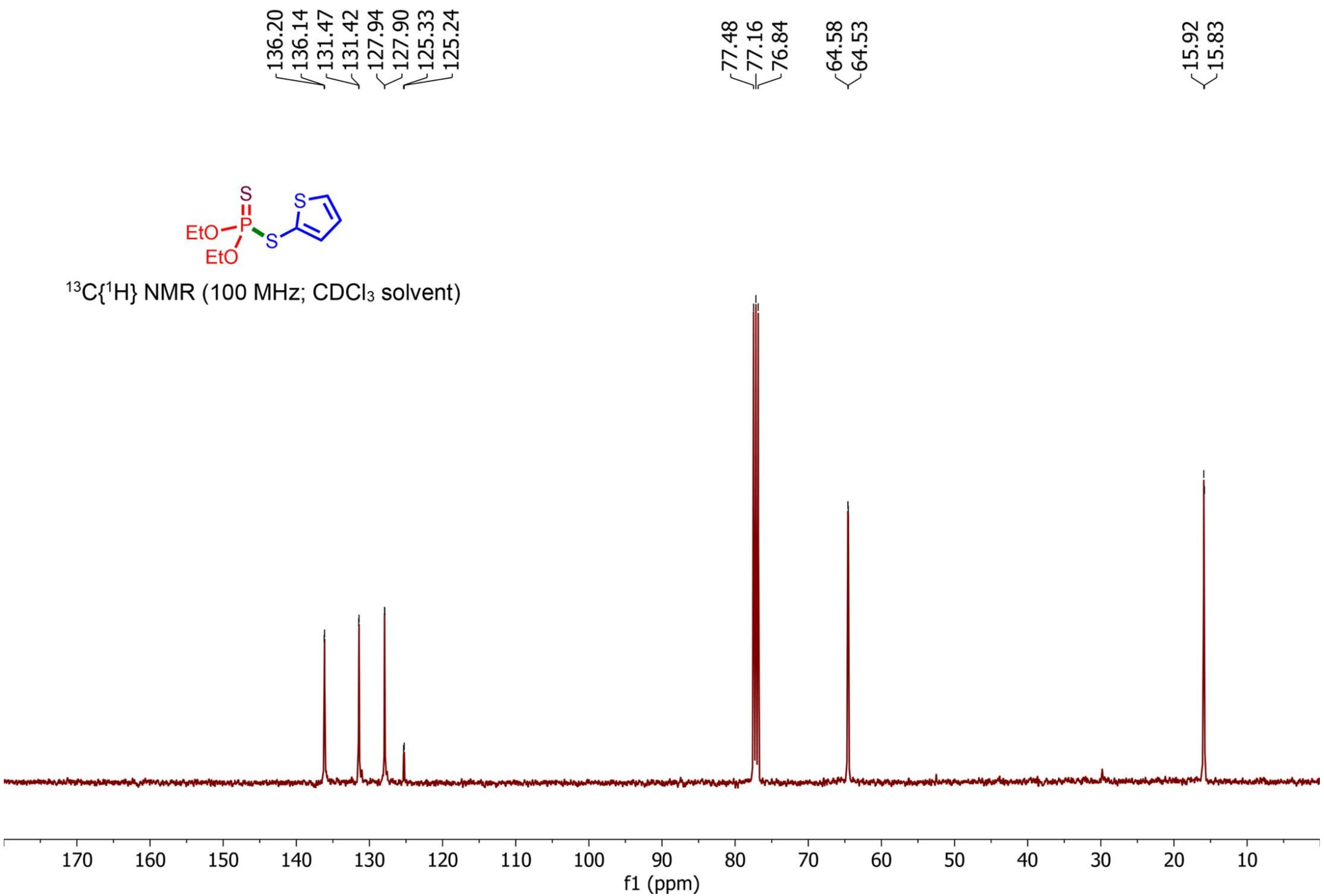

Figure S19. $^{13}C\{^1H\}$ NMR spectrum of *O*,*O*-diethyl *S*-(thiophen-2-yl) phosphorodithioate (**3g**)

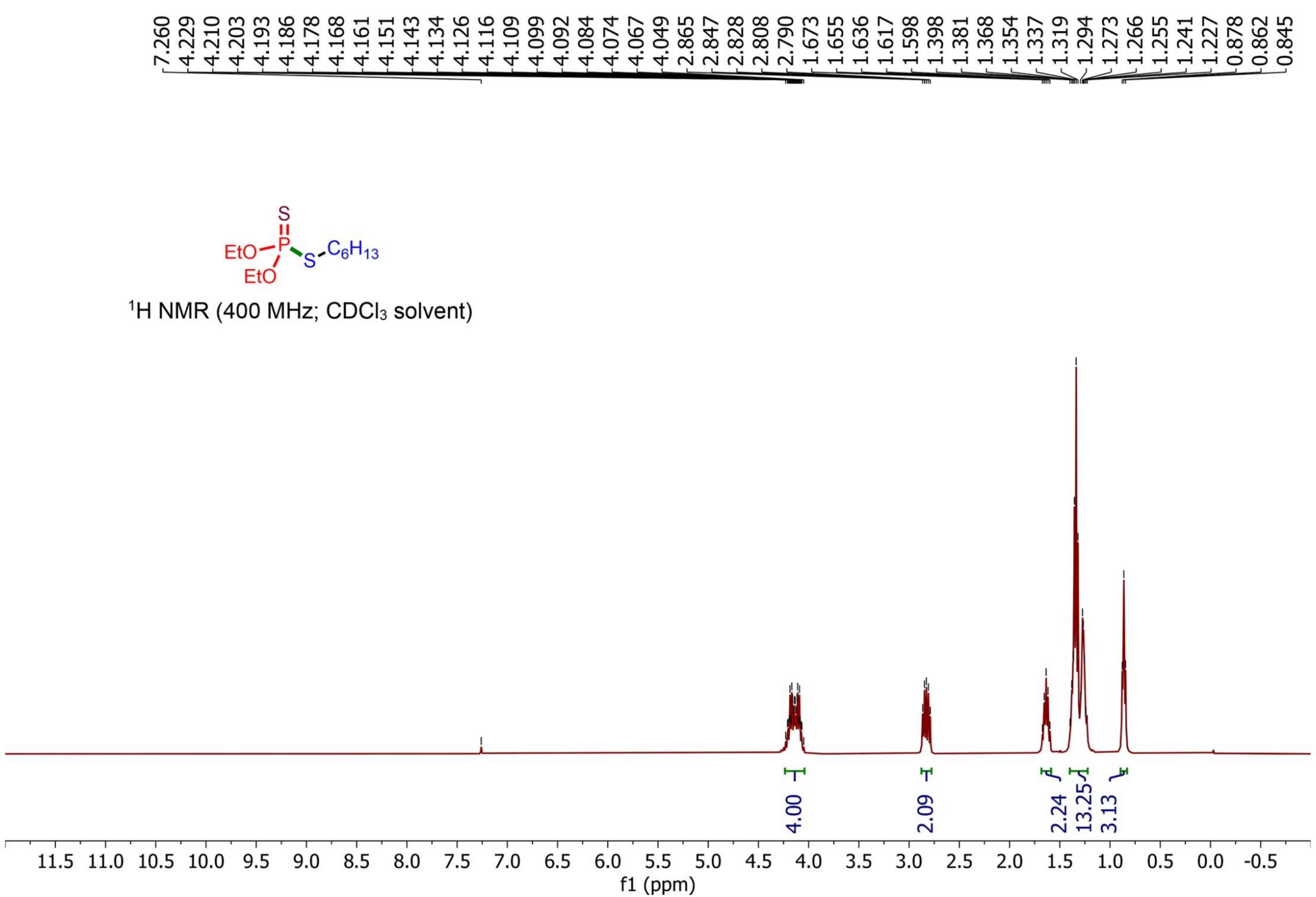

Figure S20. ^{1}H NMR spectrum of *O*,*O*-diethyl *S*-hexyl phosphorodithioate (**3h**)

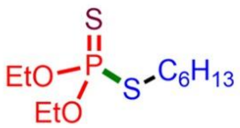

$^{13}C\{^1H\}$ NMR (100 MHz; $CDCl_3$ solvent)

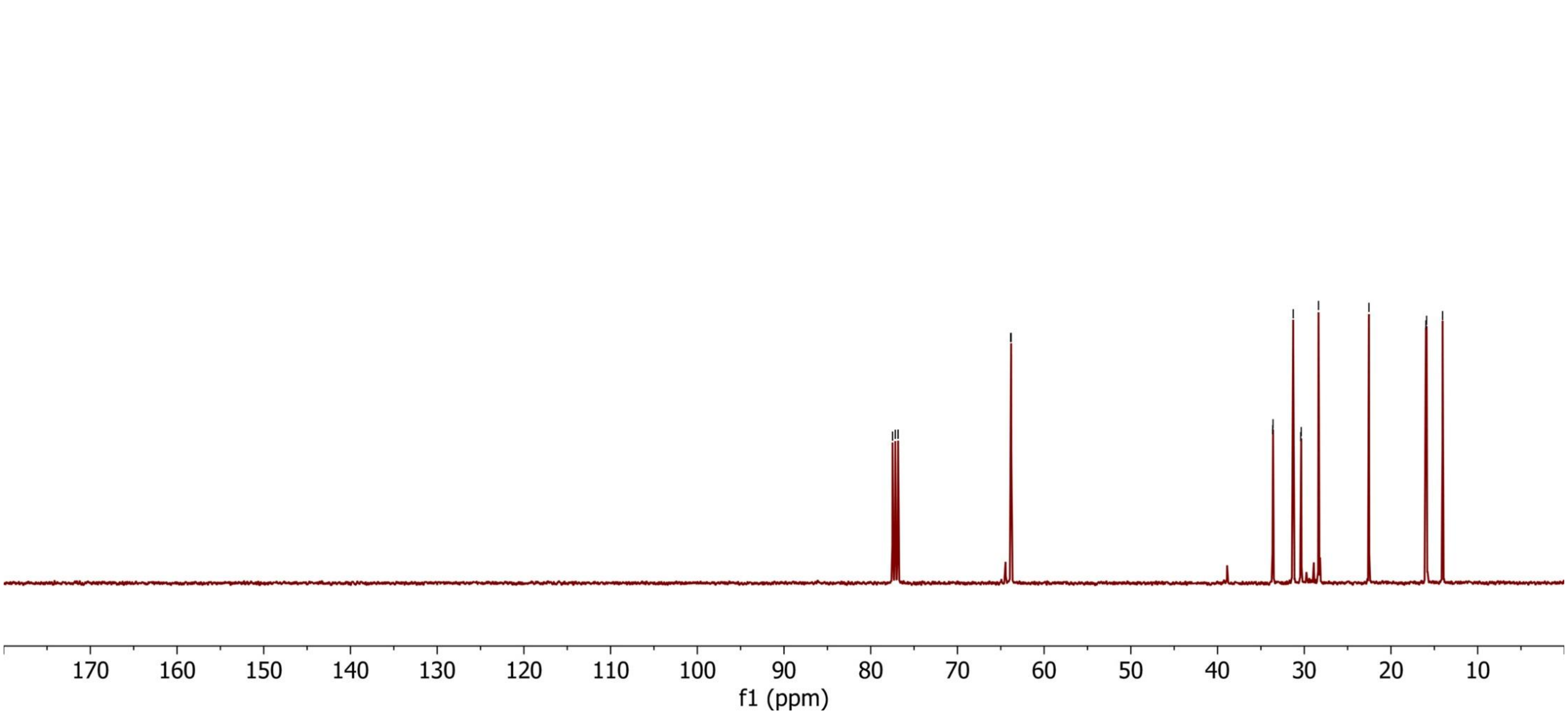

Figure S21. $^{13}C\{^1H\}$ NMR spectrum of *O*,*O*-diethyl *S*-hexyl phosphorodithioate (**3h**)

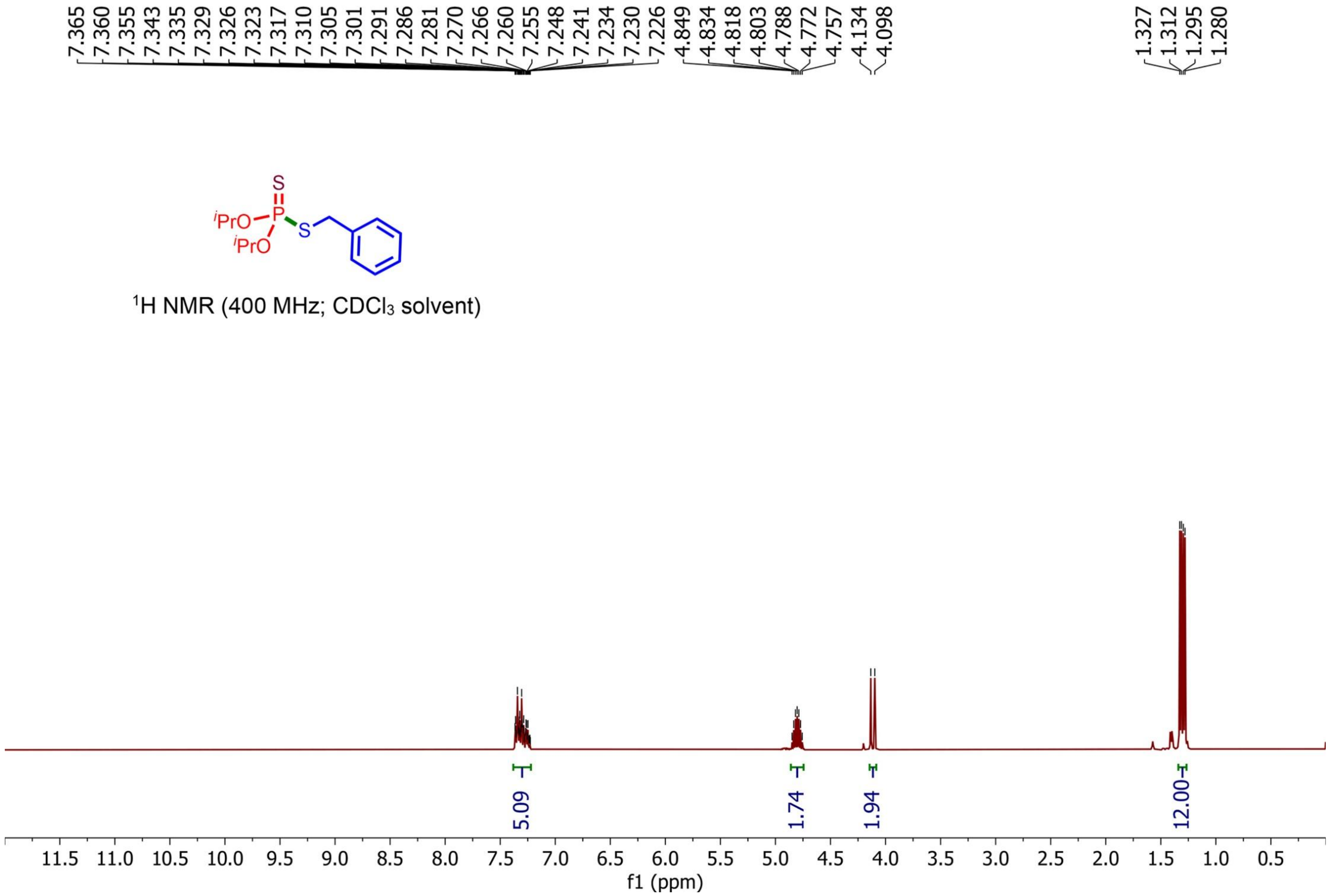

Figure S22. ^{1}H NMR spectrum of *S*-benzyl *O*,*O*-diisopropyl phosphorodithioate (**3i**)

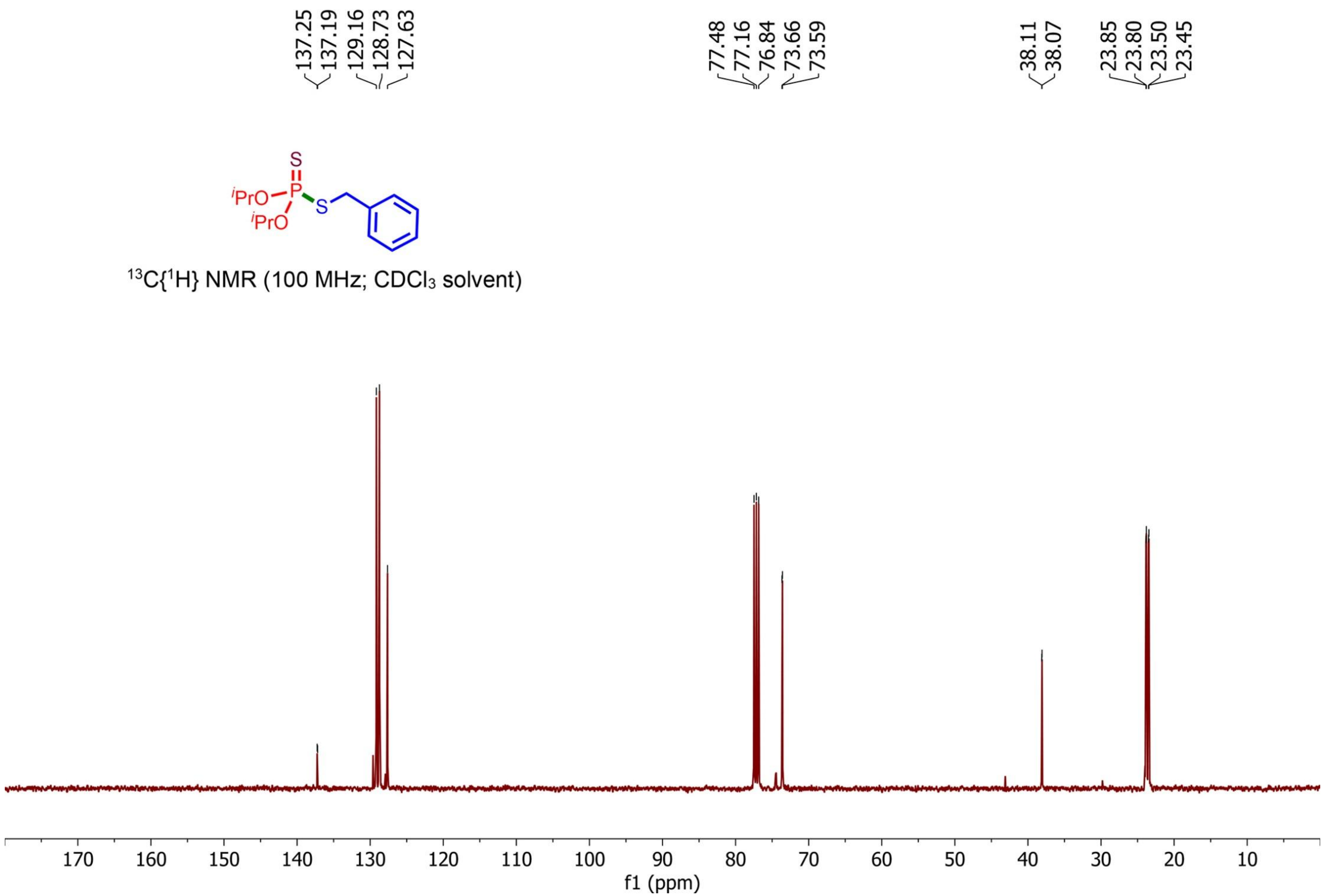

Figure S23. $^{13}C\{^{1}H\}$ NMR spectrum of *S*-benzyl *O*,*O*-diisopropyl phosphorodithioate (**3i**)

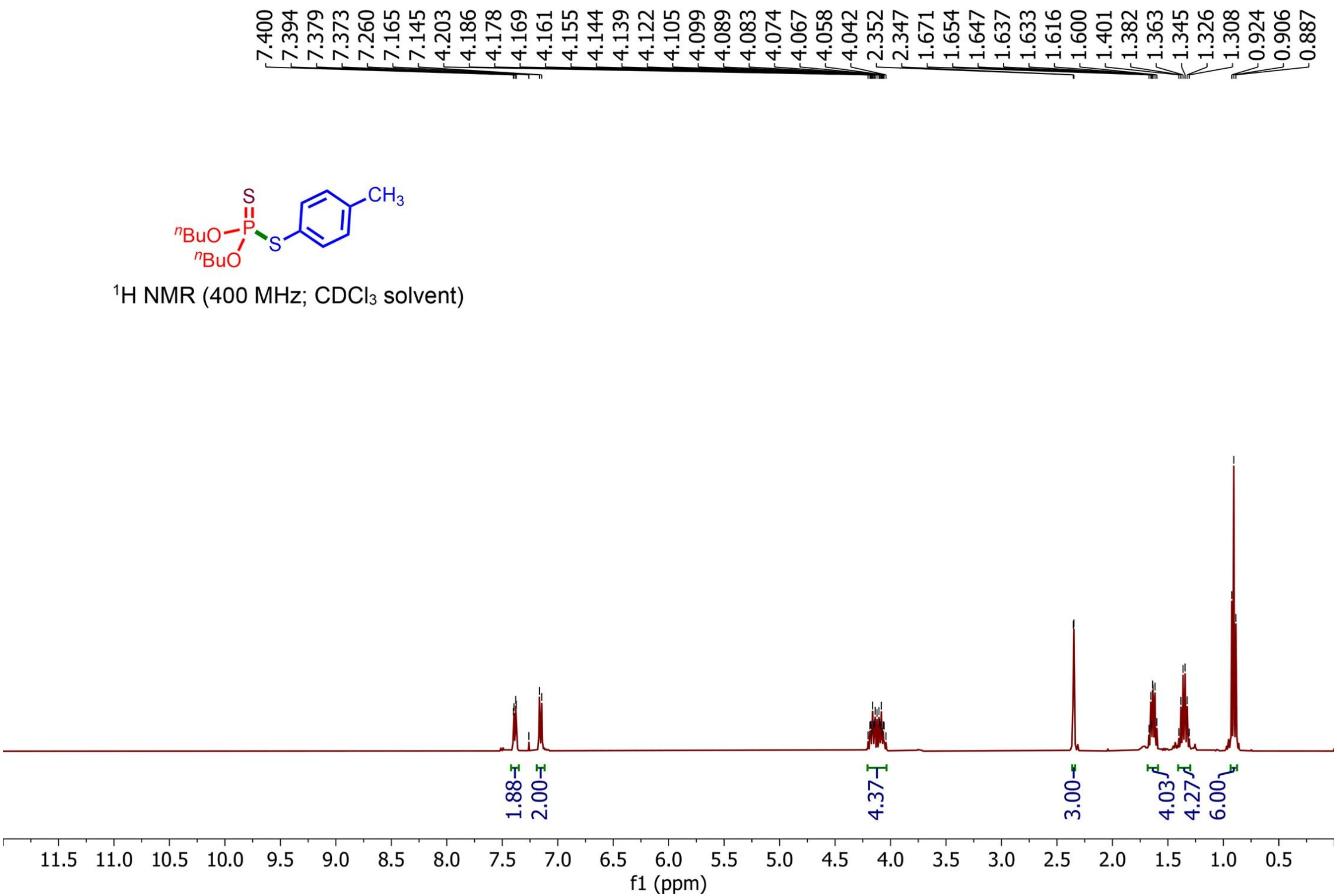

Figure S24. ^{1}H NMR spectrum of *O*,*O*-dibutyl *S*-(*p*-tolyl) phosphorodithioate (**3j**)

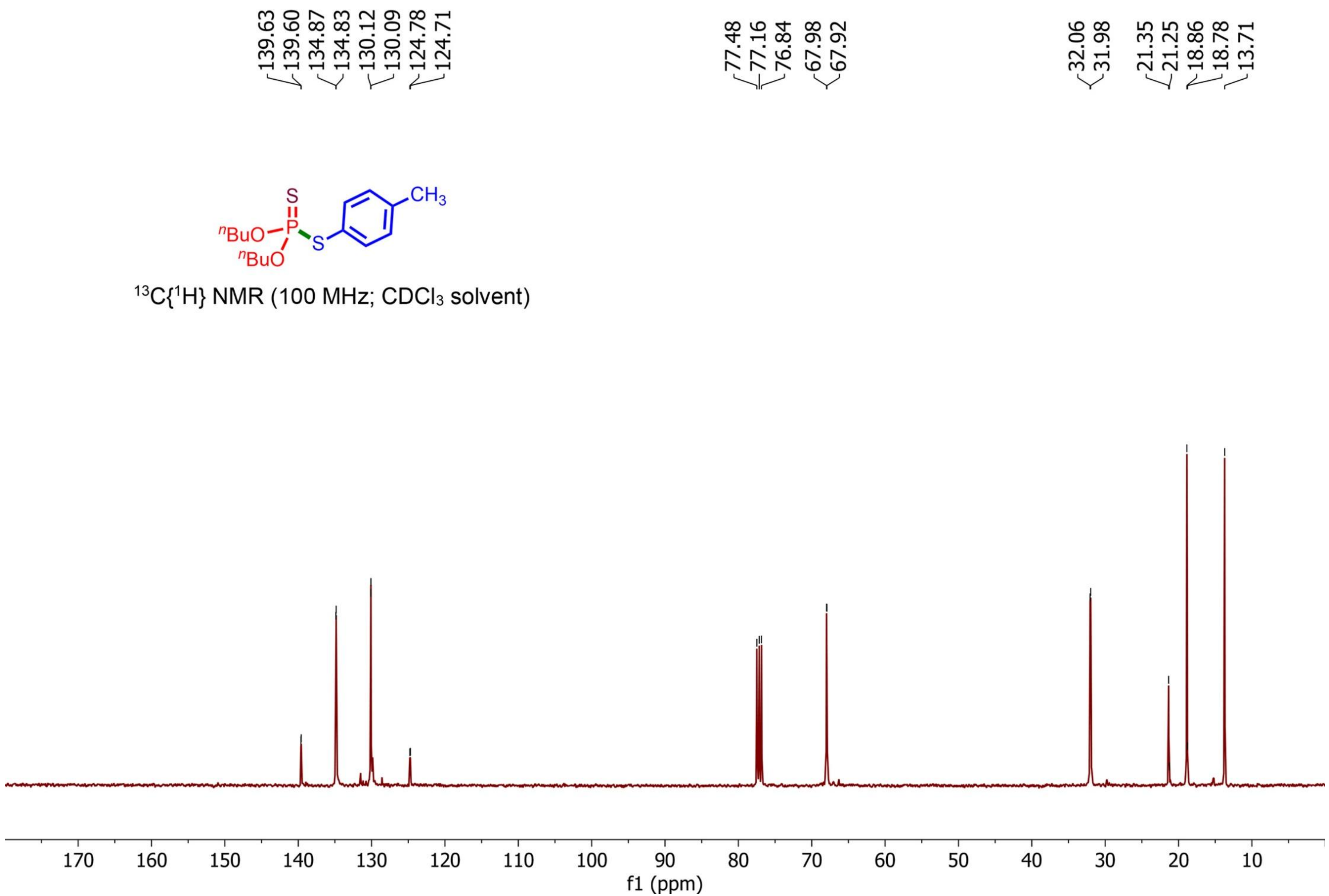

Figure S25. $^{13}C\{^{1}H\}$ NMR spectrum of *O*,*O*-dibutyl *S*-(*p*-tolyl) phosphorodithioate (**3j**)

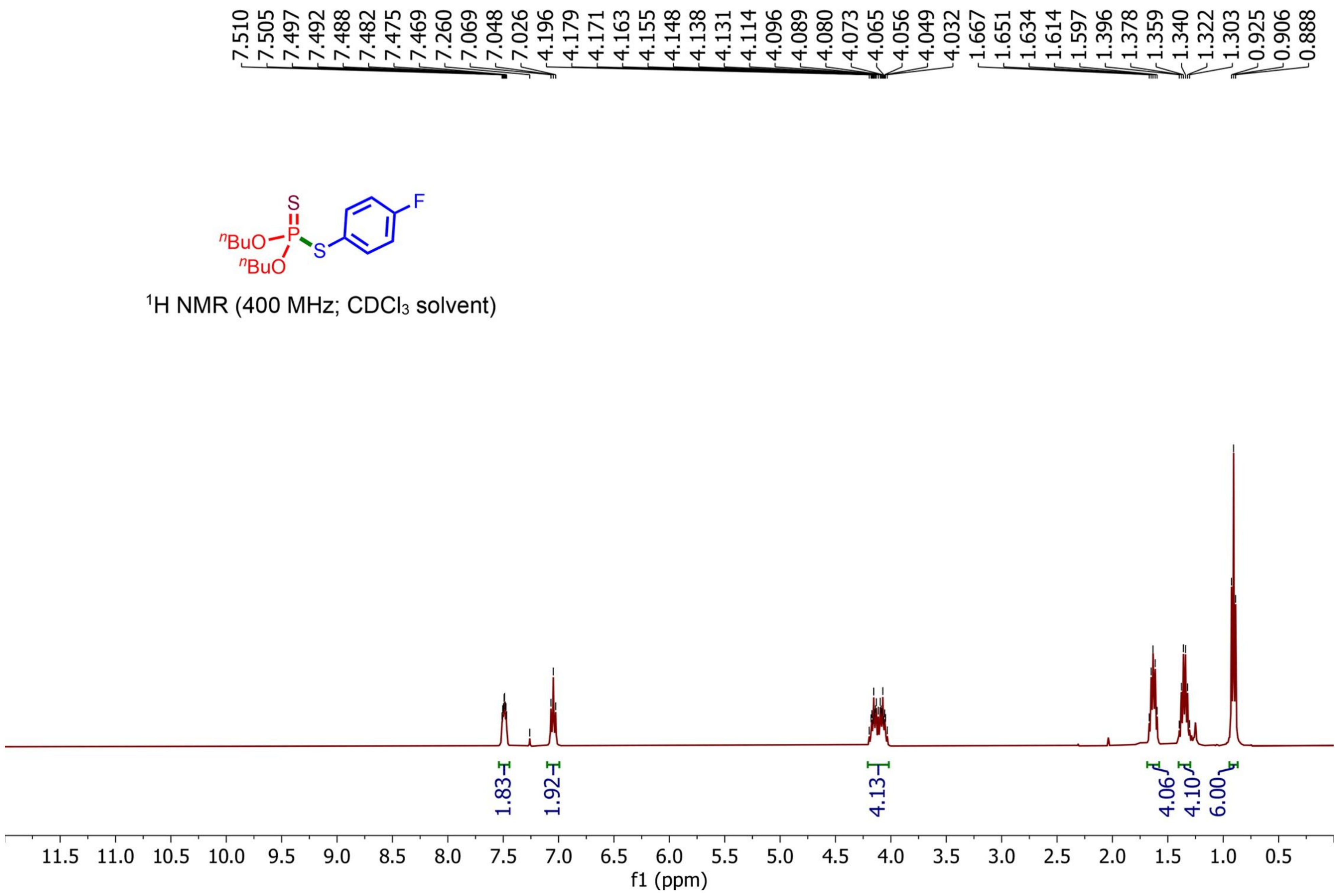

Figure S26. ^{1}H NMR spectrum of *O*,*O*-dibutyl *S*-(4-fluorophenyl) phosphorodithioate (**3k**)

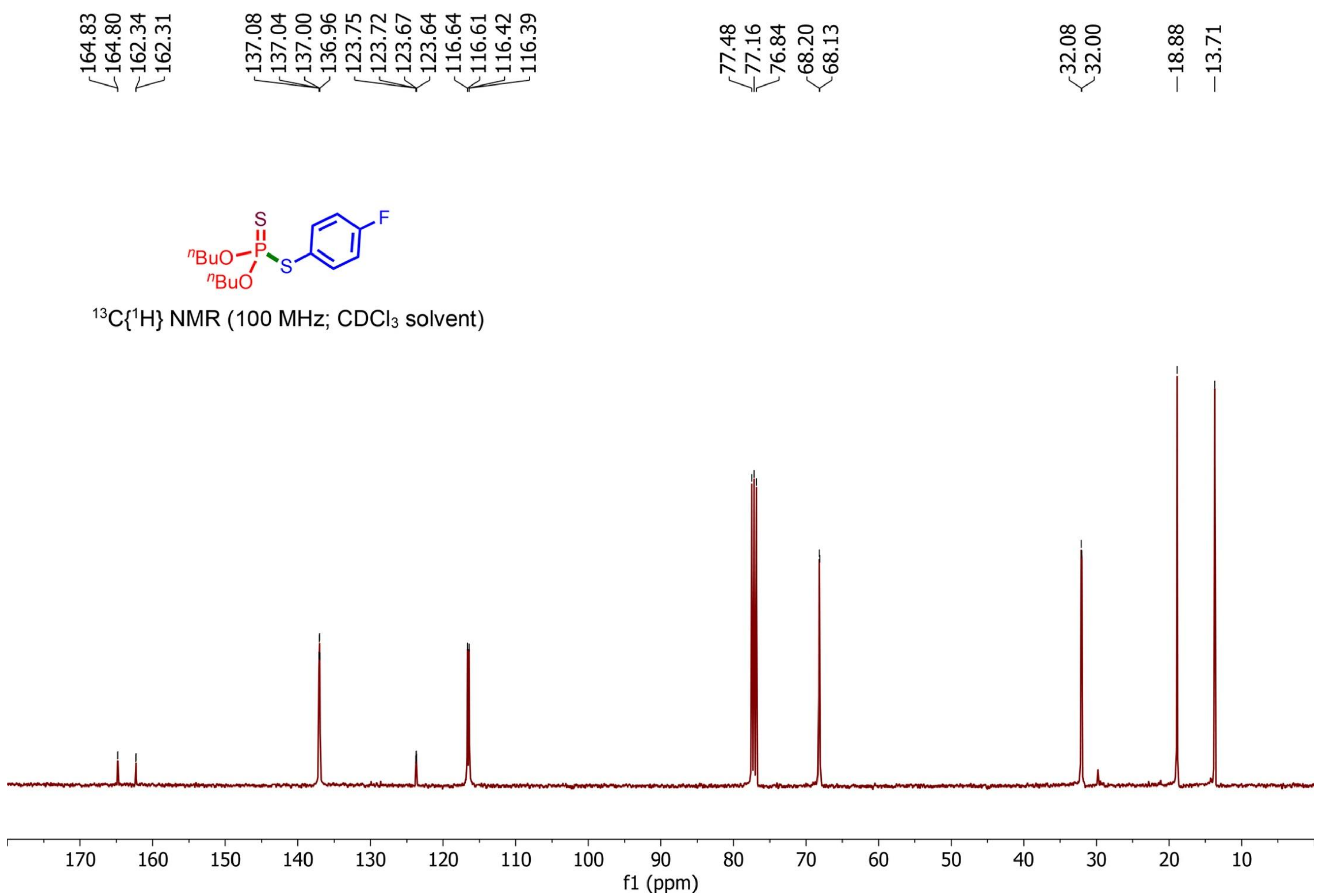

Figure S27. $^{13}C\{^1H\}$ NMR spectrum of *O*,*O*-dibutyl *S*-(4-fluorophenyl) phosphorodithioate (**3k**)

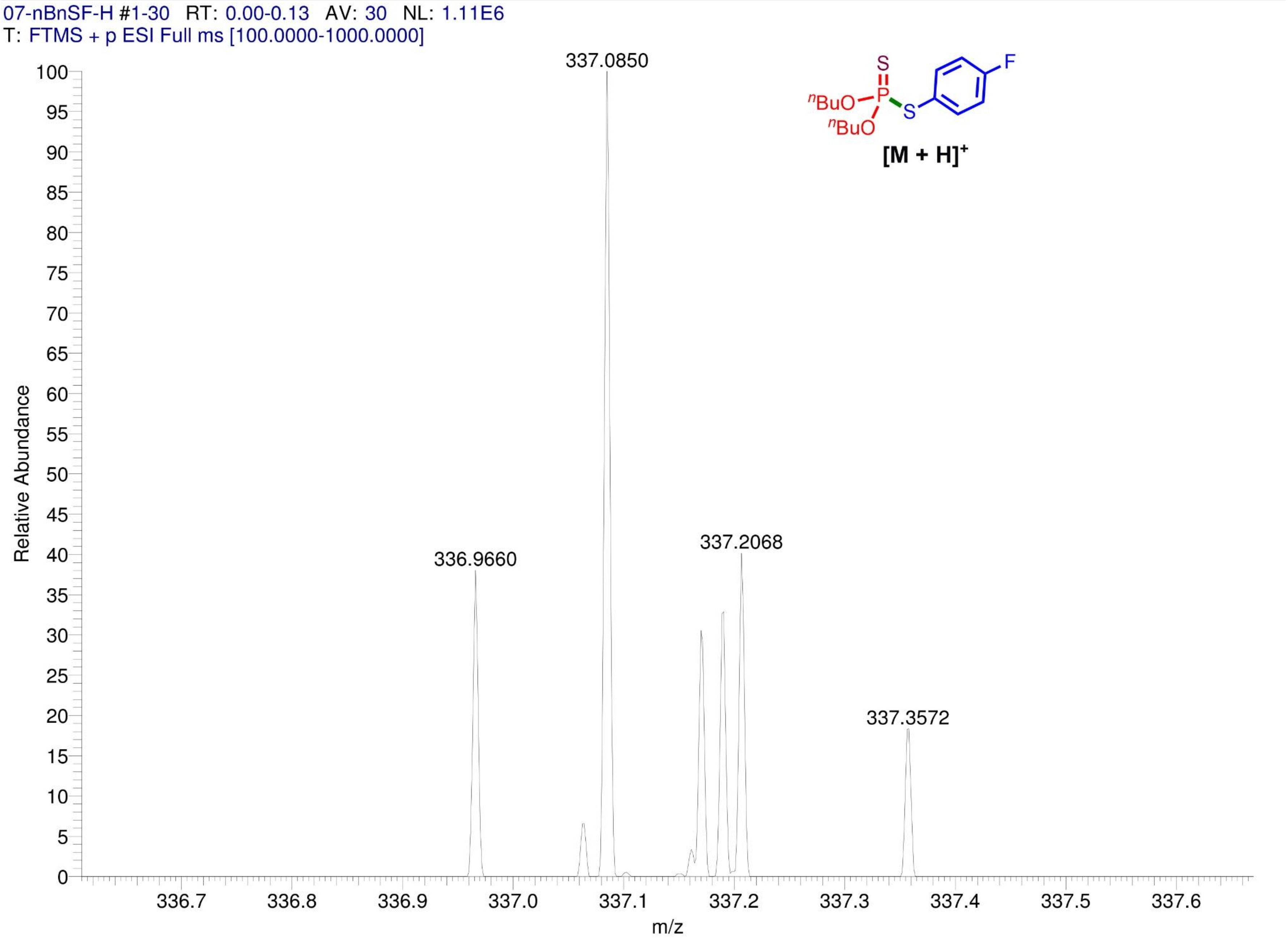

Figure S28. HRMS spectrum of *O*,*O*-dibutyl *S*-(4-fluorophenyl) phosphorodithioate (**3k**)

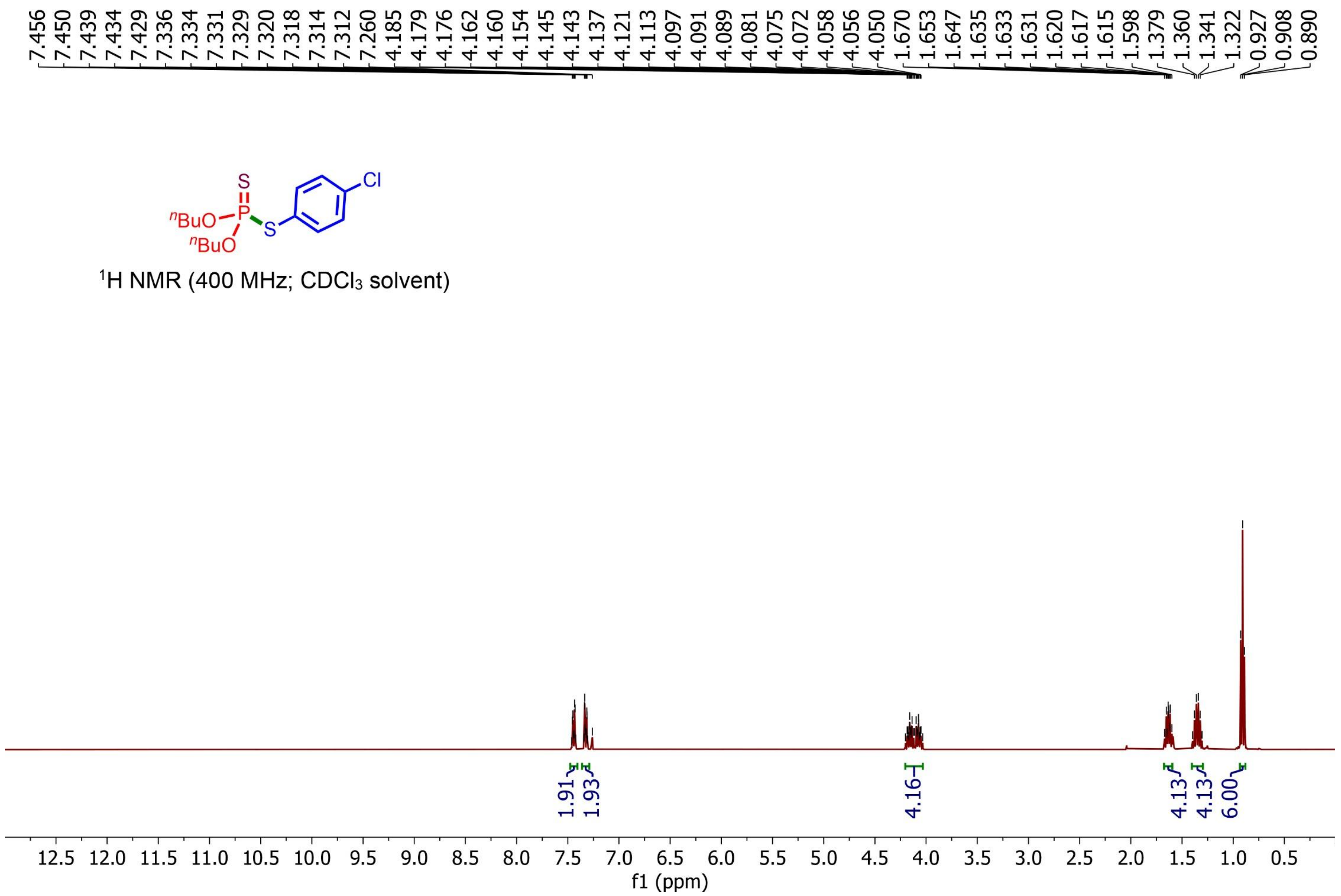

Figure S29. ^{1}H NMR spectrum of *O*,*O*-dibutyl *S*-(4-chlorophenyl) phosphorodithioate (**3l**)

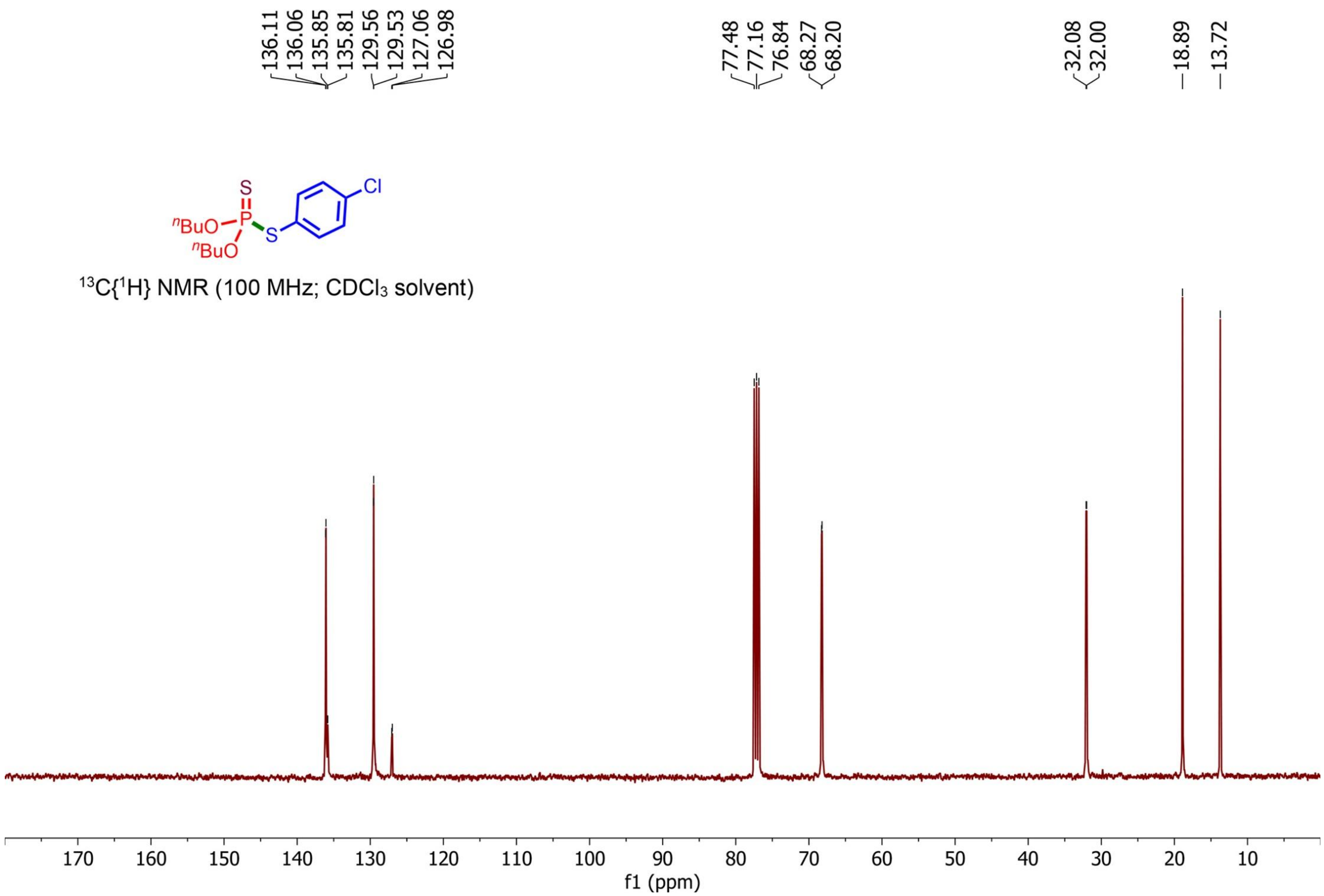

Figure S30. $^{13}C\{^{1}H\}$ NMR spectrum of *O*,*O*-dibutyl *S*-(4-chlorophenyl) phosphorodithioate (**3l**)

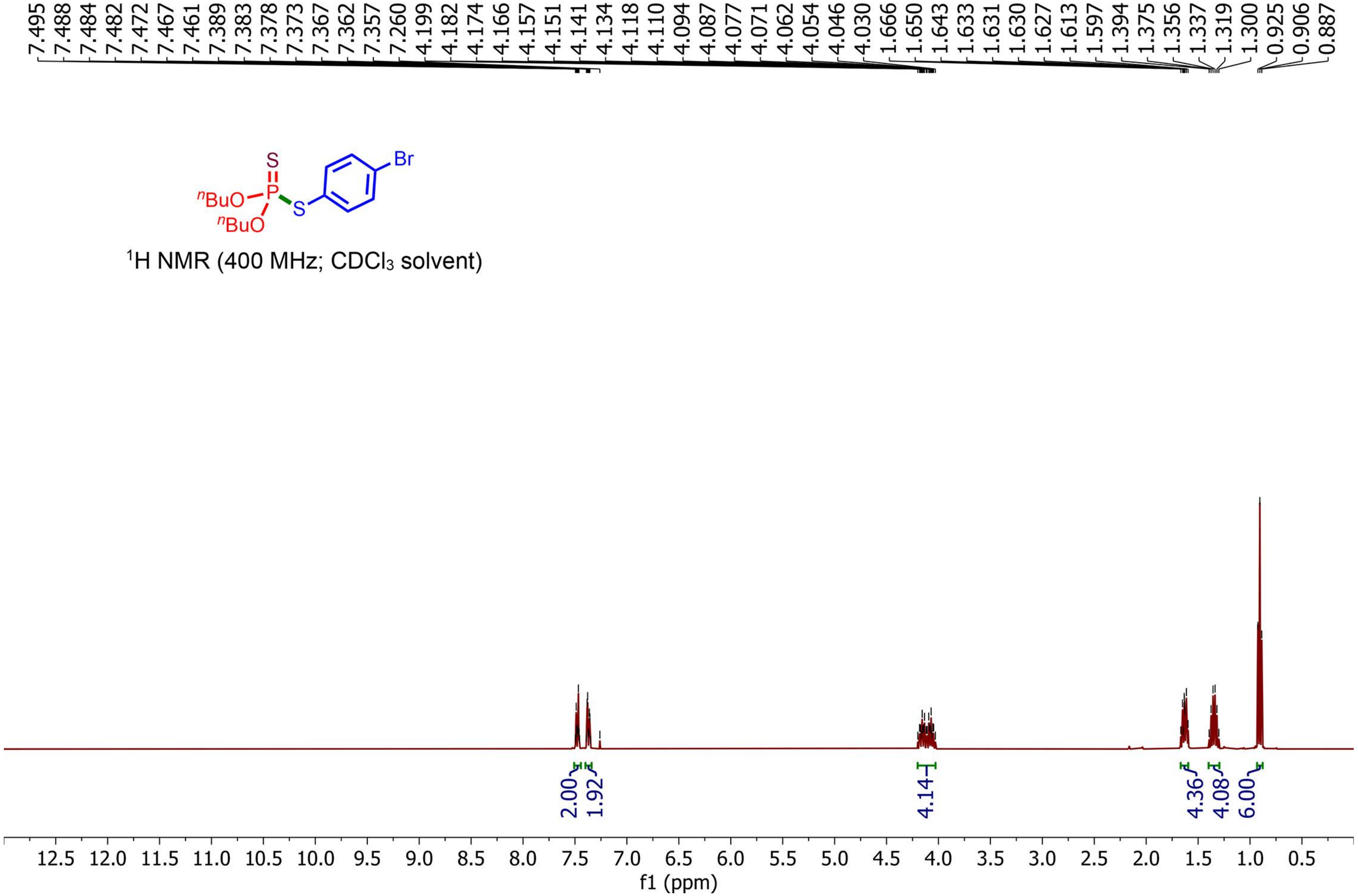

Figure S31. ^{1}H NMR spectrum of *S*-(4-bromophenyl) *O*,*O*-dibutyl phosphorodithioate (**3m**)

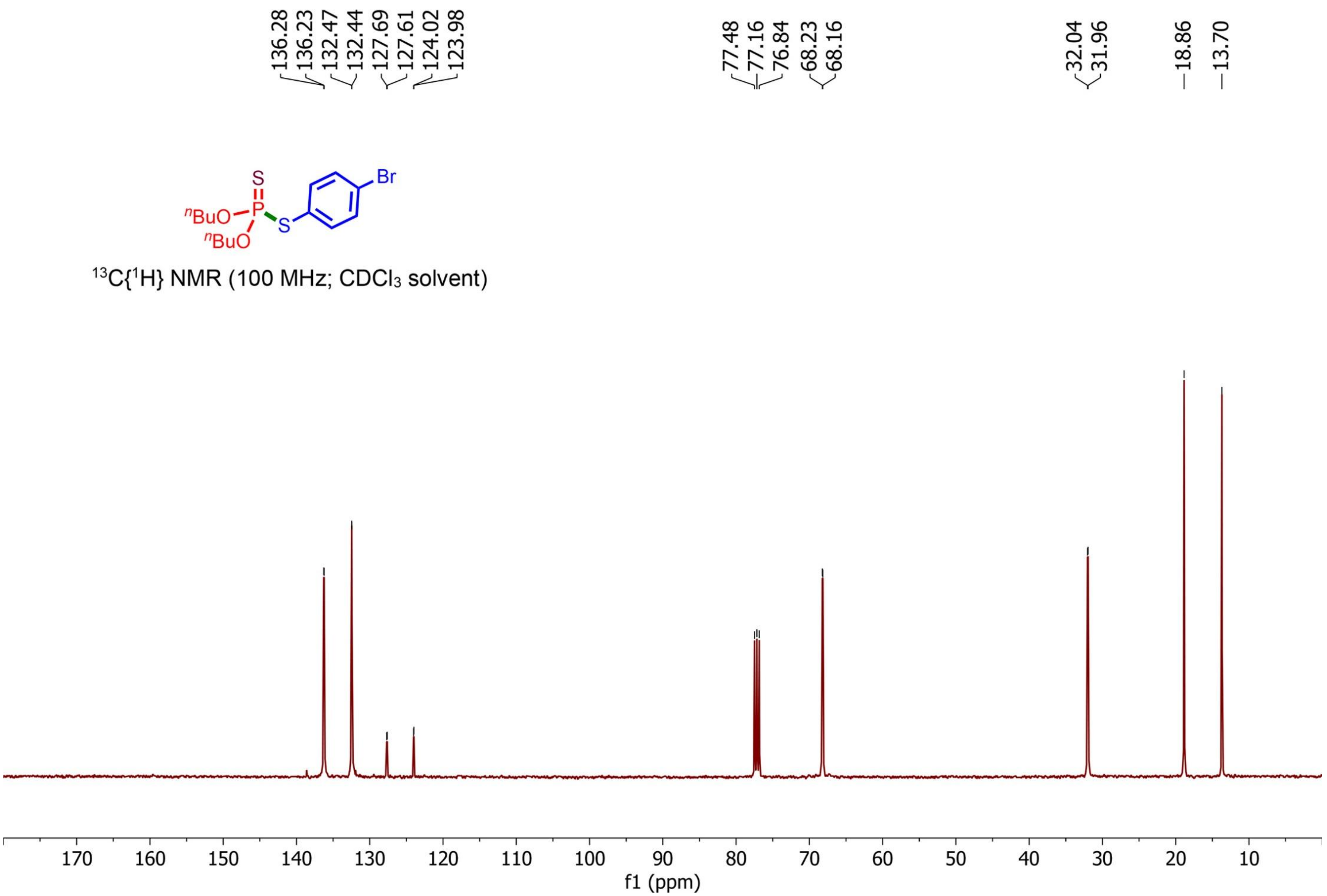

Figure S32. $^{13}C\{^{1}H\}$ NMR spectrum of *S*-(4-bromophenyl) *O*,*O*-dibutyl phosphorodithioate (**3m**)

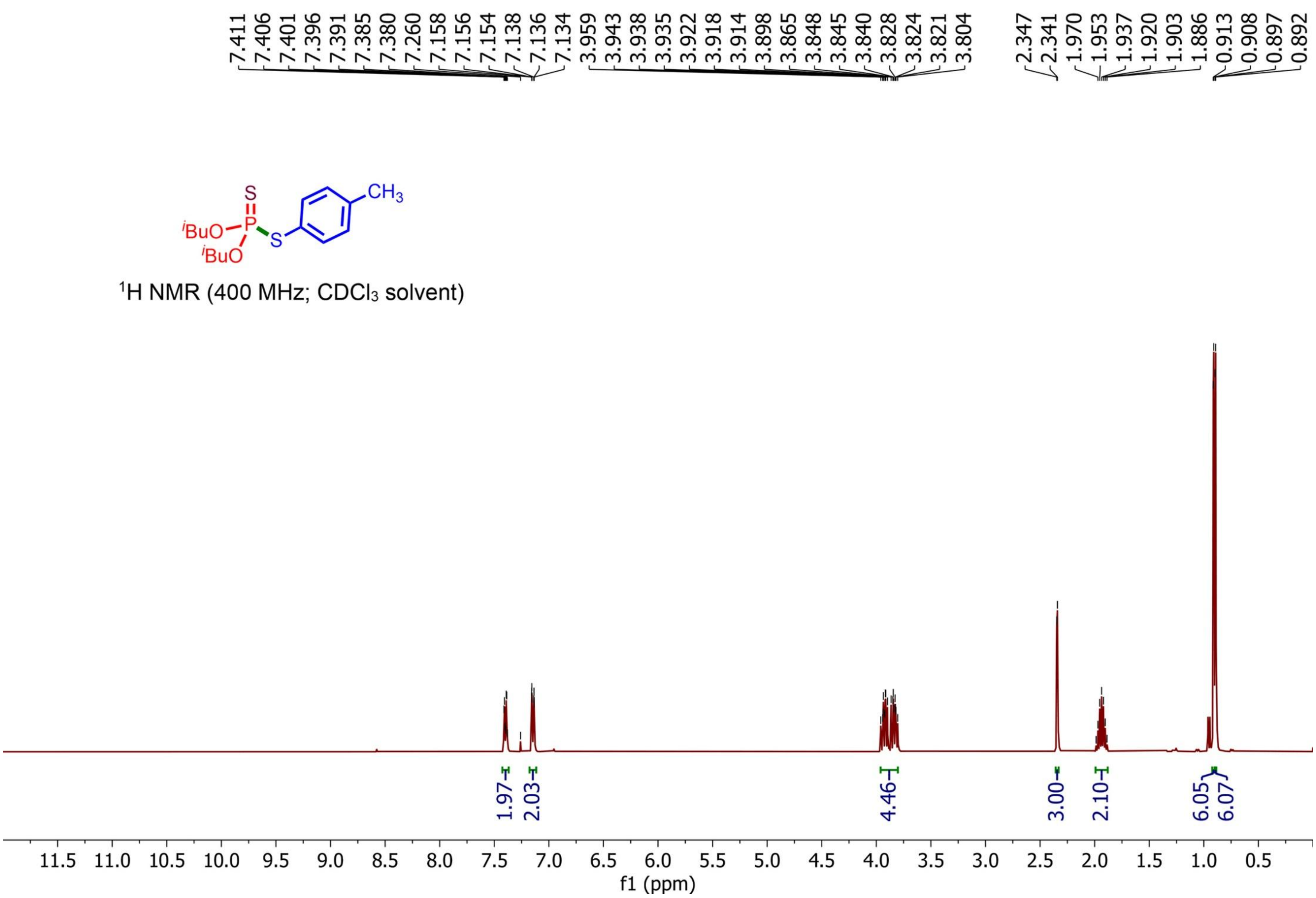

Figure S33. ^{1}H NMR spectrum of *O*,*O*-diisobutyl *S*-(*p*-tolyl) phosphorodithioate (**3n**)

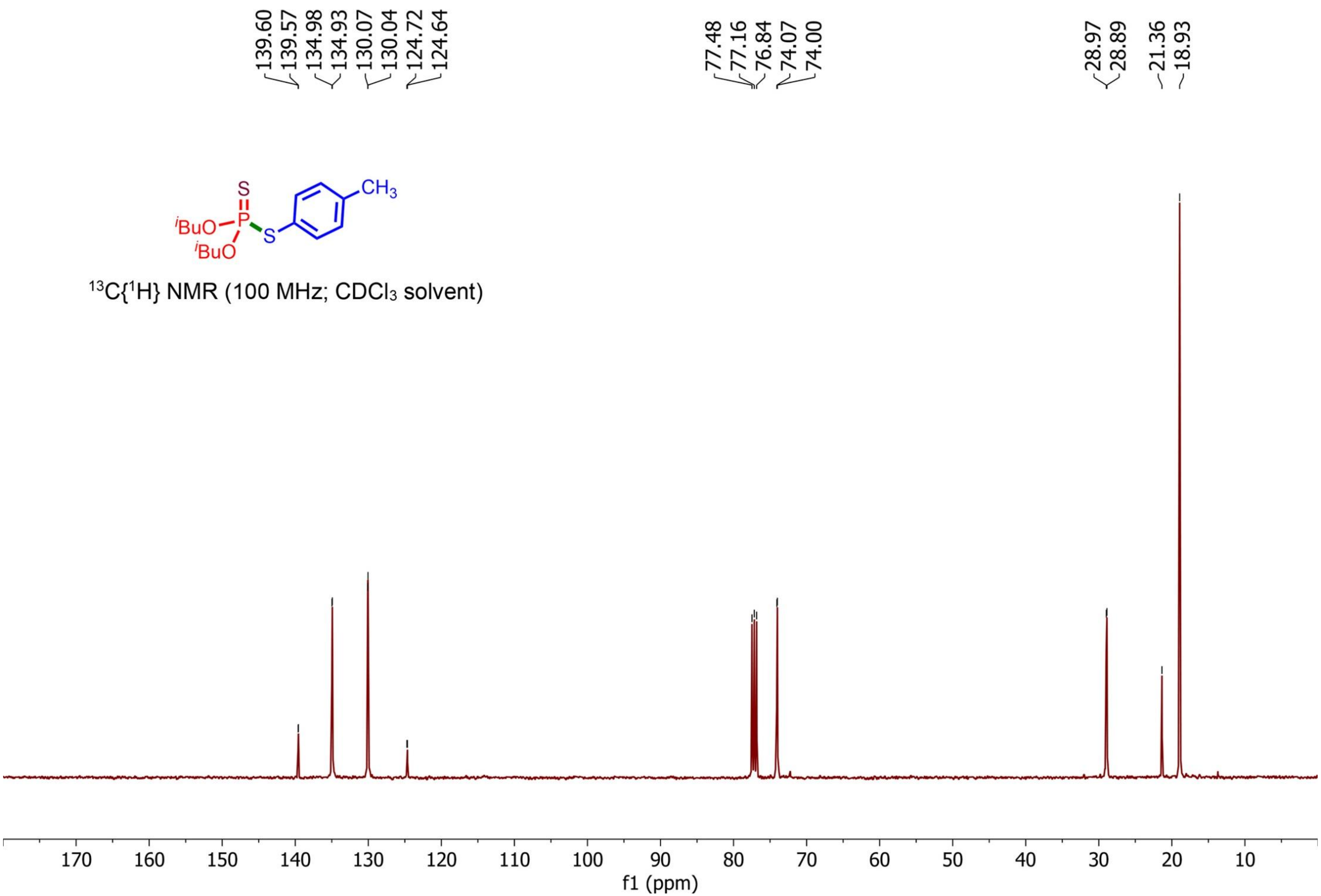

Figure S34. $^{13}C\{^1H\}$ NMR spectrum of *O*,*O*-diisobutyl *S*-(*p*-tolyl) phosphorodithioate (**3n**)

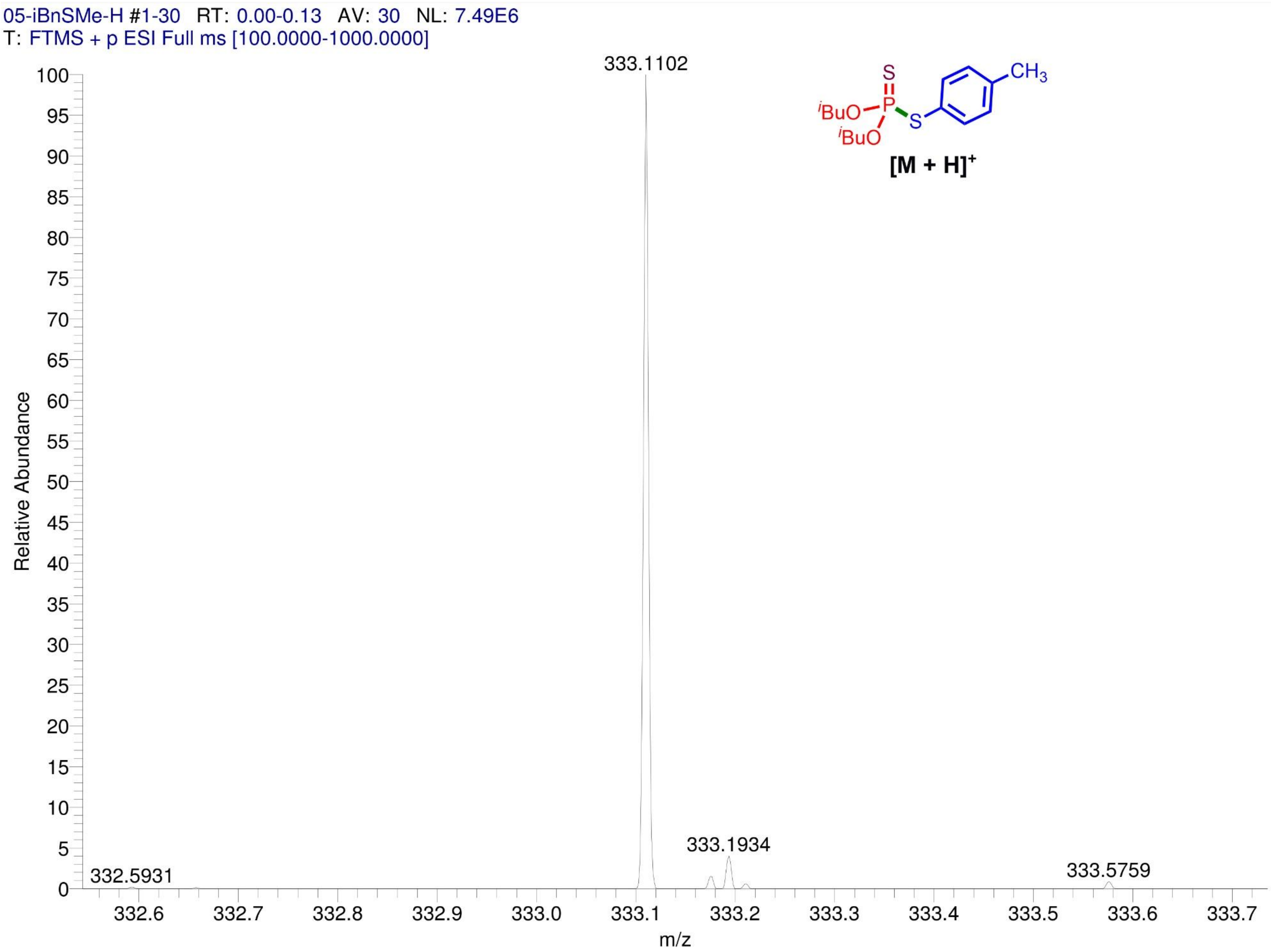

Figure S35. HRMS spectrum of *O*,*O*-diisobutyl *S*-(*p*-tolyl) phosphorodithioate (**3n**)

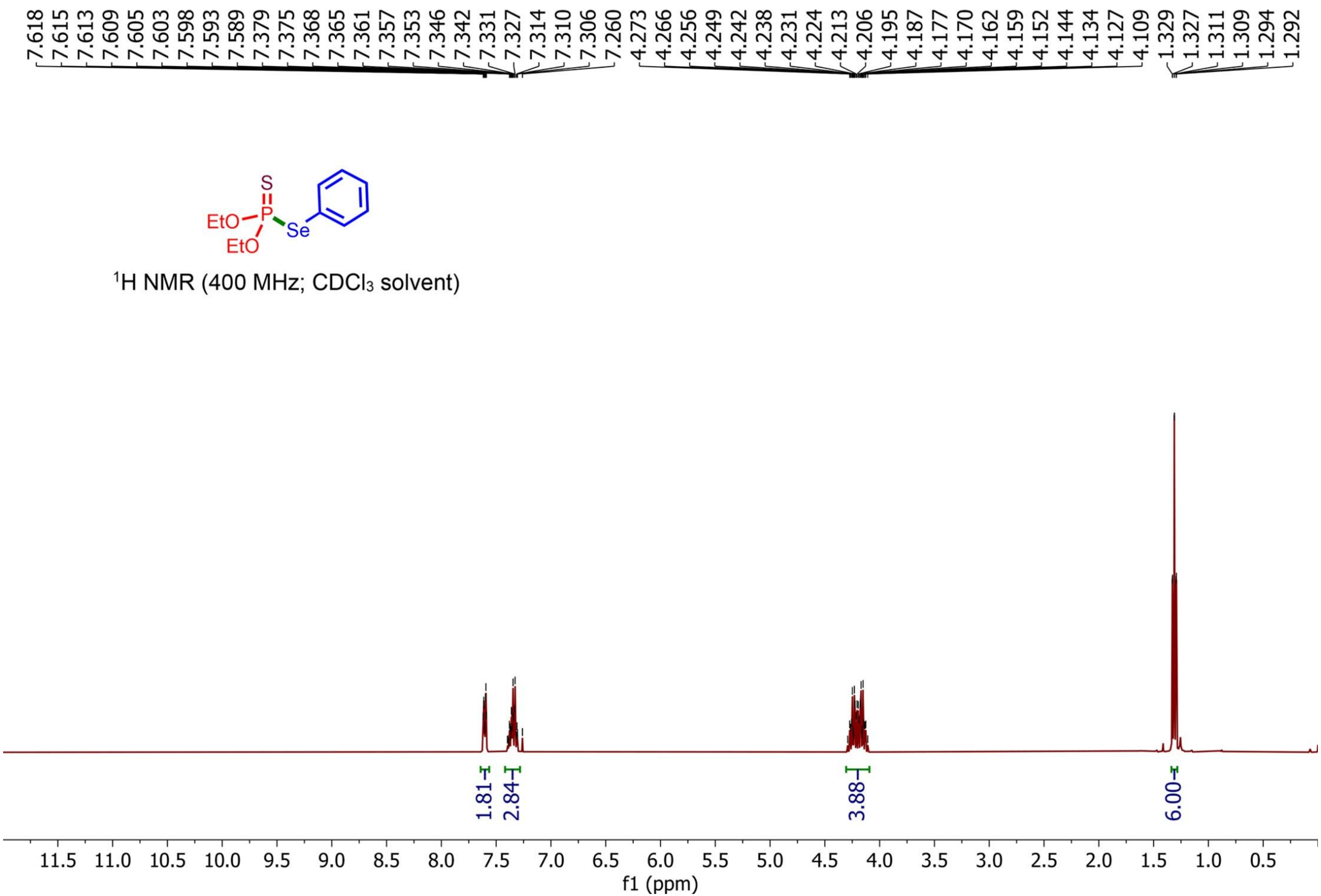

Figure S36. ^{1}H NMR spectrum of *O*,*O*-diethyl *Se*-phenyl phosphoroselenothioate (**5a**)

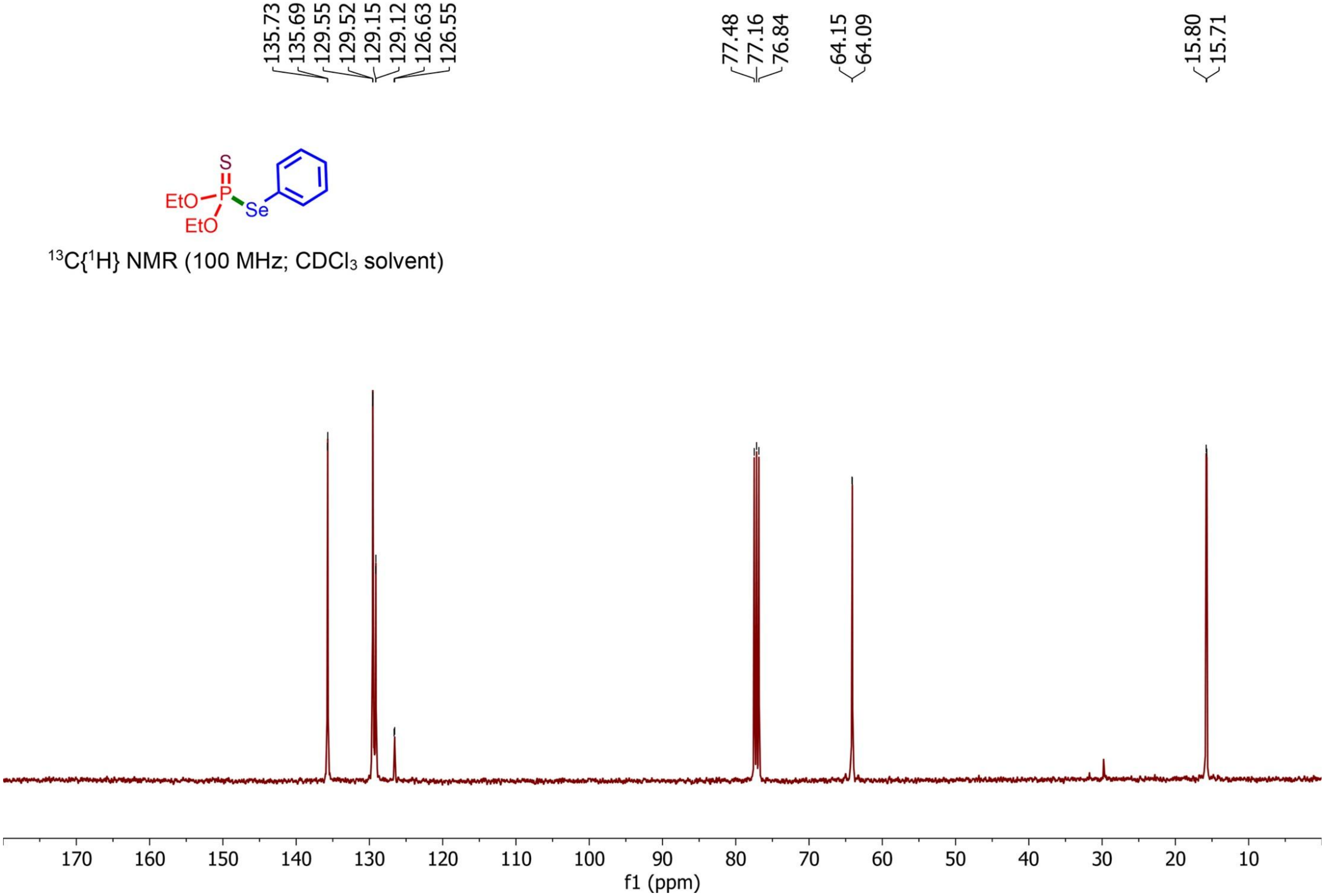

Figure S37. $^{13}C\{^{1}H\}$ NMR spectrum of *O*,*O*-diethyl *Se*-phenyl phosphoroselenothioate (**5a**)

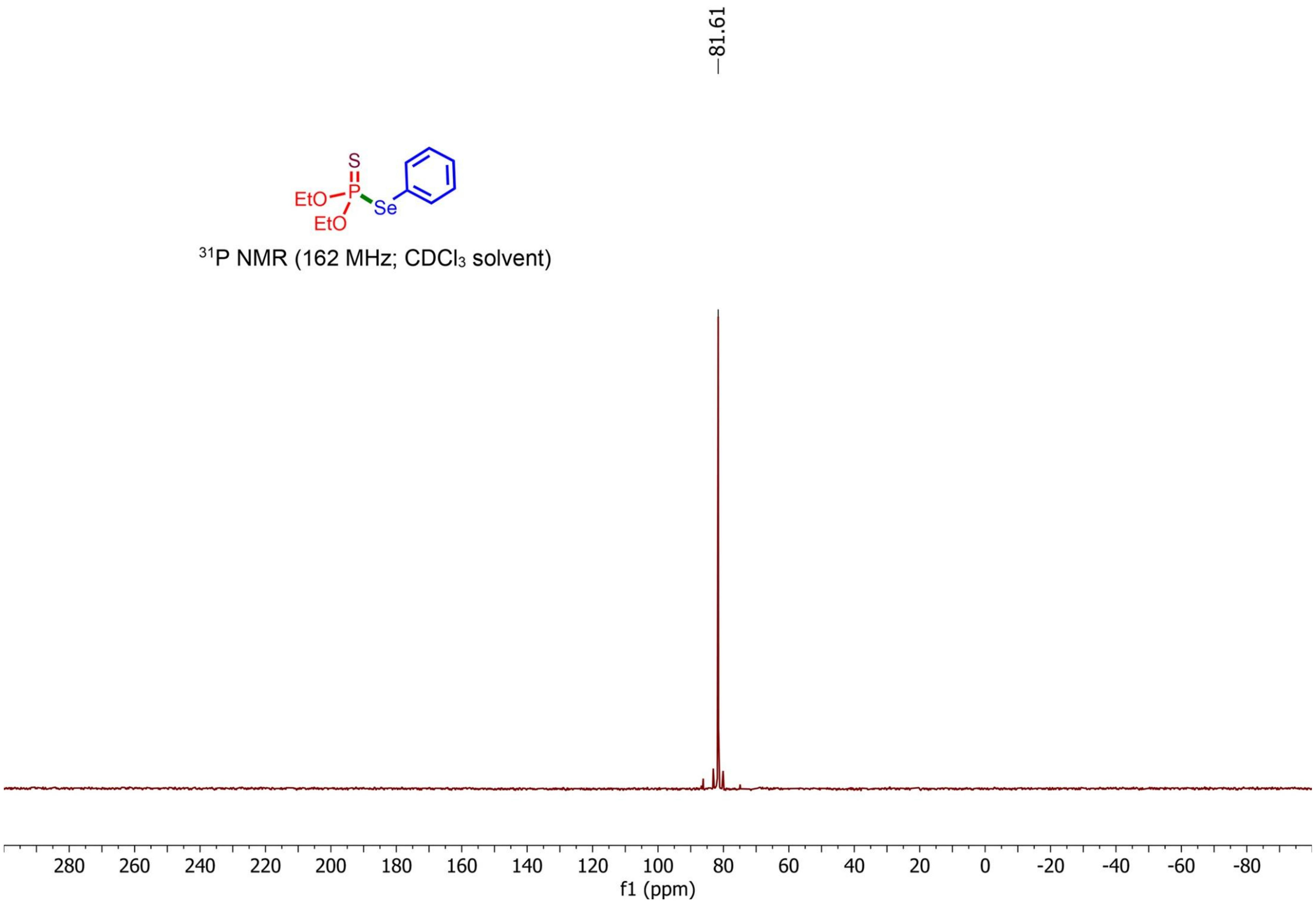

Figure S38. ^{31}P NMR spectrum of *O*,*O*-diethyl *Se*-phenyl phosphoroselenothioate (**5a**)

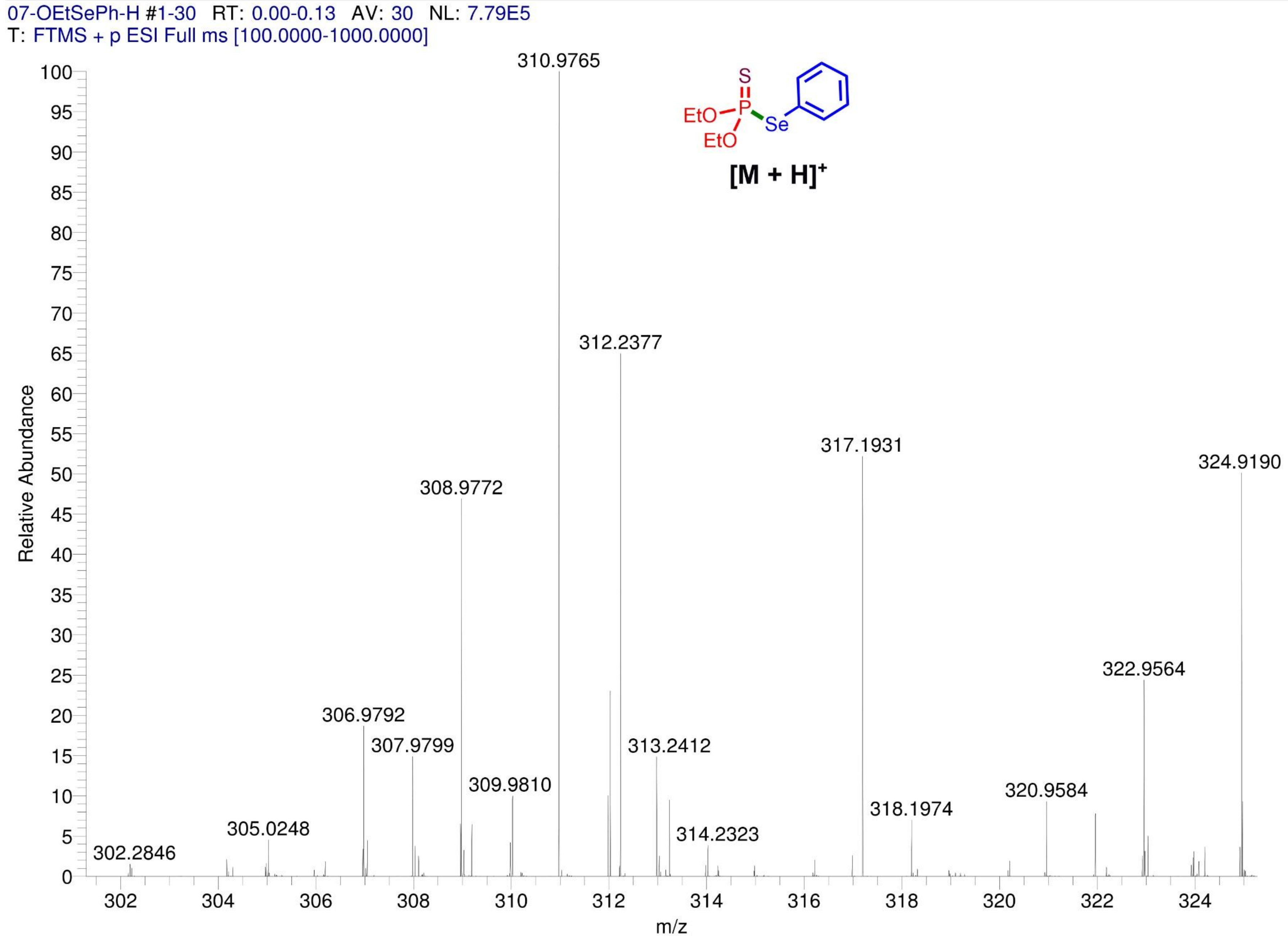

Figure S39. HRMS spectrum of *O*,*O*-diethyl *Se*-phenyl phosphoroselenothioate (**5a**)

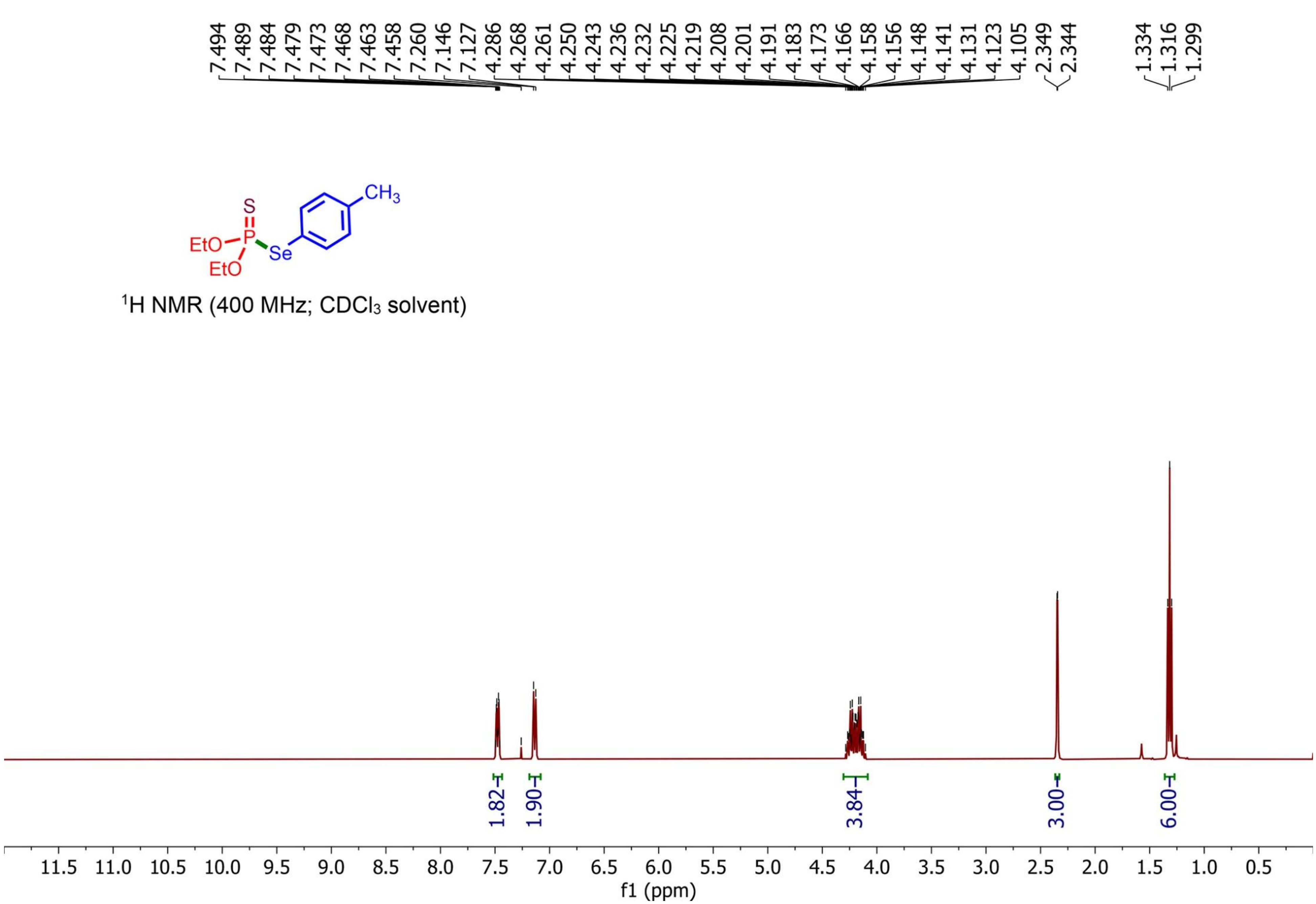

Figure S40. ^{1}H NMR spectrum of *O*,*O*-diethyl *Se*-(*p*-tolyl) phosphoroselenothioate (**5b**)

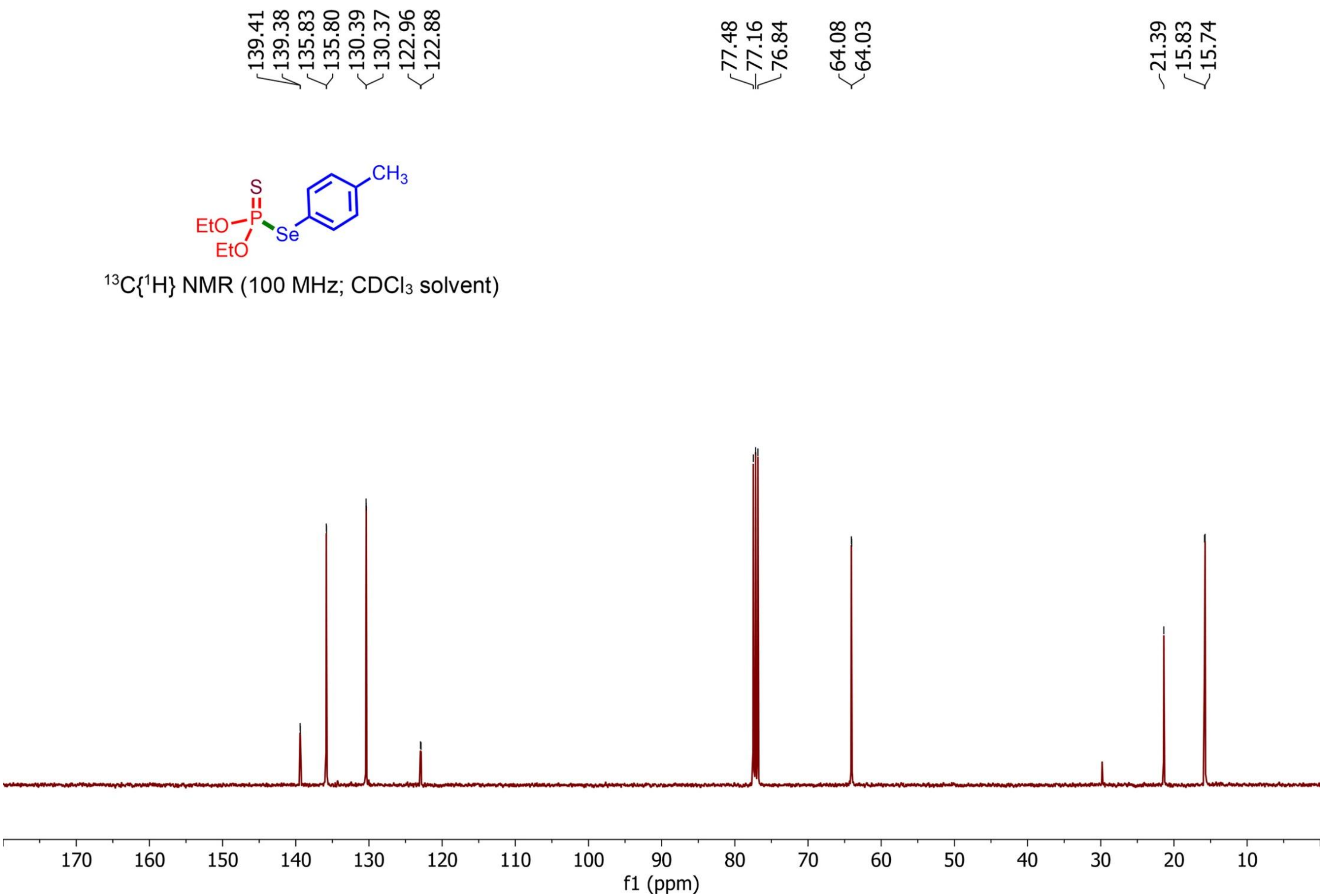

Figure S41. $^{13}C\{^1H\}$ NMR spectrum of *O*,*O*-diethyl *Se*-(*p*-tolyl) phosphoroselenothioate (**5b**)

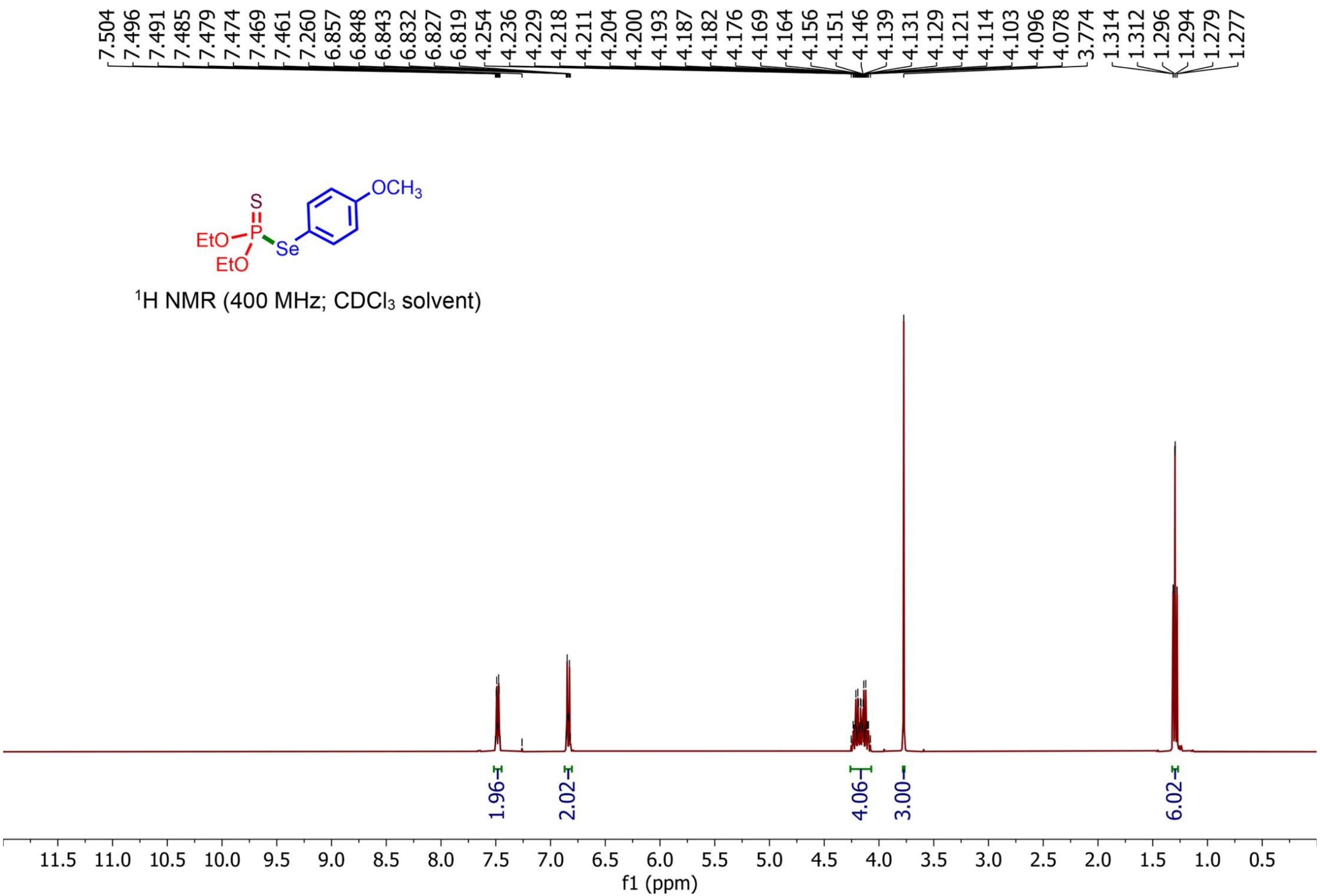

Figure S42. ^{1}H NMR spectrum of *O*,*O*-diethyl *Se*-(4-methoxyphenyl) phosphoroselenothioate (**5c**)

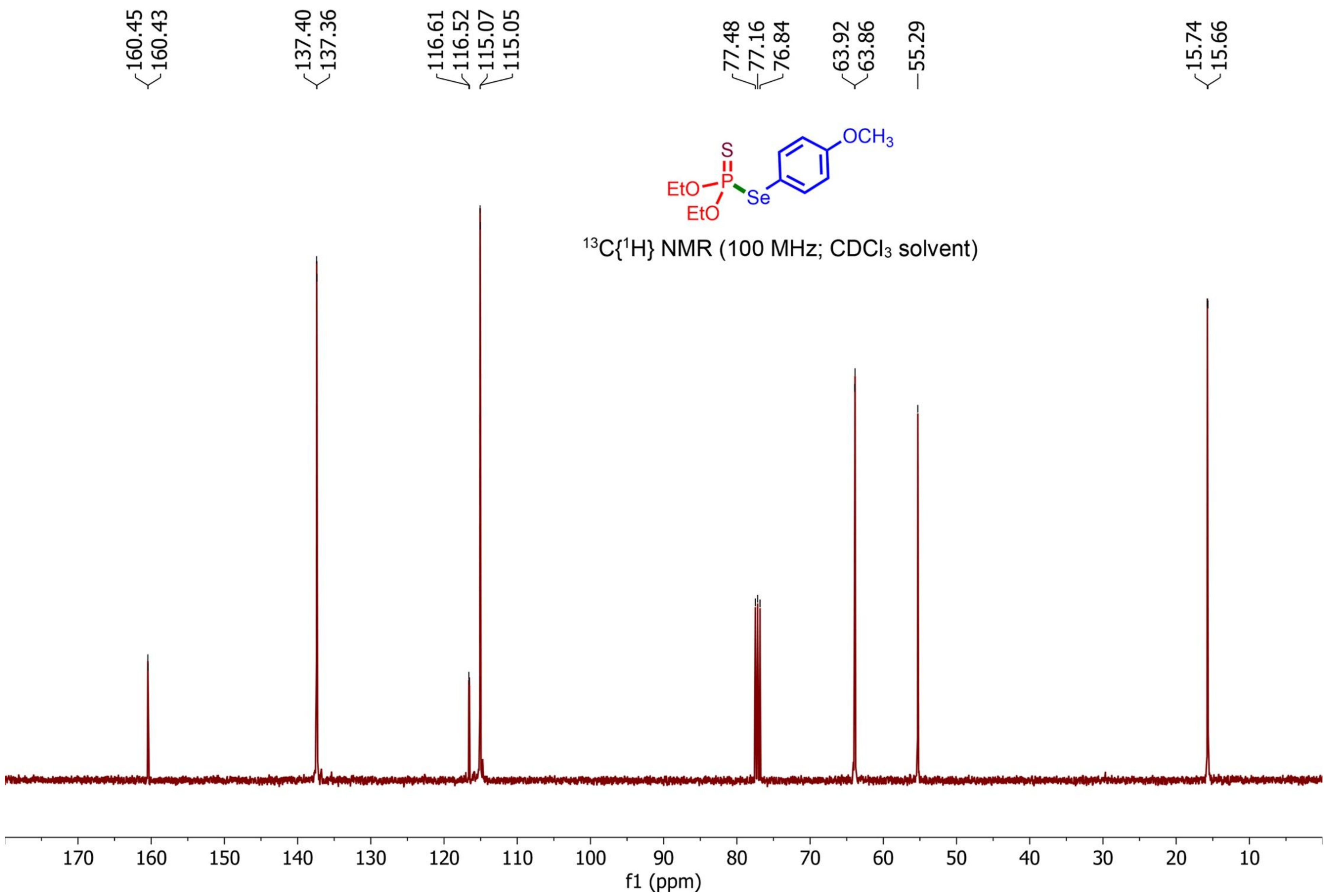

Figure S43. $^{13}C\{^{1}H\}$ NMR spectrum of *O*,*O*-diethyl *Se*-(4-methoxyphenyl) phosphoroselenothioate (**5c**)

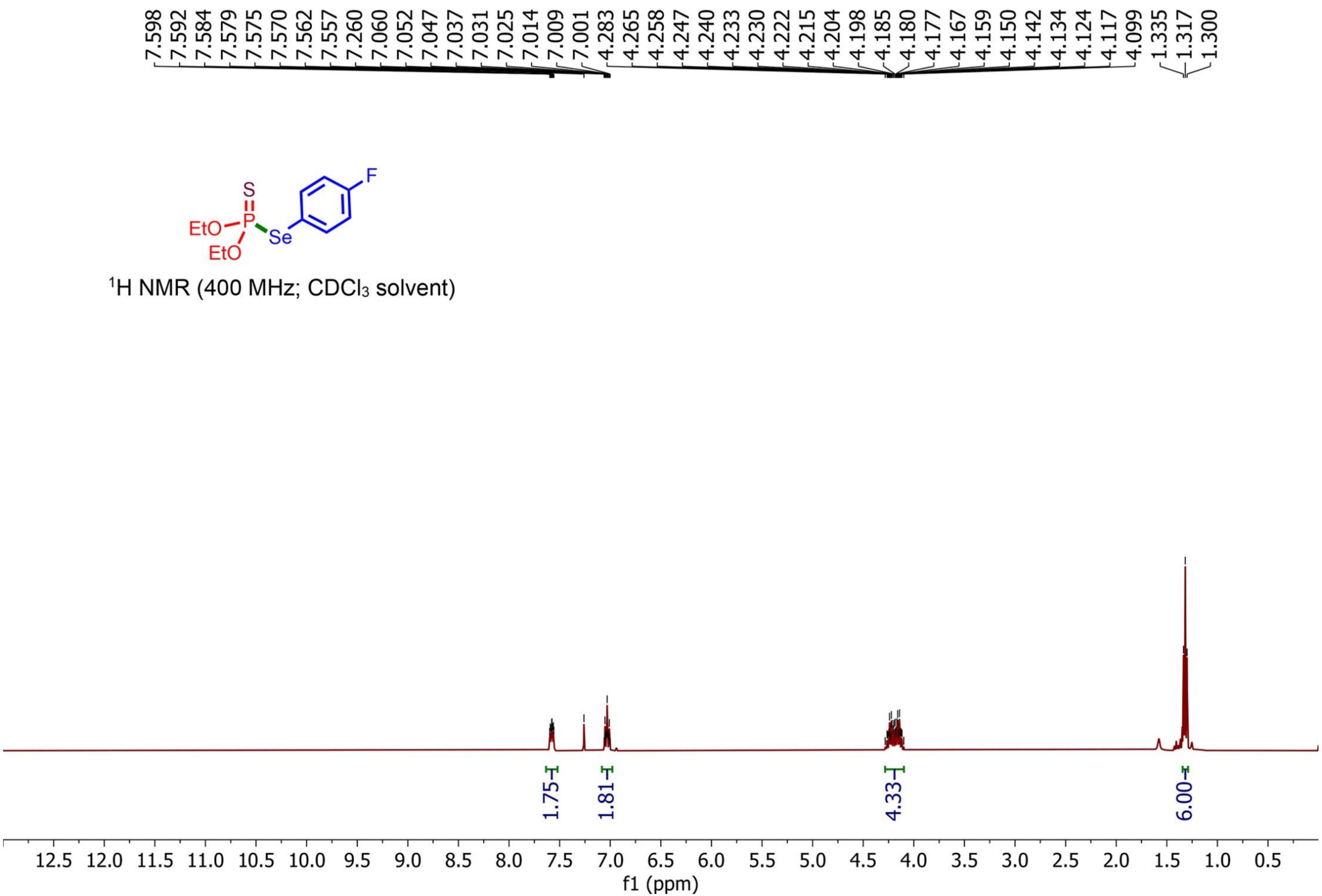

Figure S44. ^{1}H NMR spectrum of *O*,*O*-diethyl *Se*-(4-fluorophenyl) phosphoroselenothioate (**5d**)

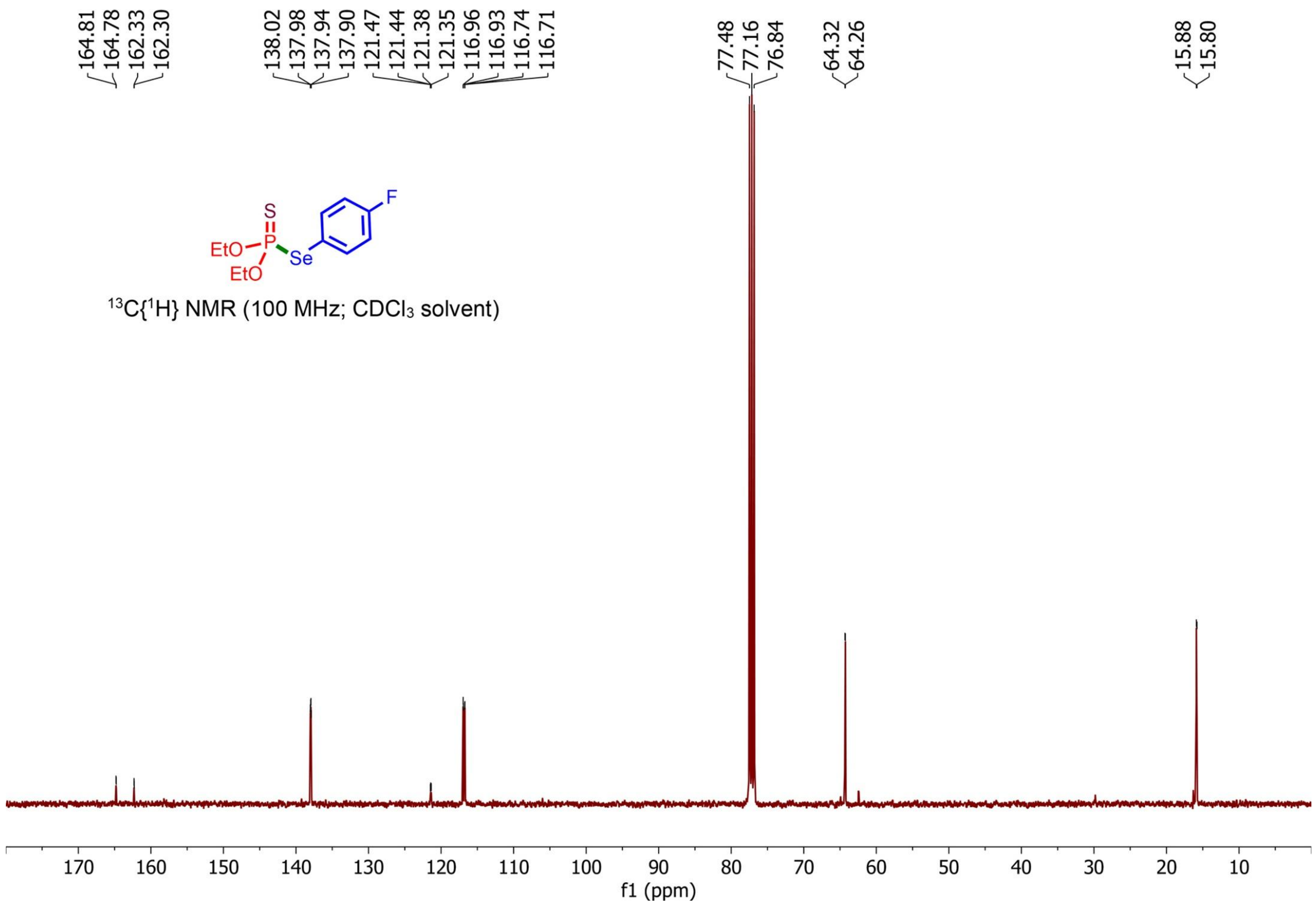

Figure S45. $^{13}C\{^{1}H\}$ NMR spectrum of *O*,*O*-diethyl *Se*-(4-fluorophenyl) phosphoroselenothioate (**5d**)

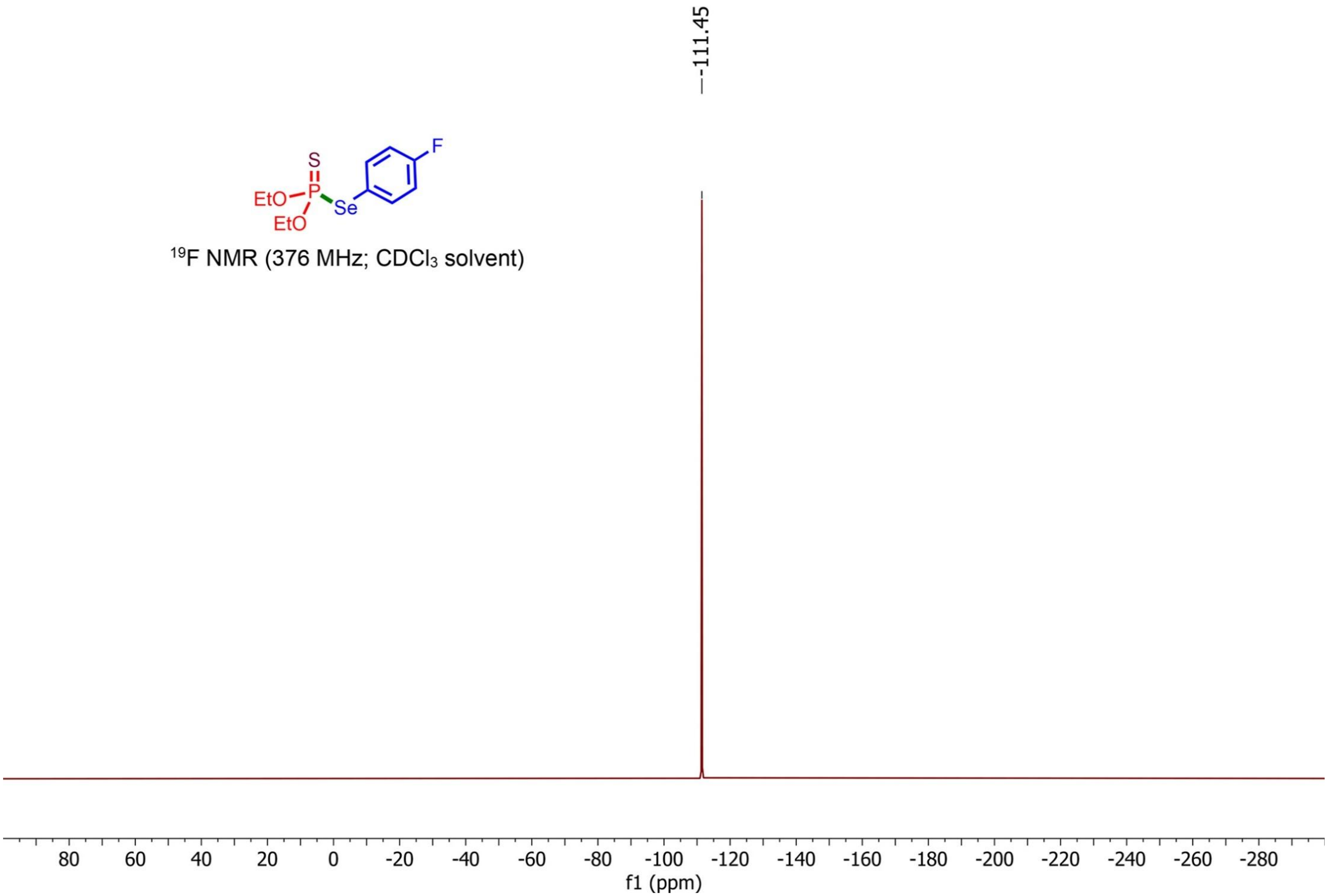

Figure S46. ^{19}F NMR spectrum of *O*,*O*-diethyl *Se*-(4-fluorophenyl) phosphoroselenothioate (**5d**)

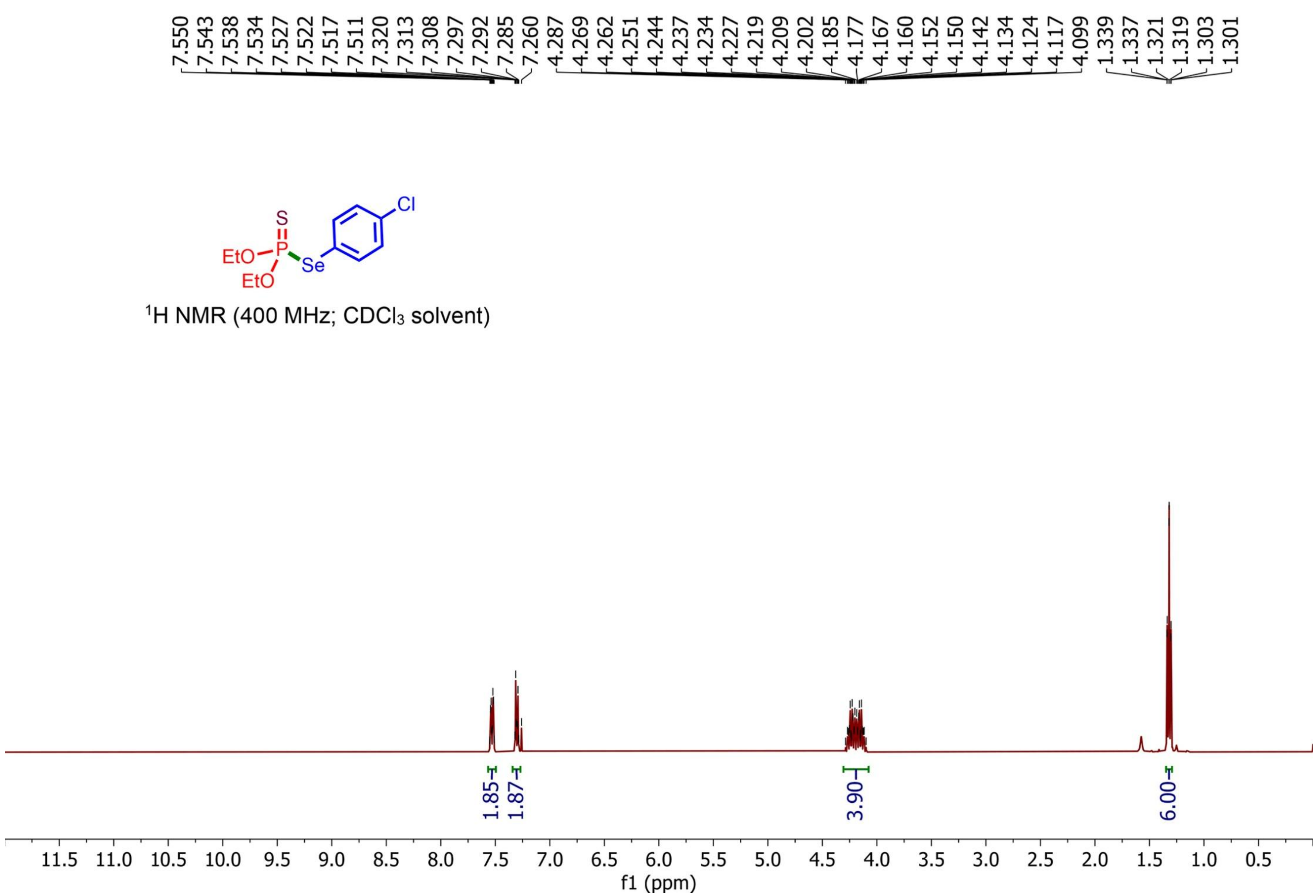

Figure S47. ^{1}H NMR spectrum of *Se*-(4-chlorophenyl) *O*,*O*-diethyl phosphoroselenothioate (**5e**)

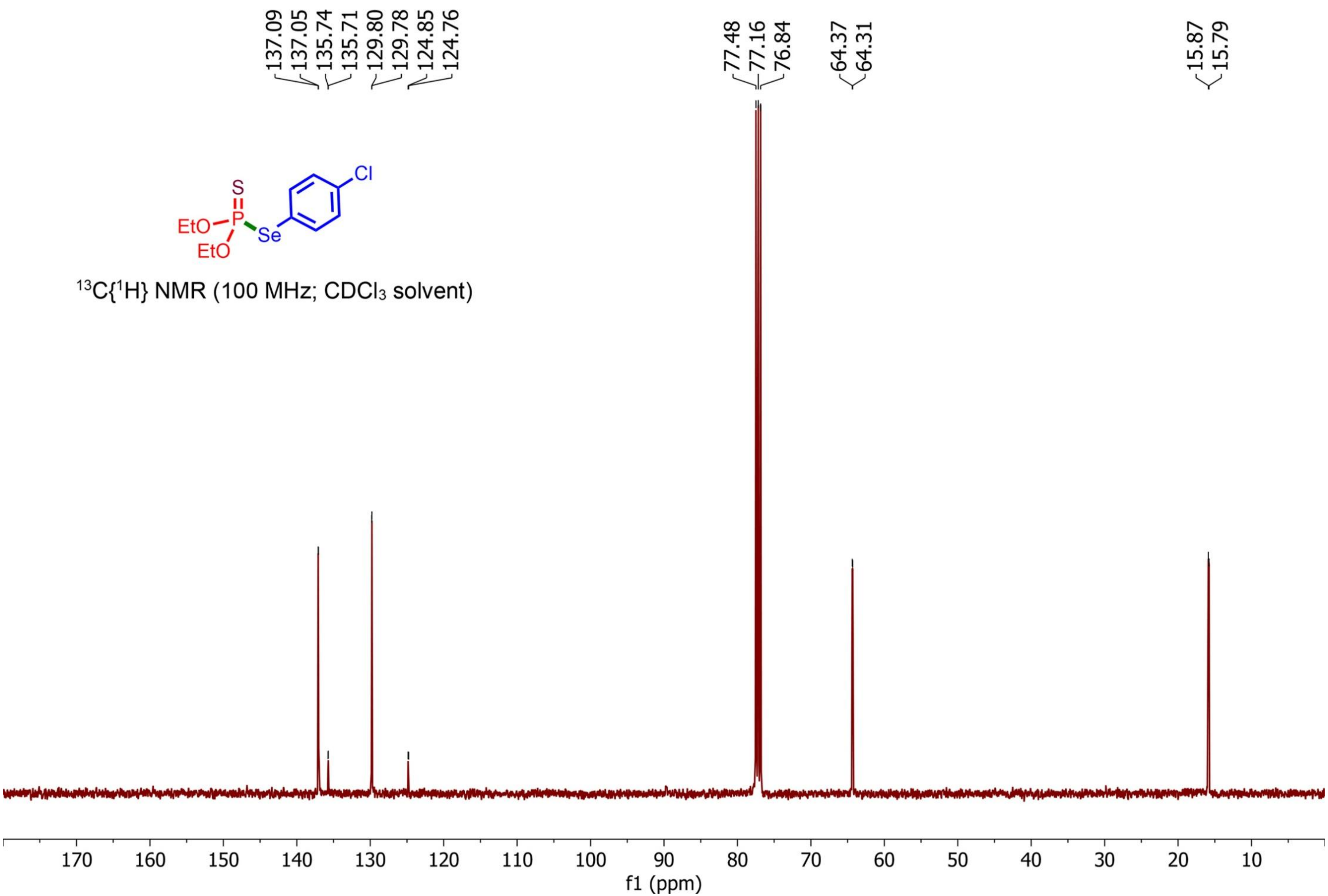

Figure S48. $^{13}C\{^1H\}$ NMR spectrum of *Se*-(4-chlorophenyl) *O*,*O*-diethyl phosphoroselenothioate (**5e**)

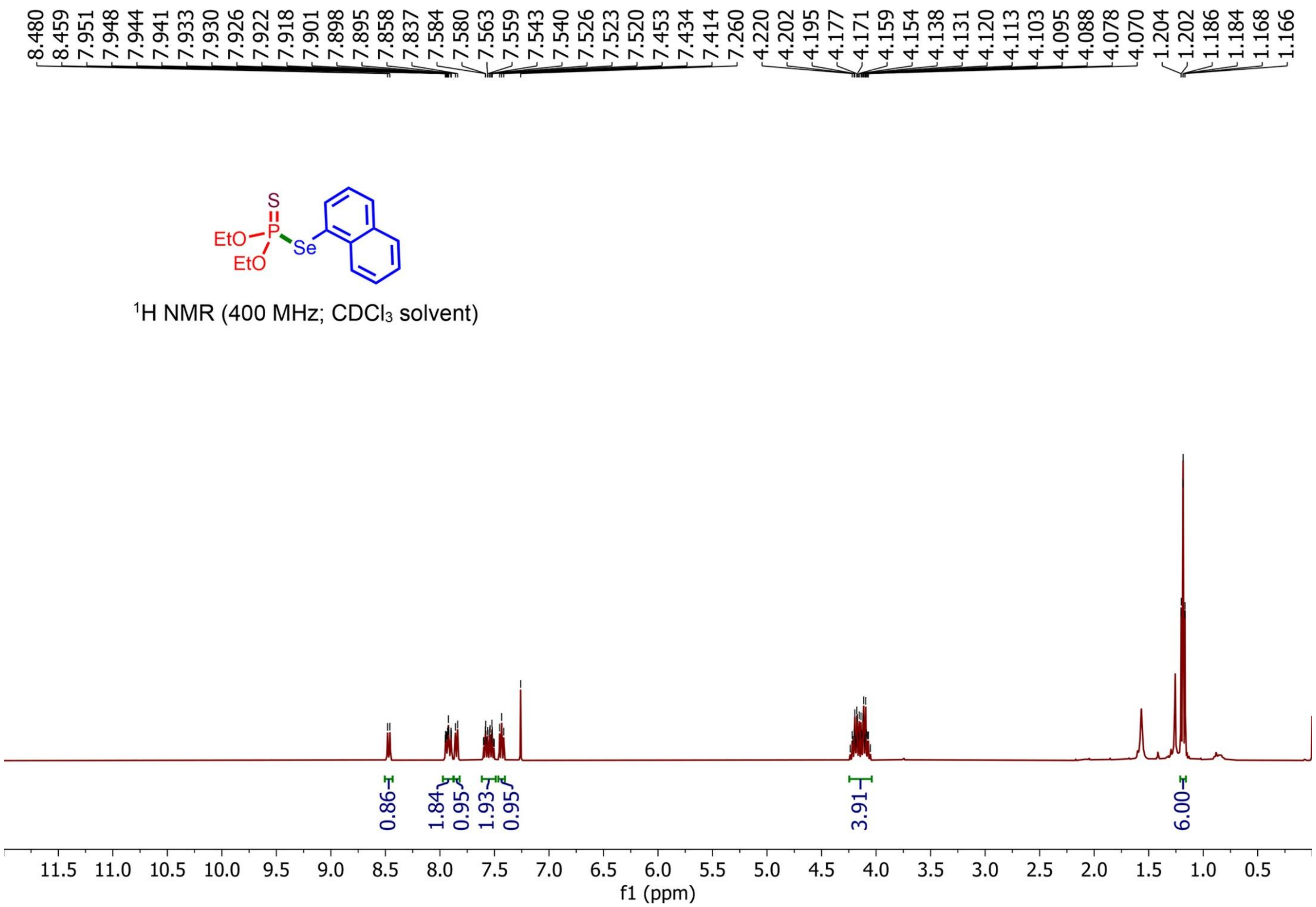

Figure S49. ^{1}H NMR spectrum of *O*,*O*-diethyl *Se*-(naphthalen-1-yl) phosphoroselenothioate (**5f**)

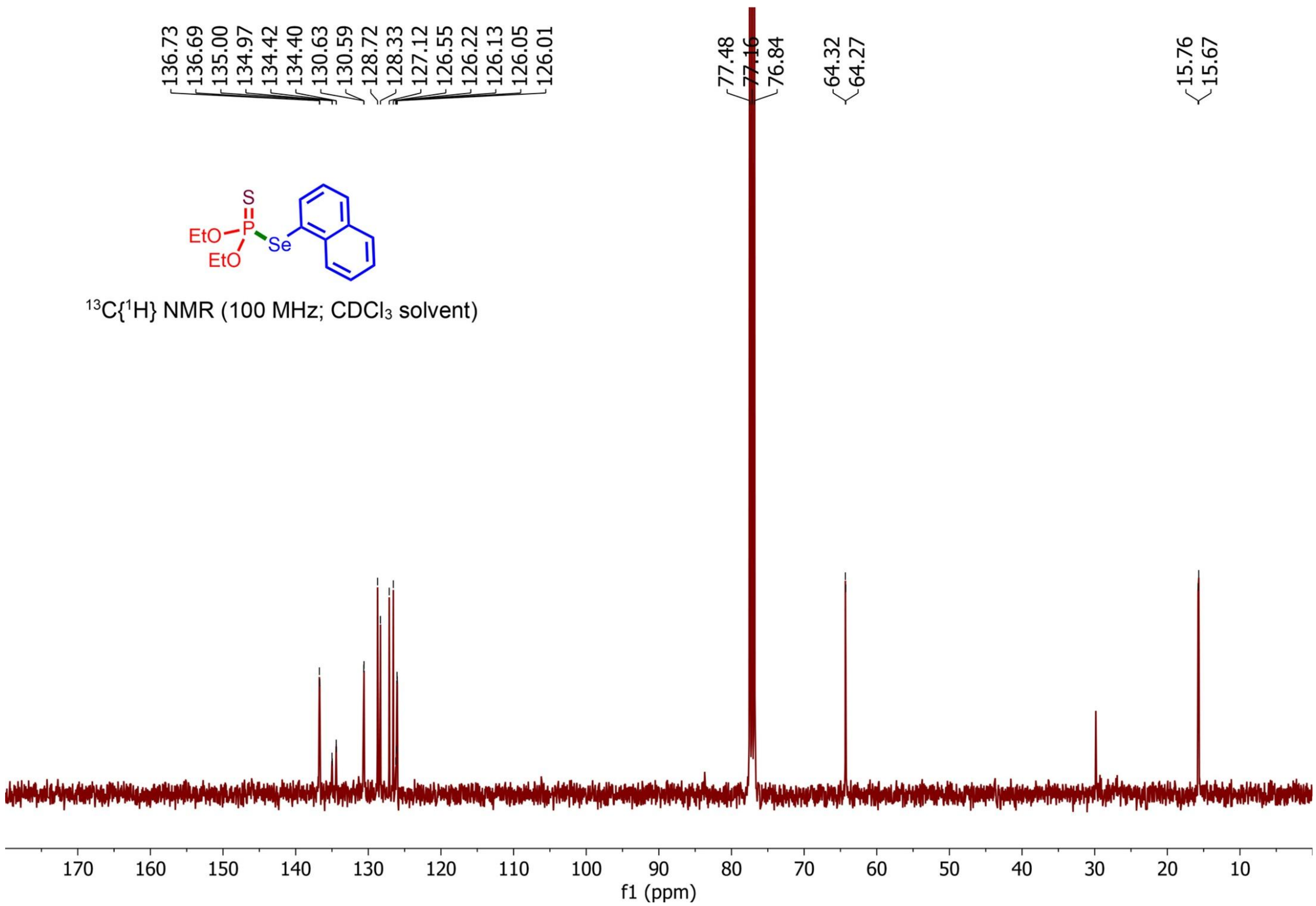

Figure S50. $^{13}C\{^{1}H\}$ NMR spectrum of *O*,*O*-diethyl *Se*-(naphthalen-1-yl) phosphoroselenothioate (**5f**)

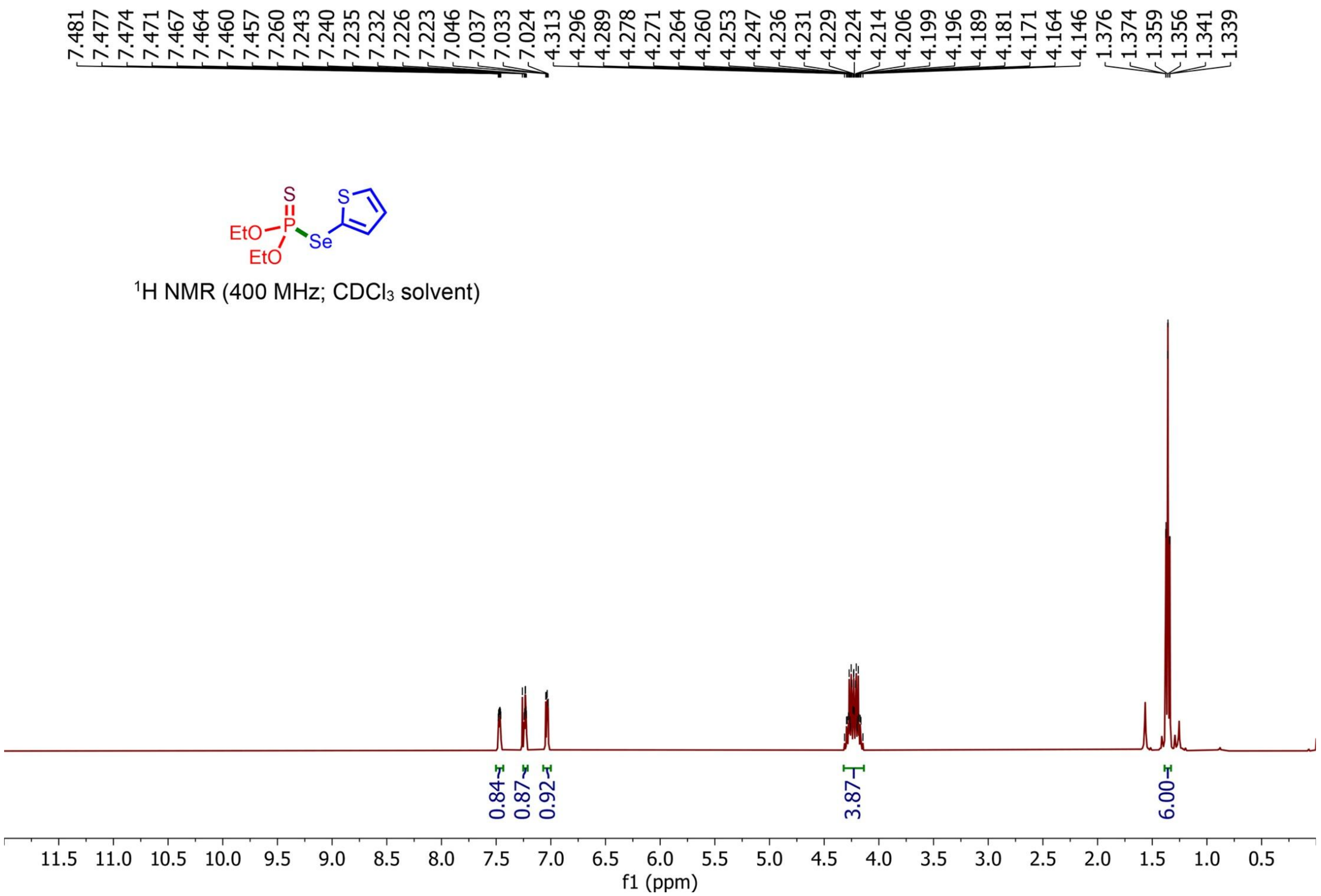

Figure S51. ^{1}H NMR spectrum of *O*,*O*-diethyl *Se*-(thiophen-2-yl) phosphoroselenothioate (**5g**)

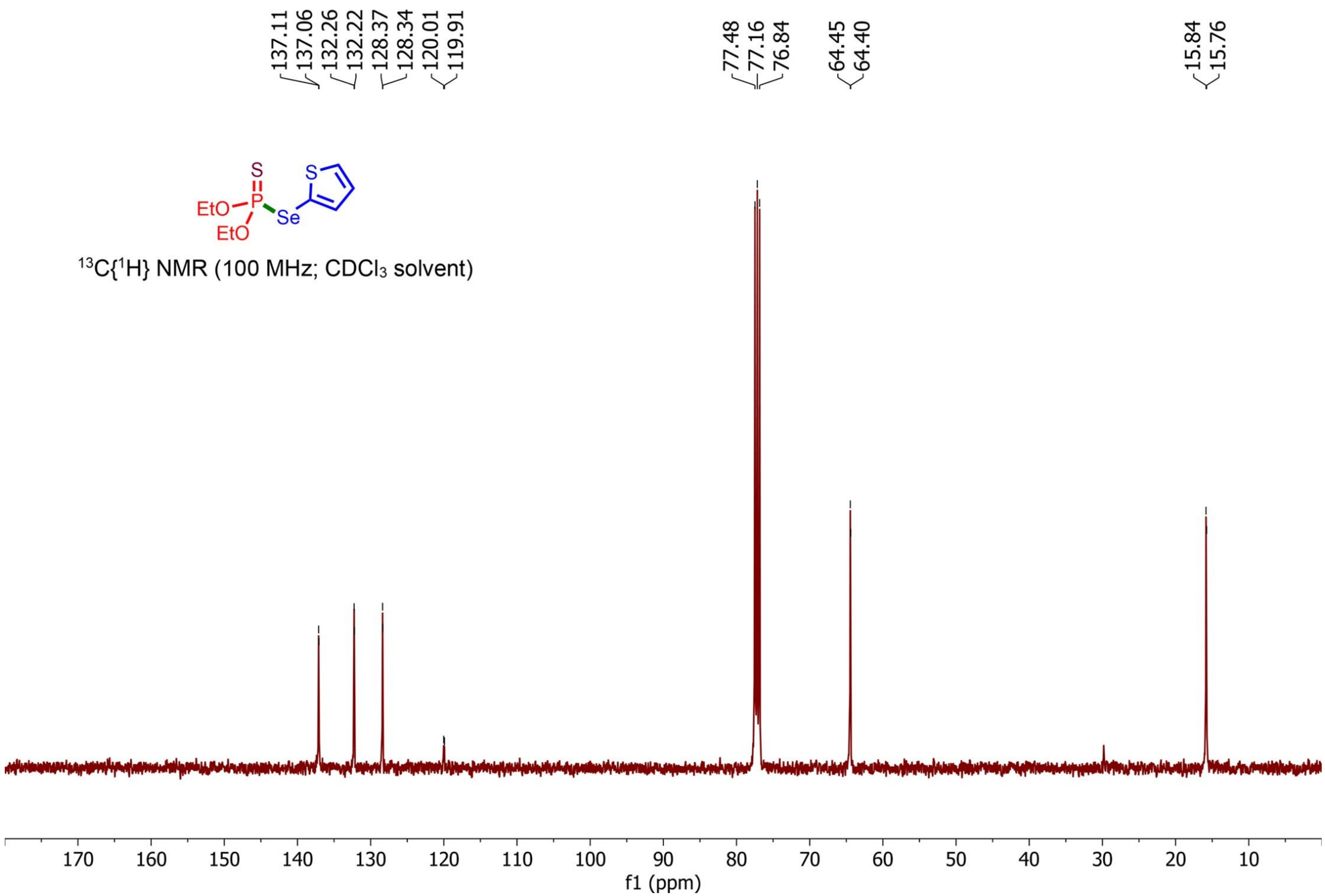

Figure S52. $^{13}C\{^1H\}$ NMR spectrum of *O*,*O*-diethyl *Se*-(thiophen-2-yl) phosphoroselenothioate (**5g**)

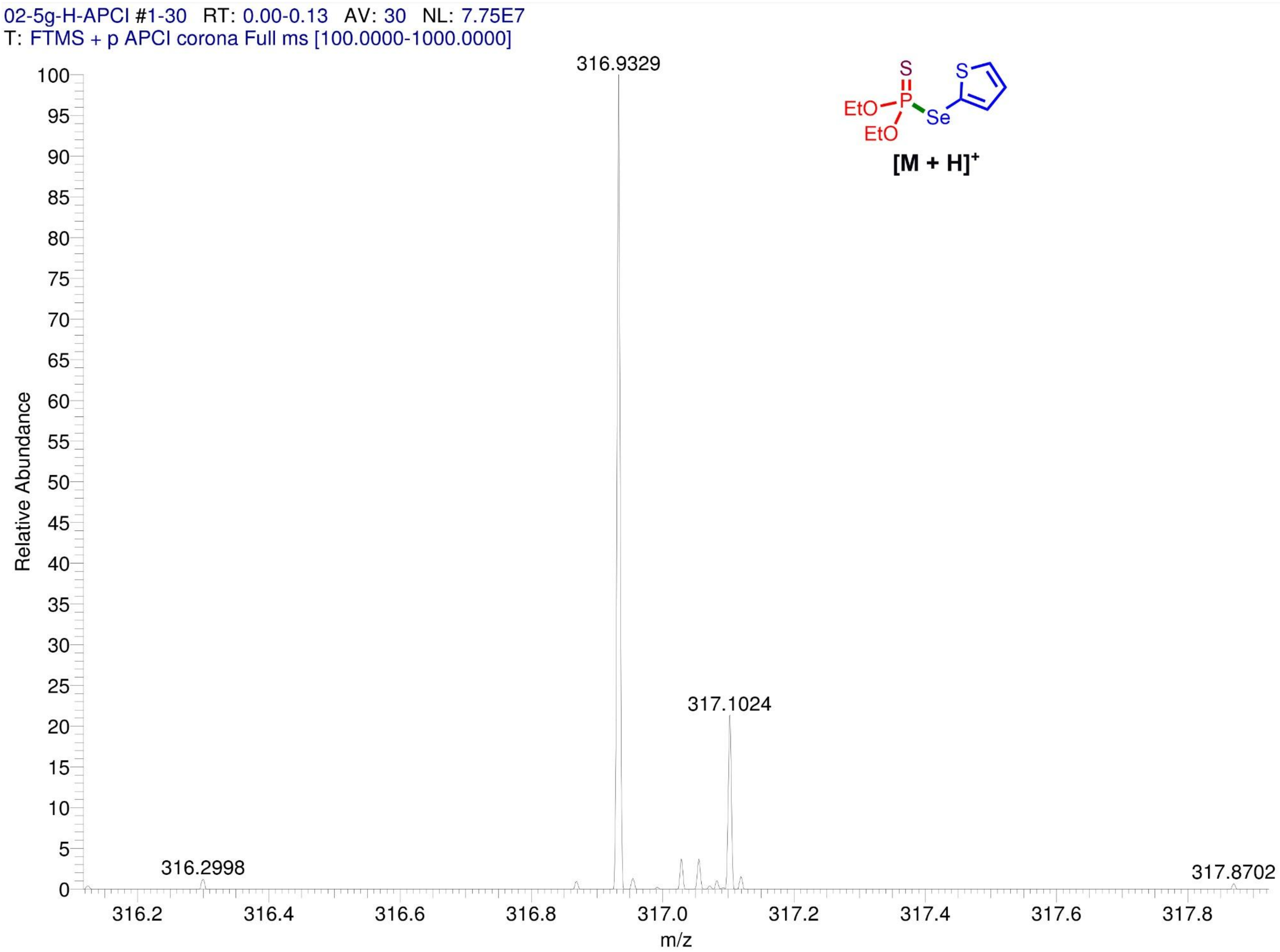

Figure S53. $^{13}C\{^{1}H\}$ NMR spectrum of *O*,*O*-diethyl *Se*-(thiophen-2-yl) phosphoroselenothioate (**5g**)

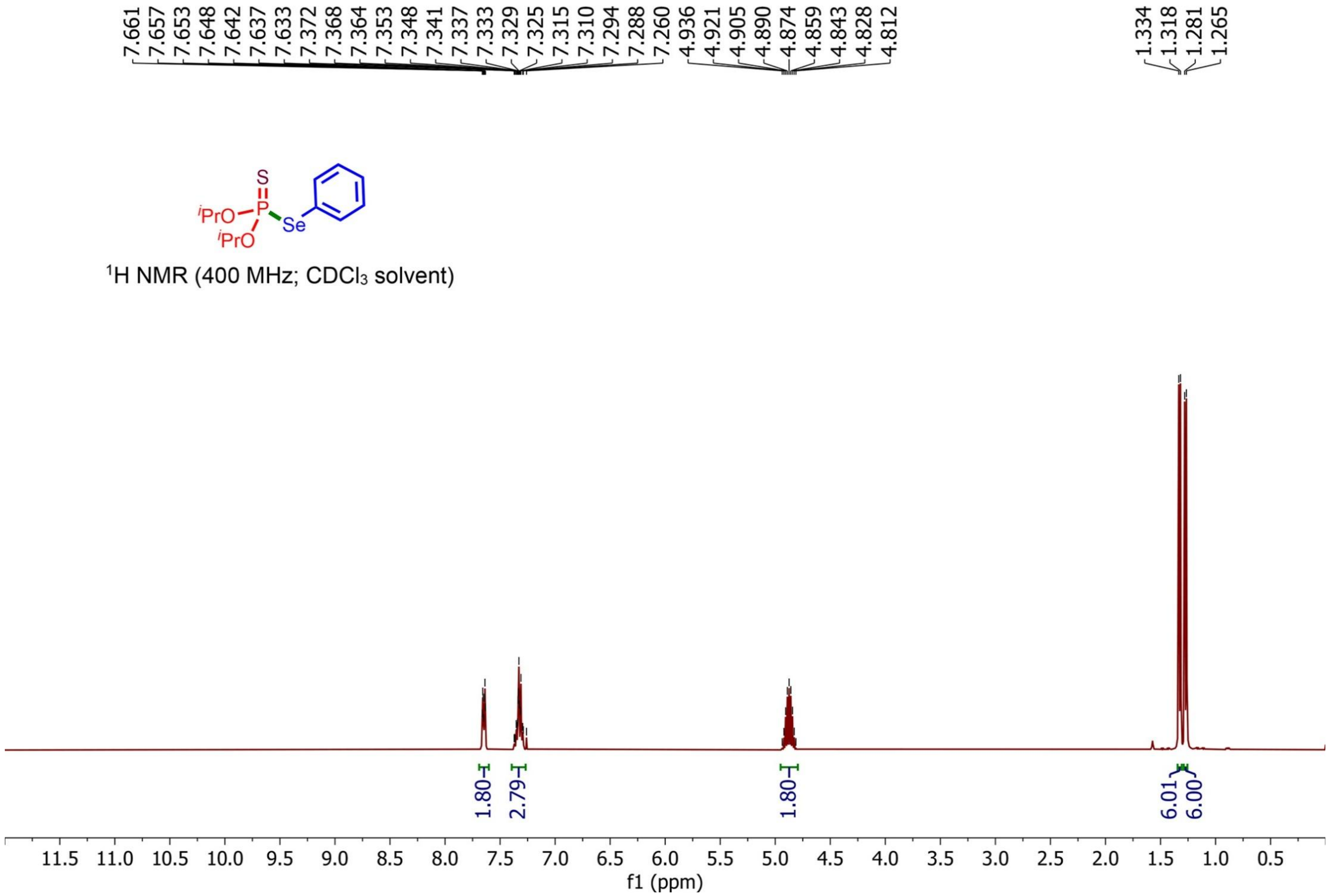

Figure S54. ^{1}H NMR spectrum of *O*,*O*-diisopropyl *Se*-phenyl phosphoroselenothioate (**5h**)

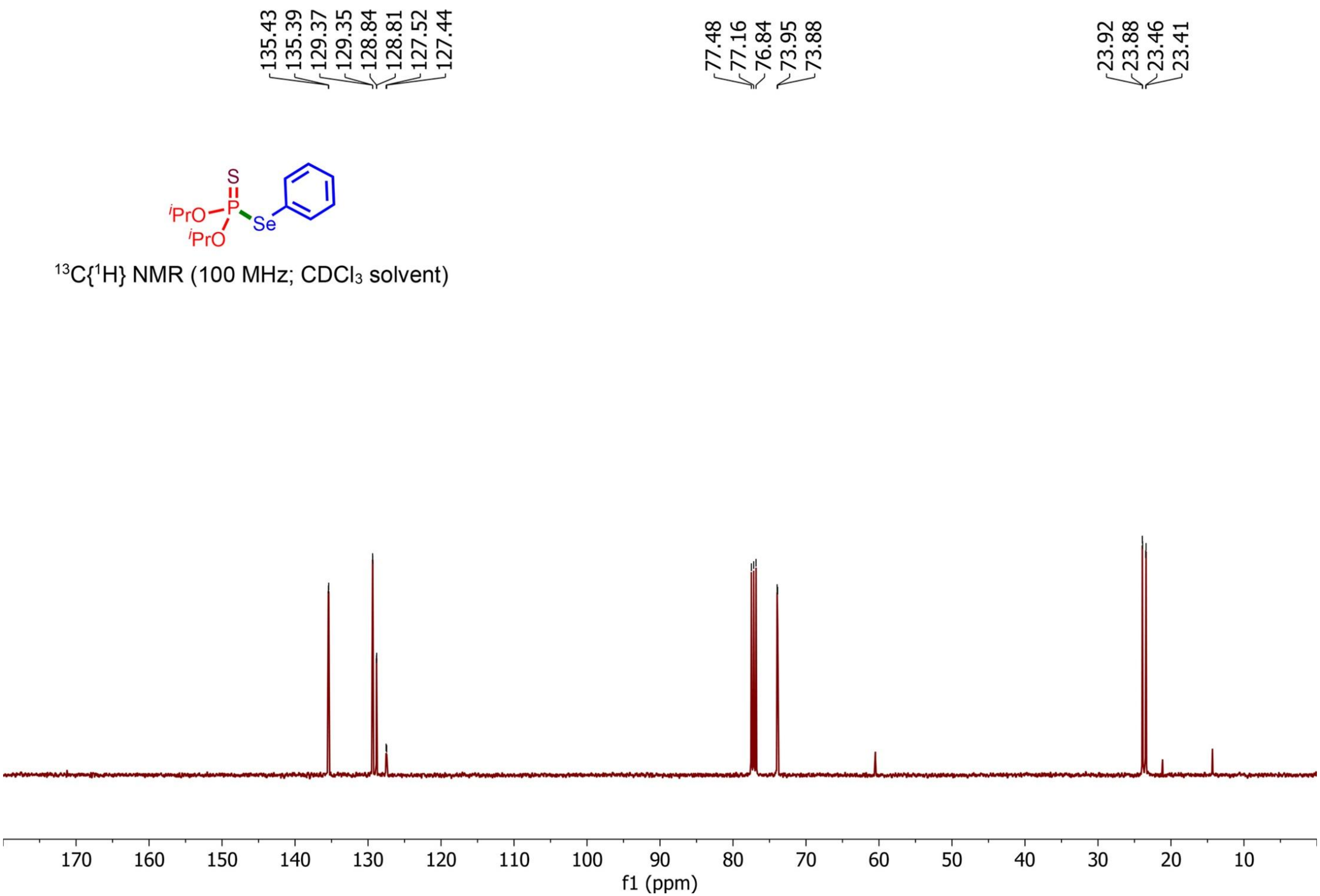

Figure S55. $^{13}C\{^1H\}$ NMR spectrum of *O*,*O*-diisopropyl *Se*-phenyl phosphoroselenothioate (**5h**)

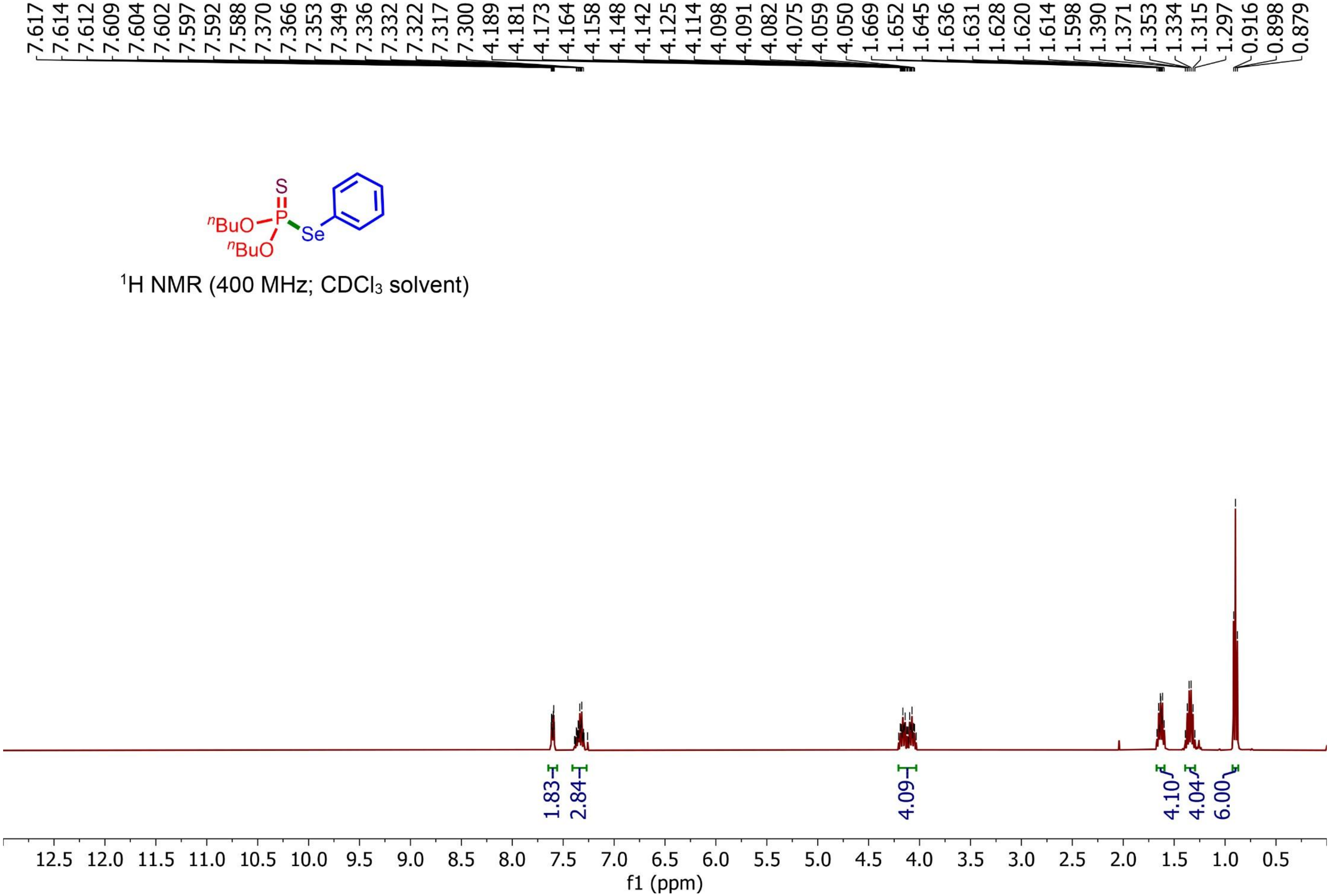

Figure S56. ^{1}H NMR spectrum of *O*,*O*-dibutyl *Se*-phenyl phosphoroselenothioate (**5i**)

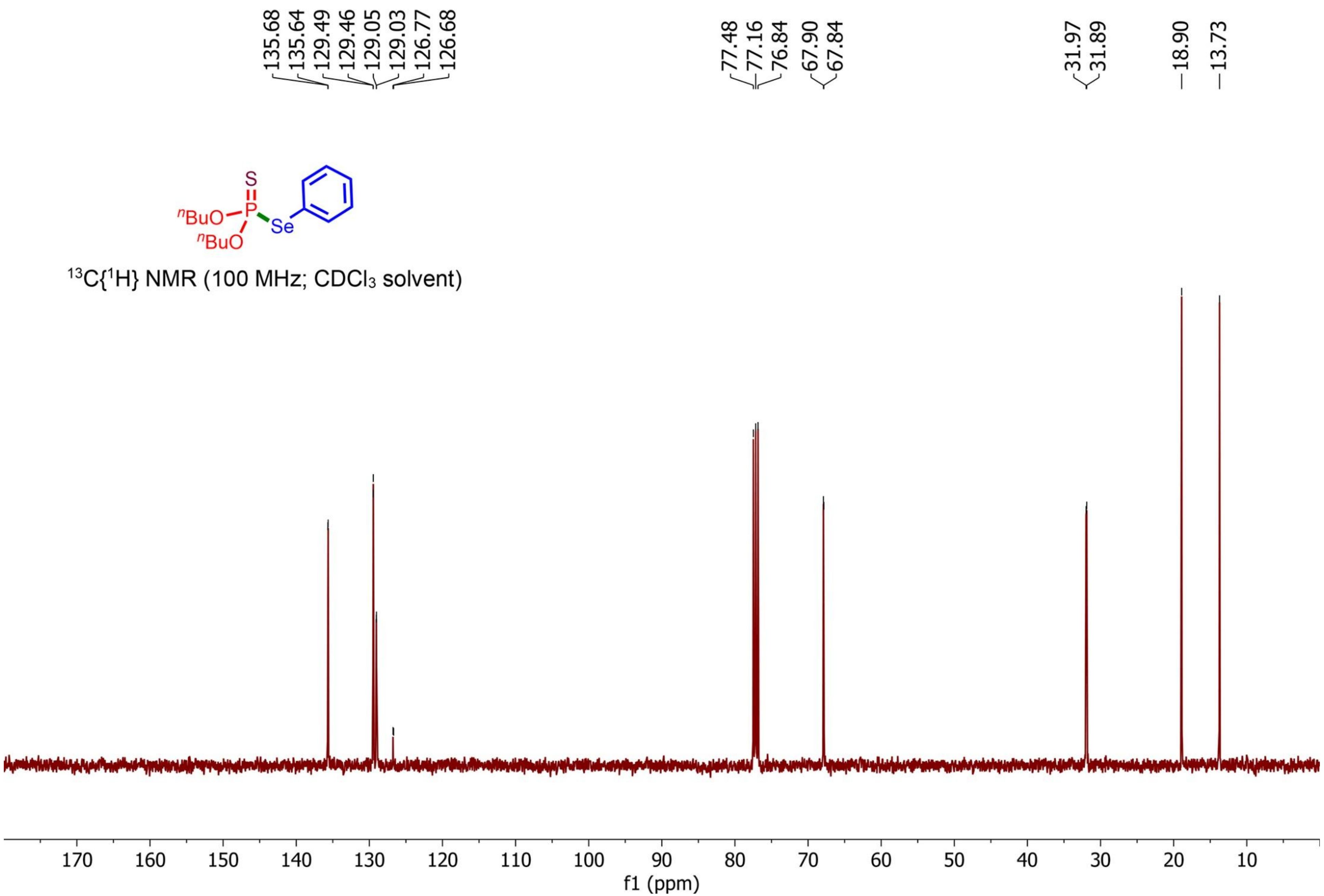

Figure S57. $^{13}C\{^{1}H\}$ NMR spectrum of *O*,*O*-dibutyl *Se*-phenyl phosphoroselenothioate (**5i**)

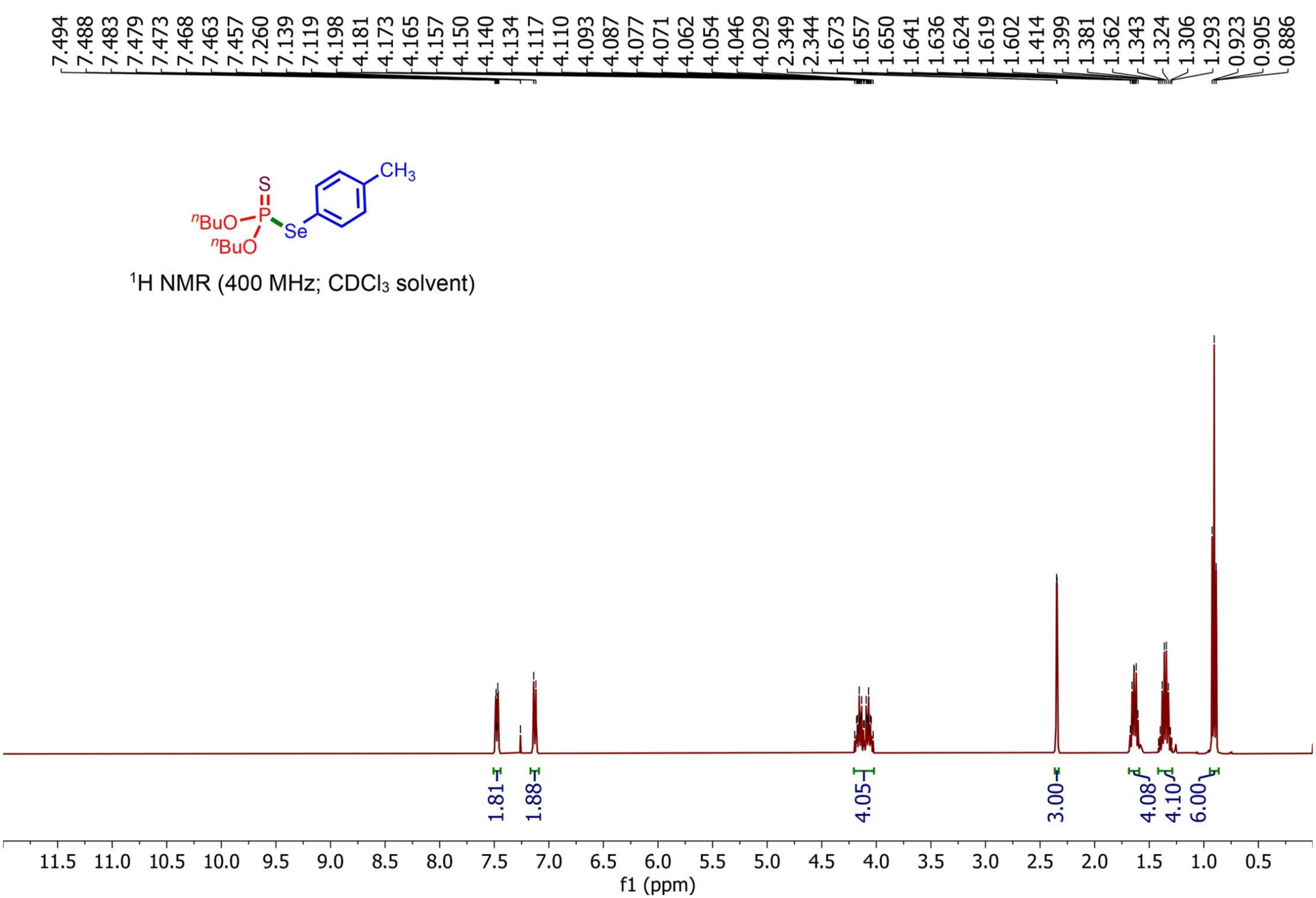

Figure S58. ^{1}H NMR spectrum of *O*,*O*-dibutyl *Se*-(*p*-tolyl) phosphoroselenothioate (**5j**)

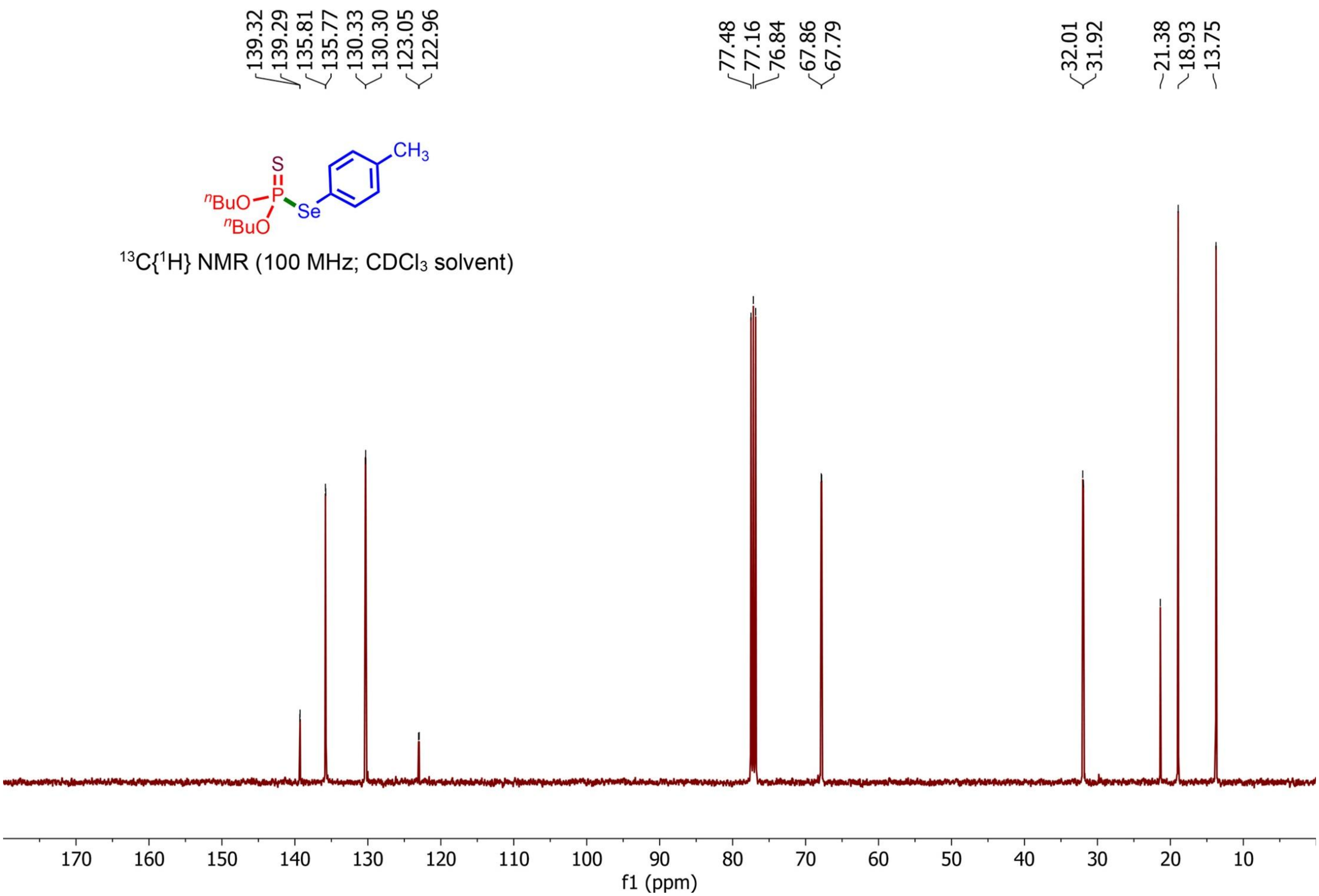

Figure S59. $^{13}C\{^{1}H\}$ NMR spectrum of *O*,*O*-dibutyl *Se*-(*p*-tolyl) phosphoroselenothioate (**5j**)

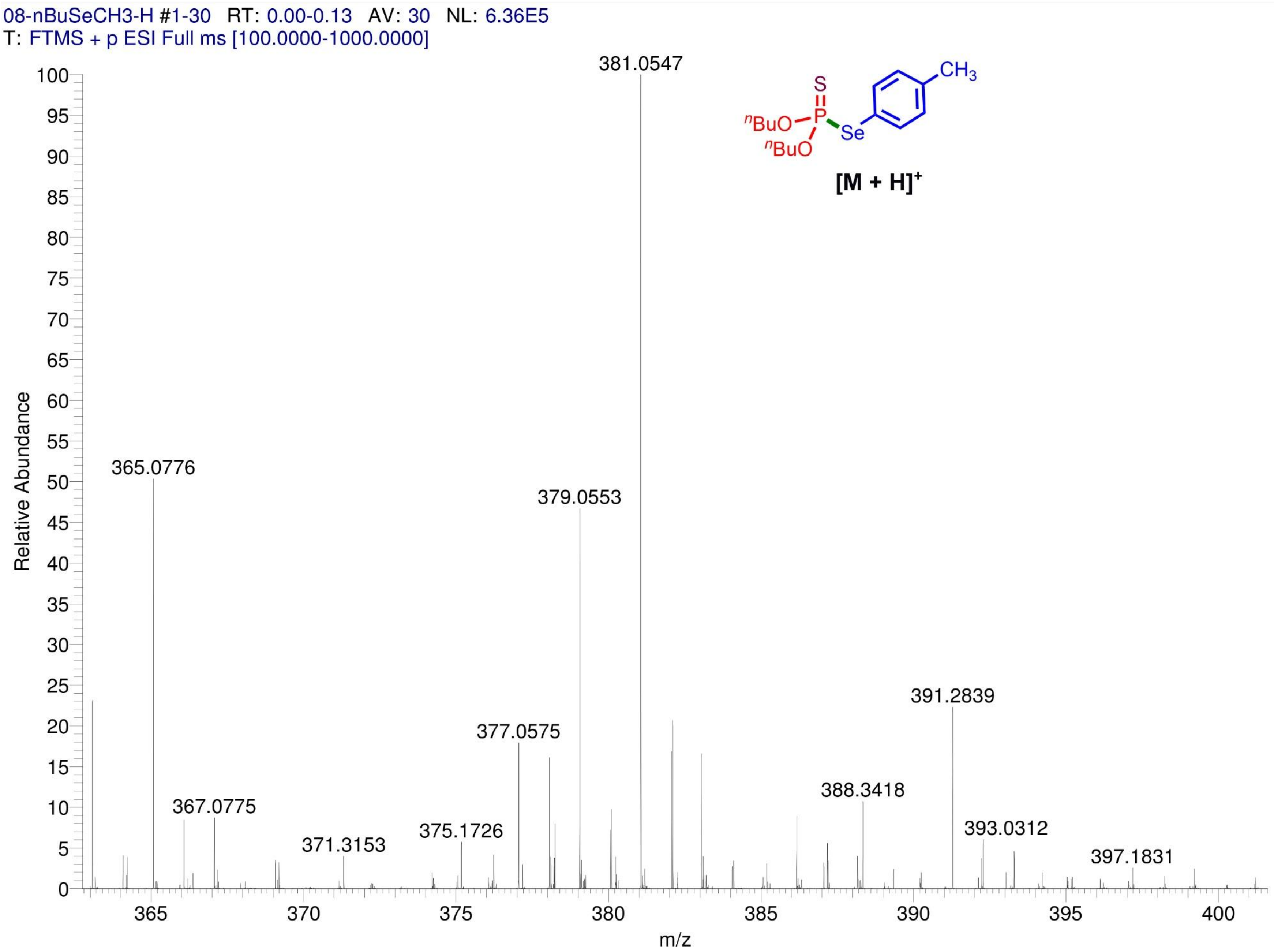

Figure S60. HRMS spectrum of *O*,*O*-dibutyl *Se*-(*p*-tolyl) phosphoroselenothioate (**5j**)

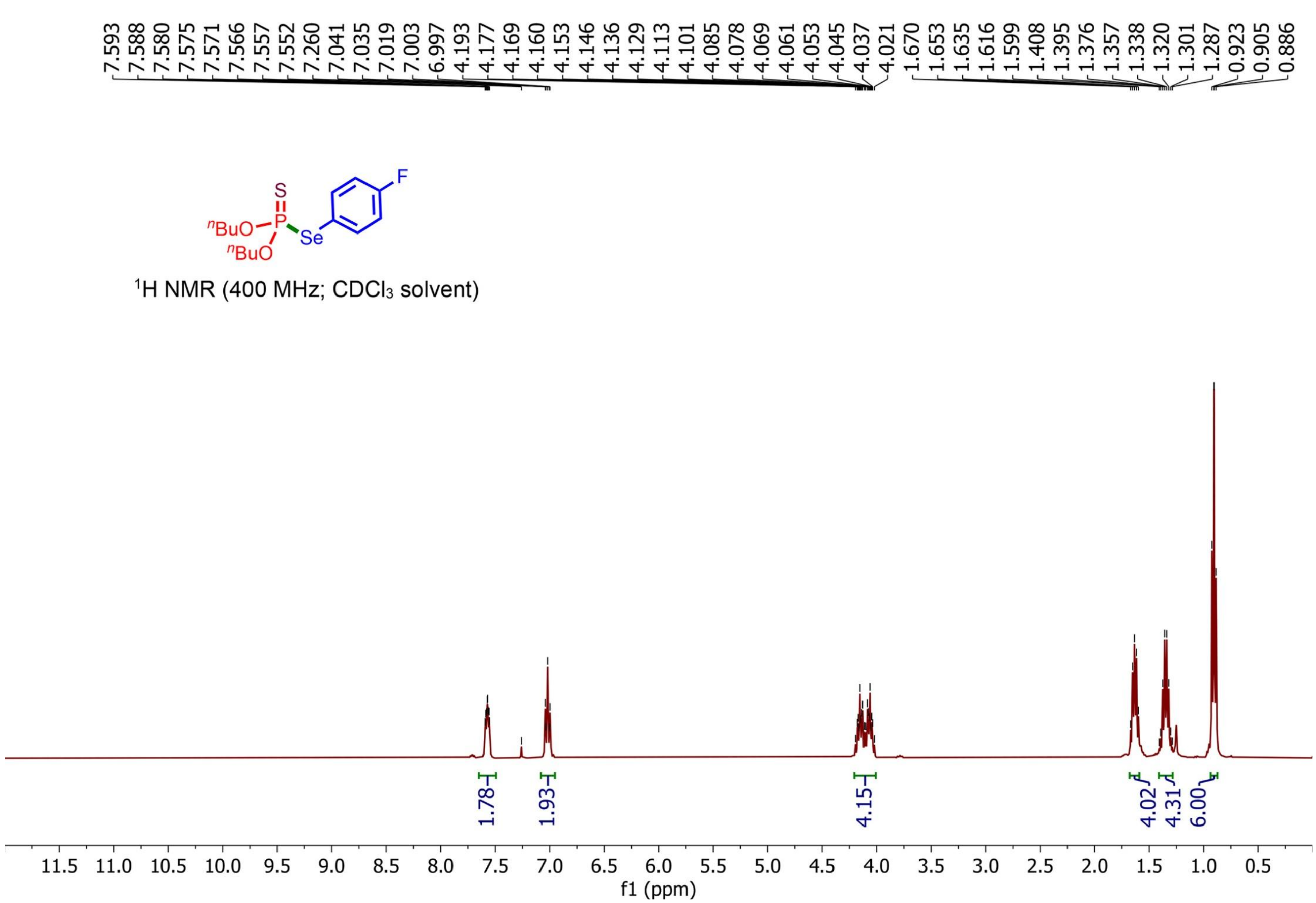

Figure S61. ^{1}H NMR spectrum of *O*,*O*-dibutyl *Se*-(4-fluorophenyl) phosphoroselenothioate (**5k**)

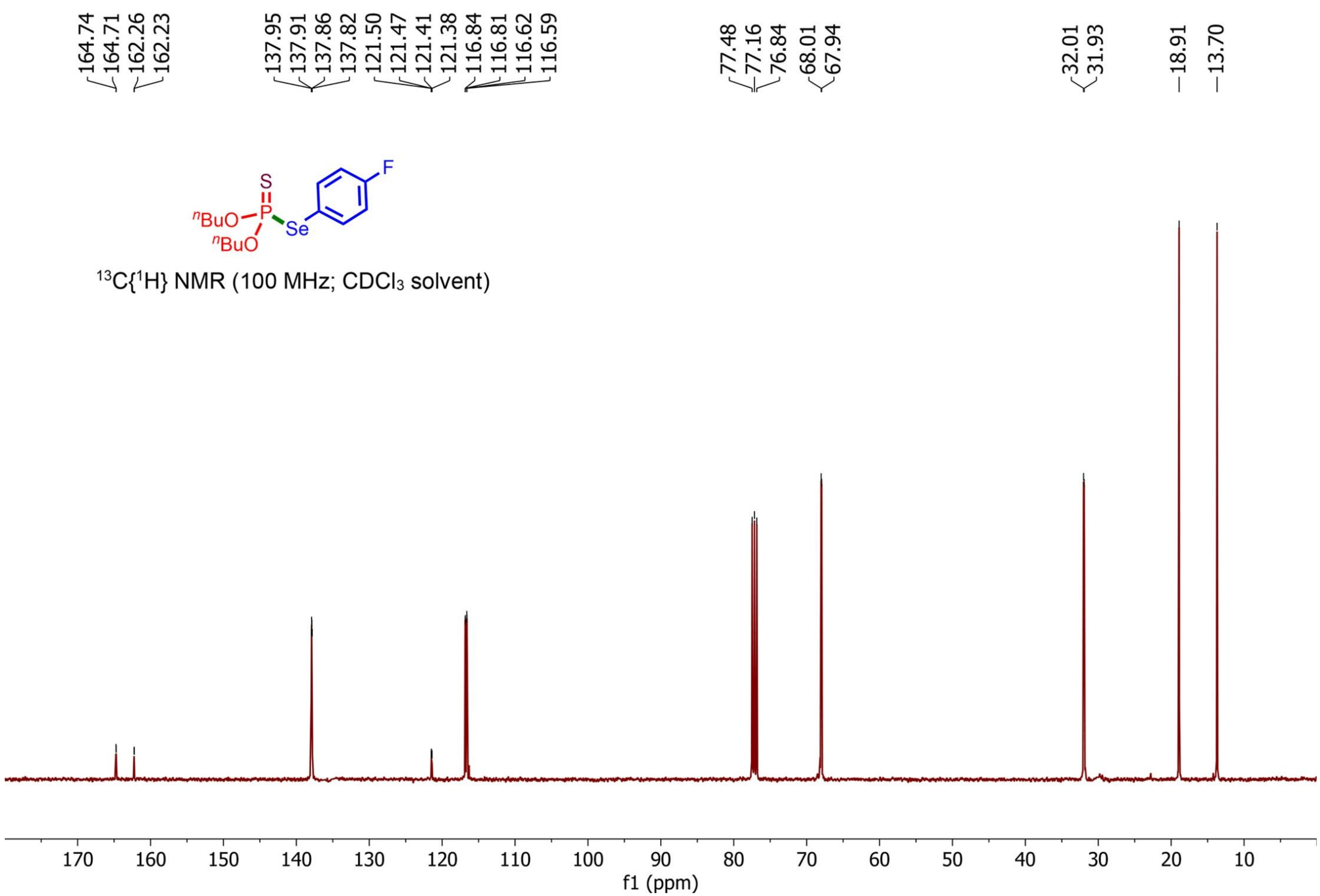

Figure S62. $^{13}C\{^{1}H\}$ NMR spectrum of *O*,*O*-dibutyl *Se*-(4-fluorophenyl) phosphoroselenothioate (**5k**)

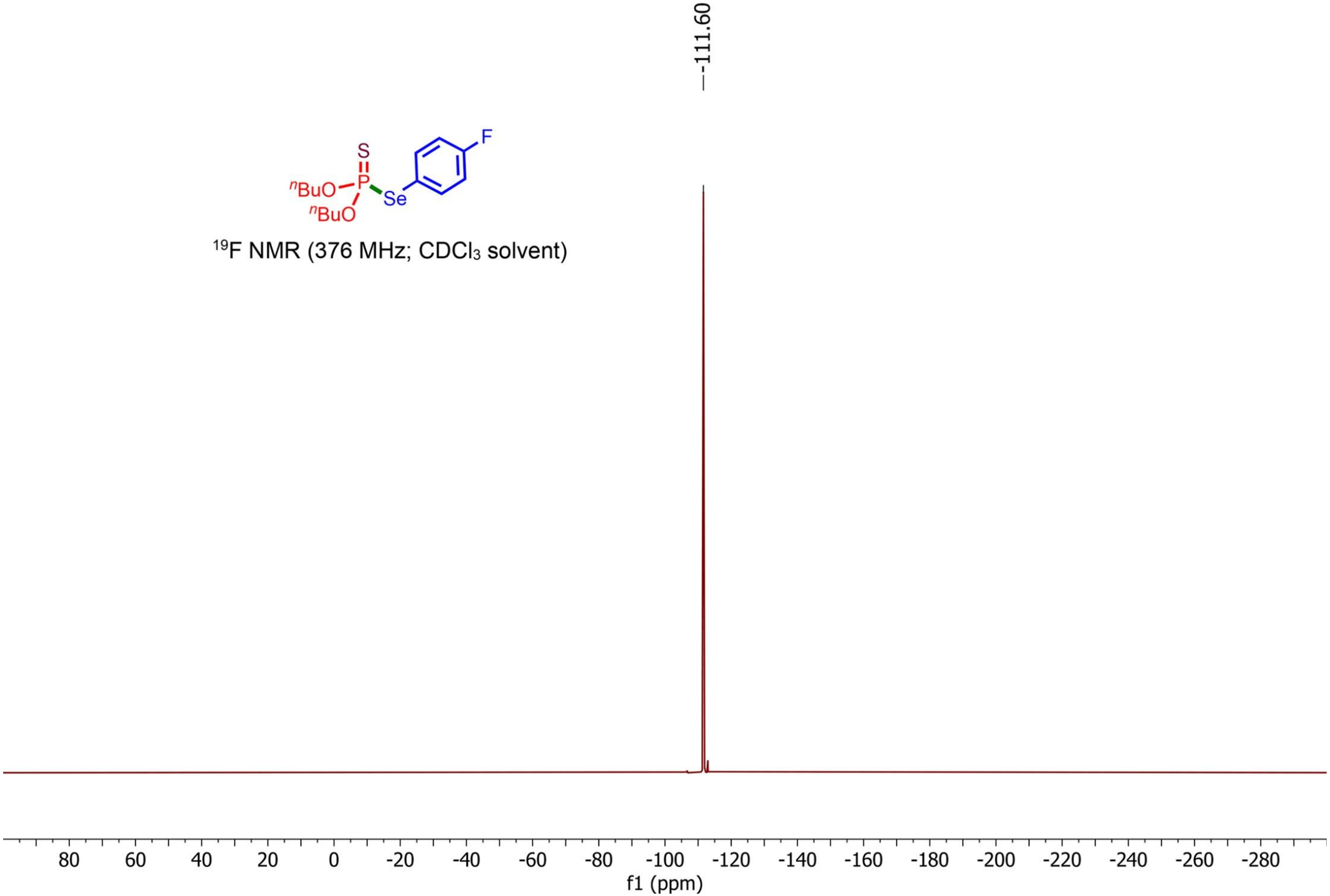

Figure S63. ^{19}F NMR spectrum of *O*,*O*-dibutyl *Se*-(4-fluorophenyl) phosphoroselenothioate (**5k**)

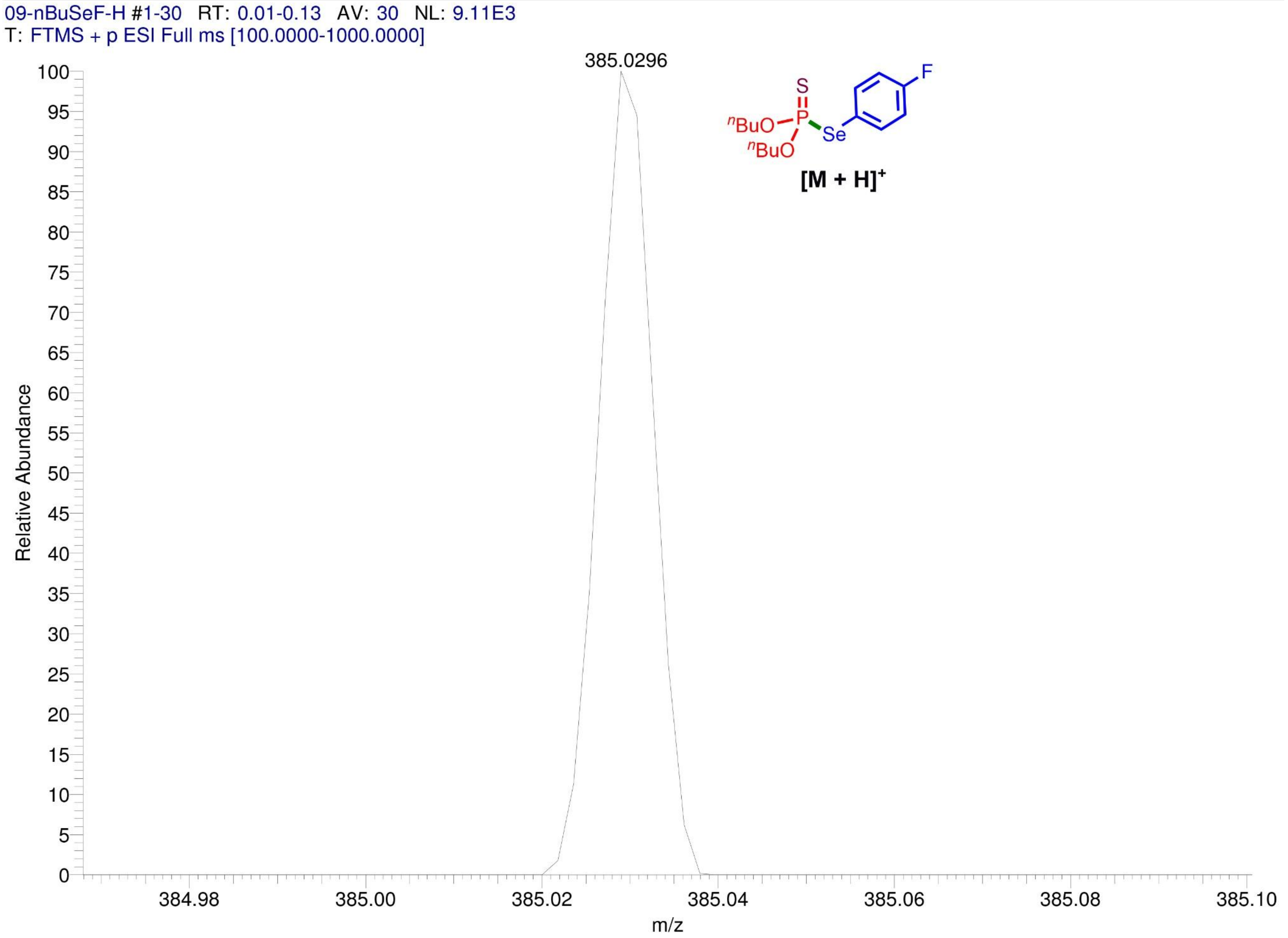

Figure S64. HRMS spectrum of *O*,*O*-dibutyl *Se*-(4-fluorophenyl) phosphoroselenothioate (**5k**)

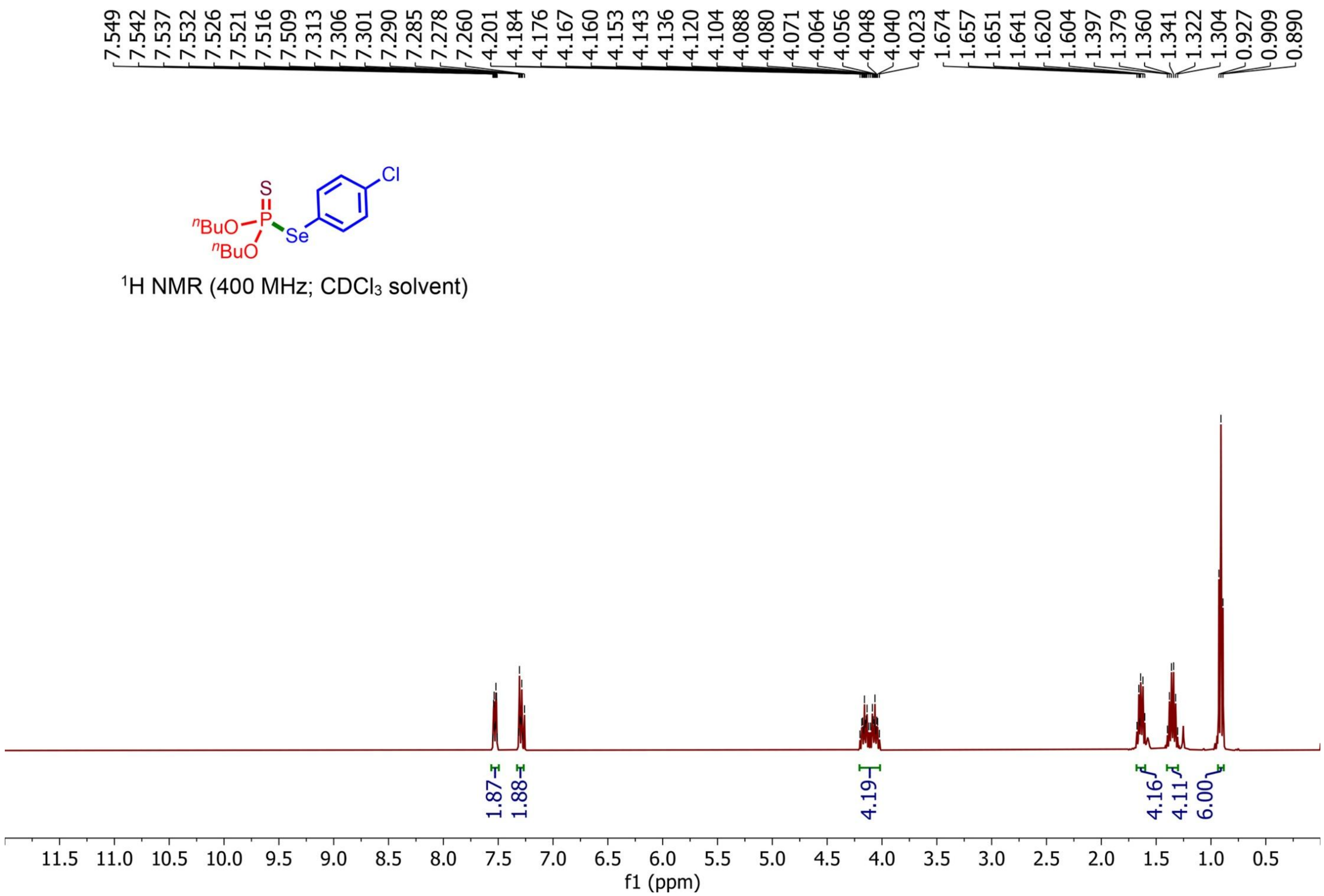

Figure S65. ^{1}H NMR spectrum of *O*,*O*-dibutyl *Se*-(4-chlorophenyl) phosphoroselenothioate (**5l**)

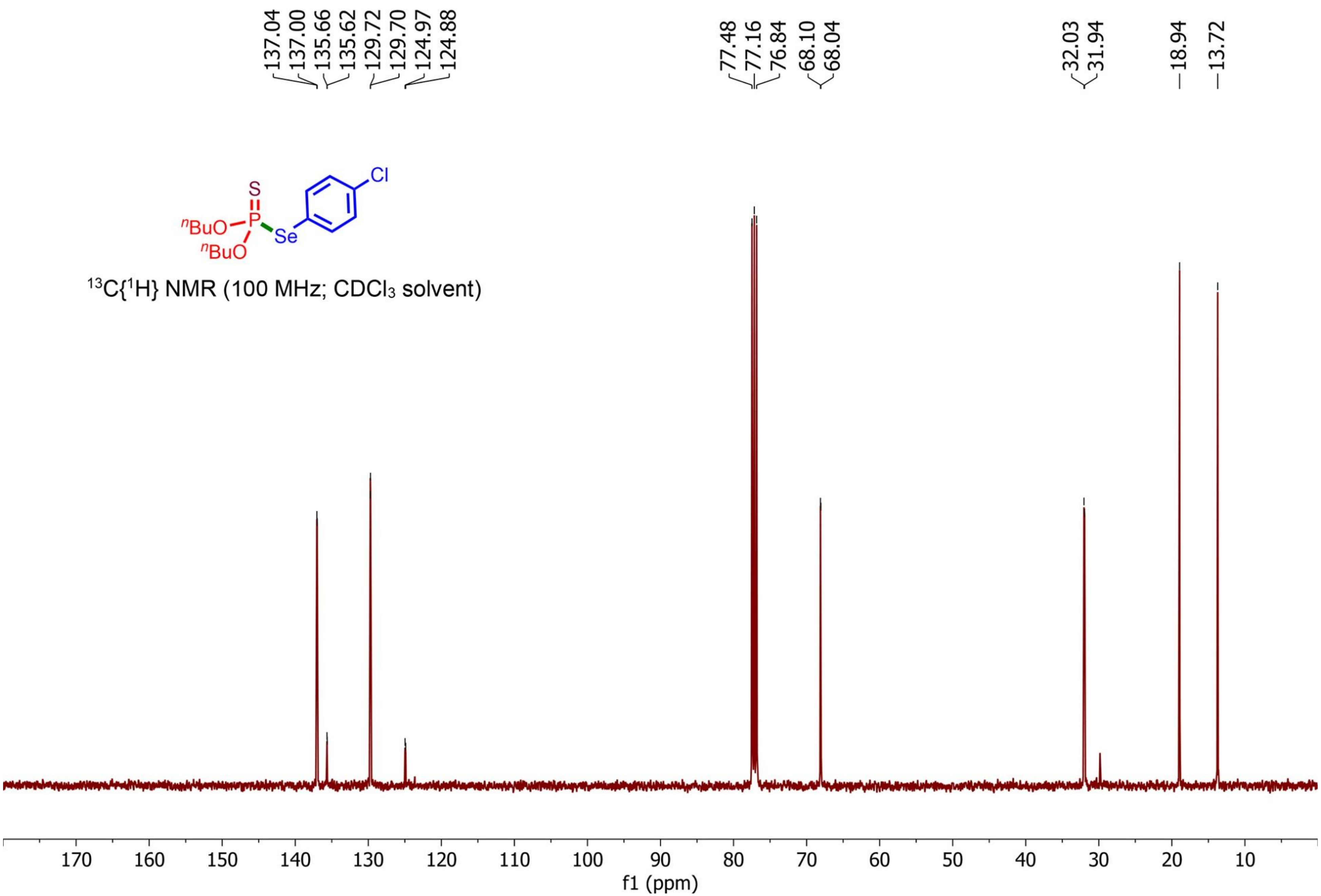

Figure S66. $^{13}C\{^1H\}$ NMR spectrum of *O*,*O*-dibutyl *Se*-(4-chlorophenyl) phosphoroselenothioate (**5l**)

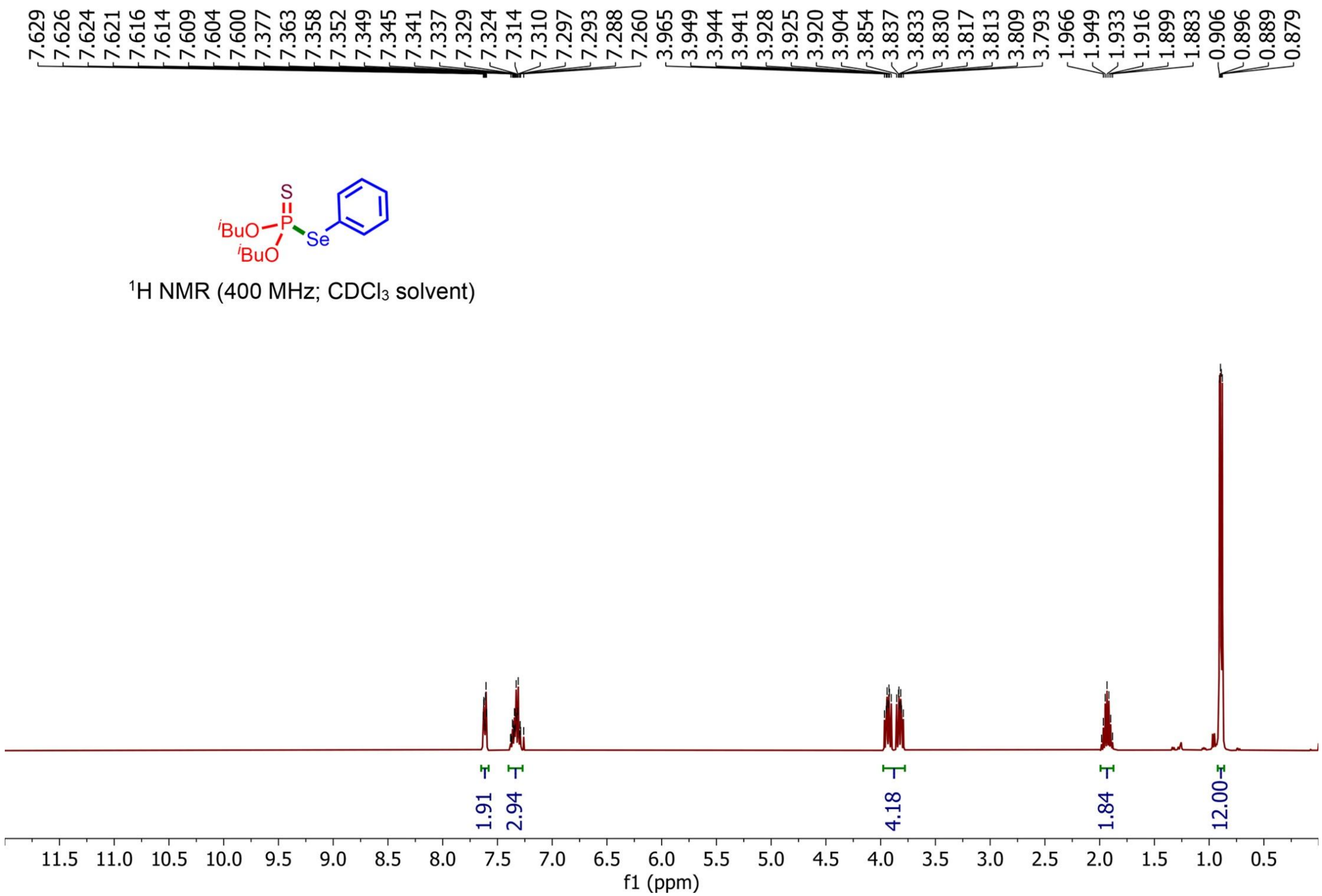

Figure S67. ^{1}H NMR spectrum of *O*,*O*-diisobutyl *Se*-phenyl phosphoroselenothioate (**5m**)

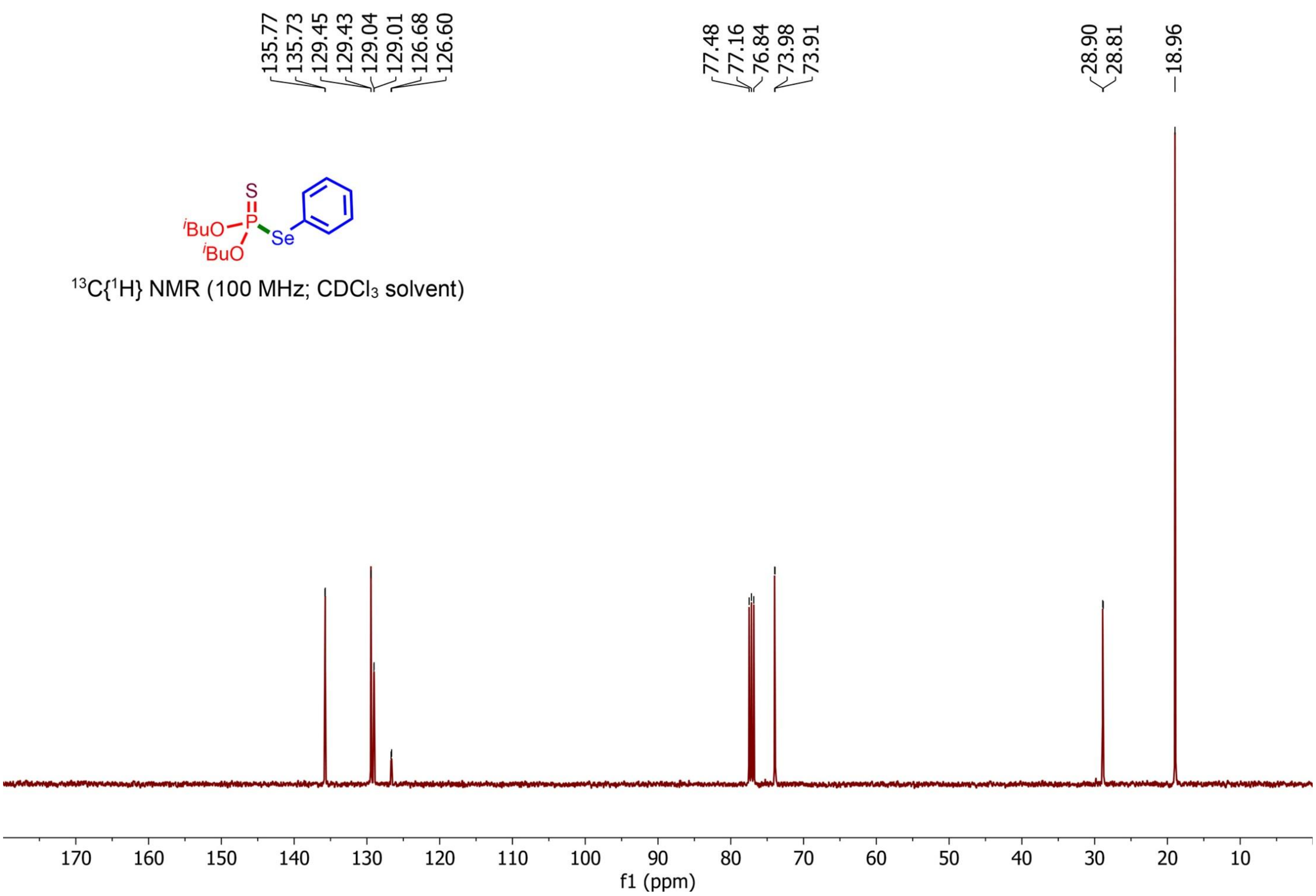

Figure S68. $^{13}C\{^1H\}$ NMR spectrum of *O*,*O*-diisobutyl *Se*-phenyl phosphoroselenothioate (**5m**)

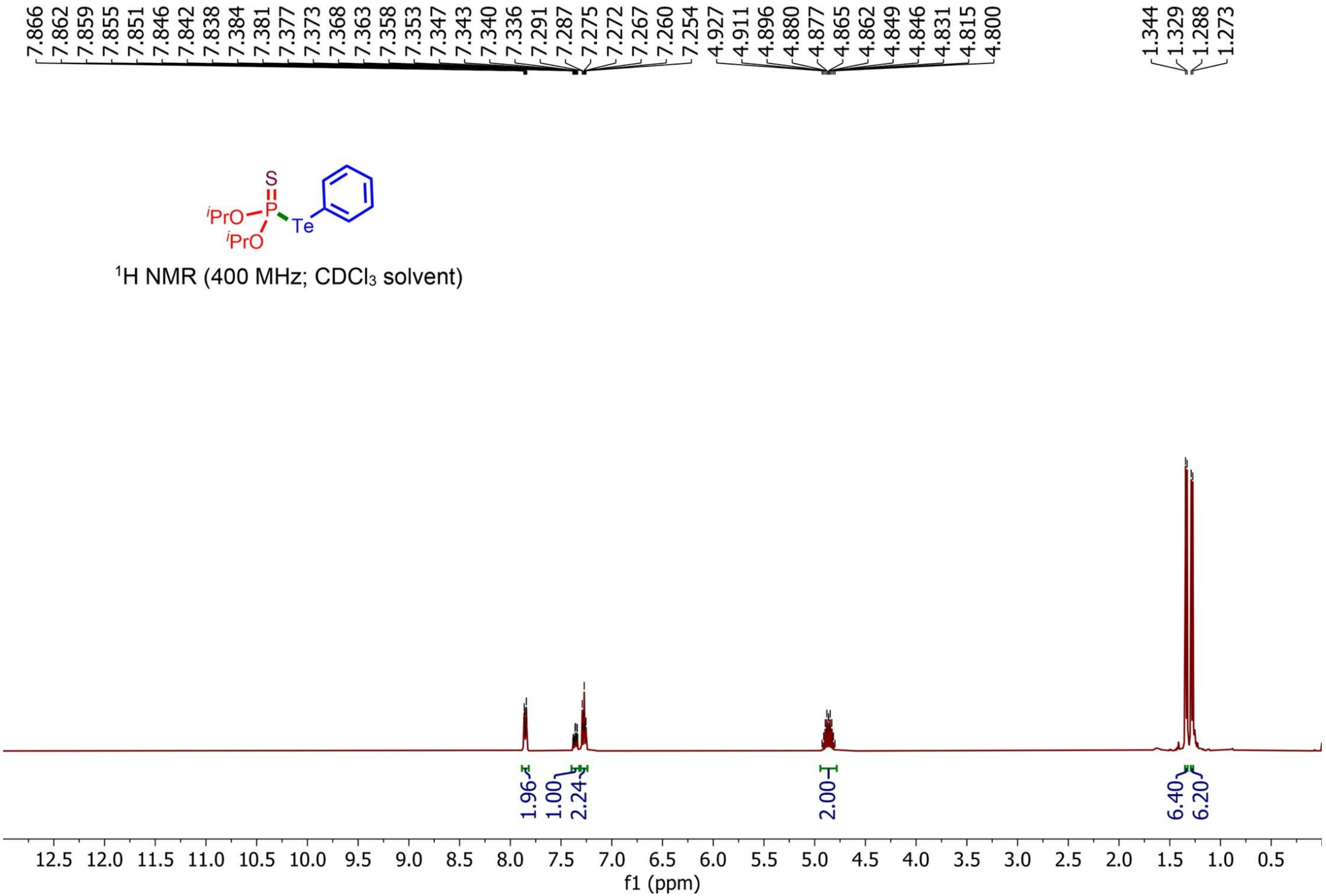

Figure S69. ^{1}H NMR spectrum of *O*,*O*-diisopropyl *Te*-phenyl phosphorotellurothioate (**7a**)

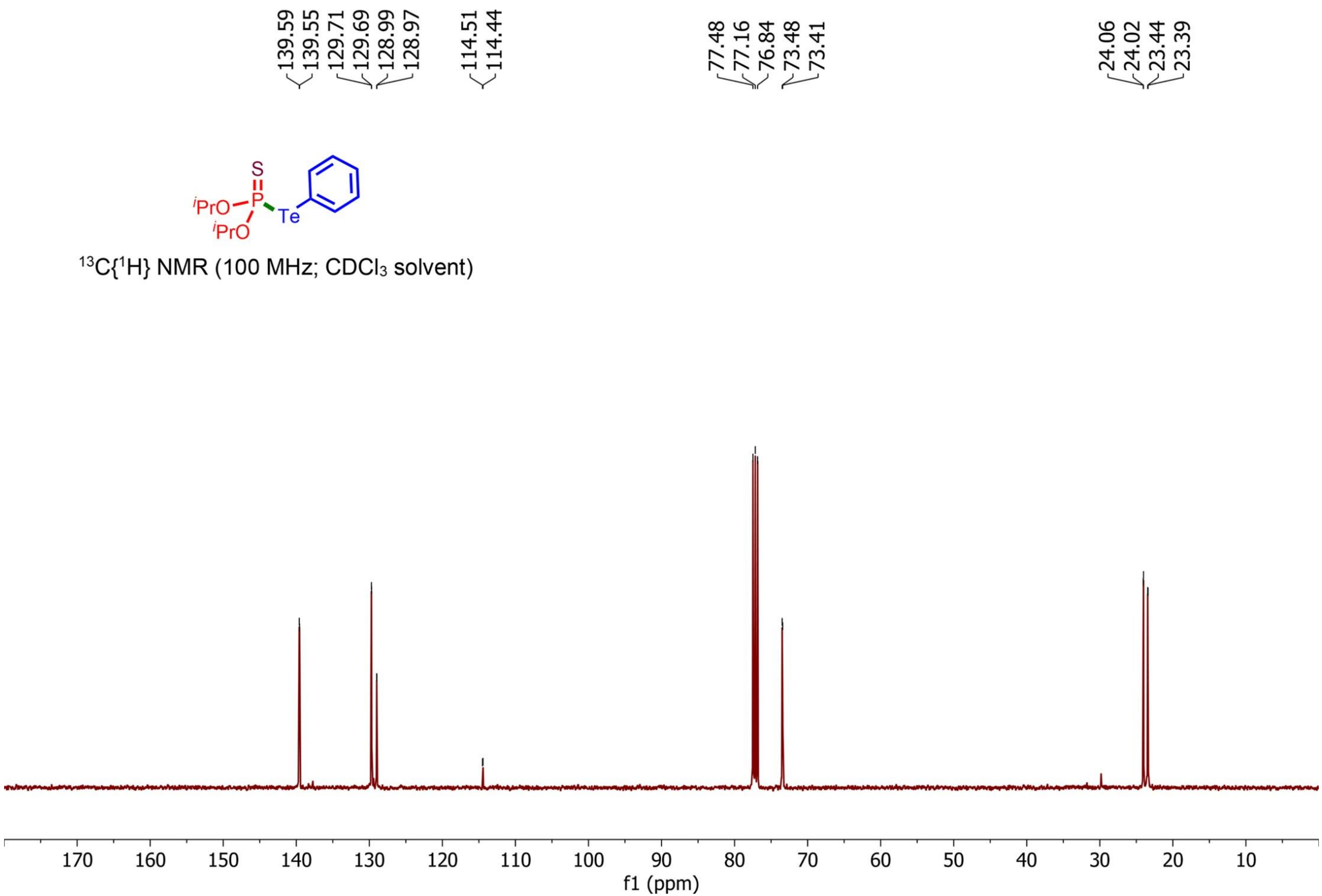

Figure S70. $^{13}C\{^{1}H\}$ NMR spectrum of *O*,*O*-diisopropyl *Te*-phenyl phosphorotellurothioate (**7a**)

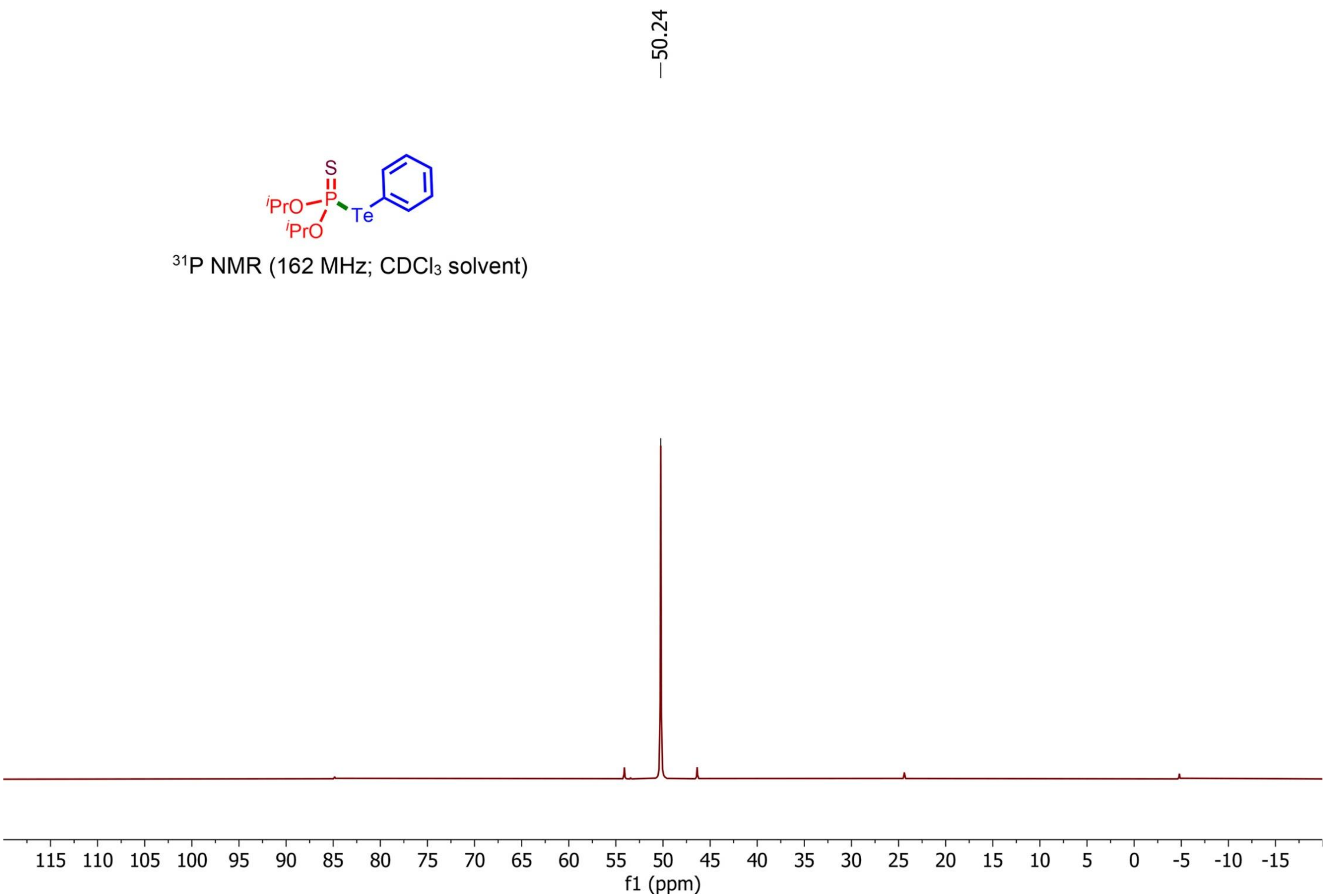

Figure S71. ^{31}P NMR spectrum of *O*,*O*-diisopropyl *Te*-phenyl phosphorotellurothioate (**7a**)

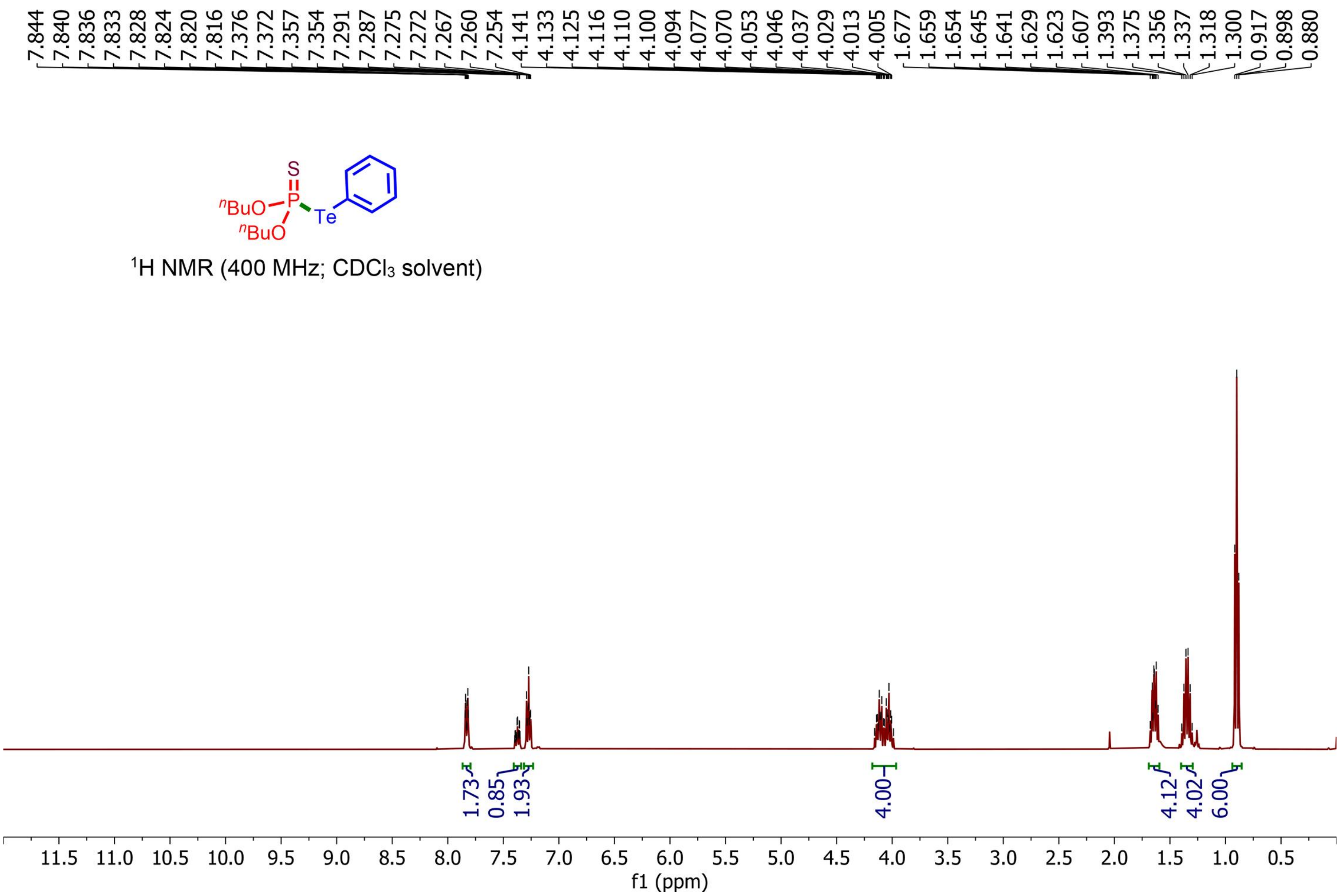

Figure S72. ^{1}H NMR spectrum of *O*,*O*-dibutyl *Te*-phenyl phosphorotellurothioate (**7b**)

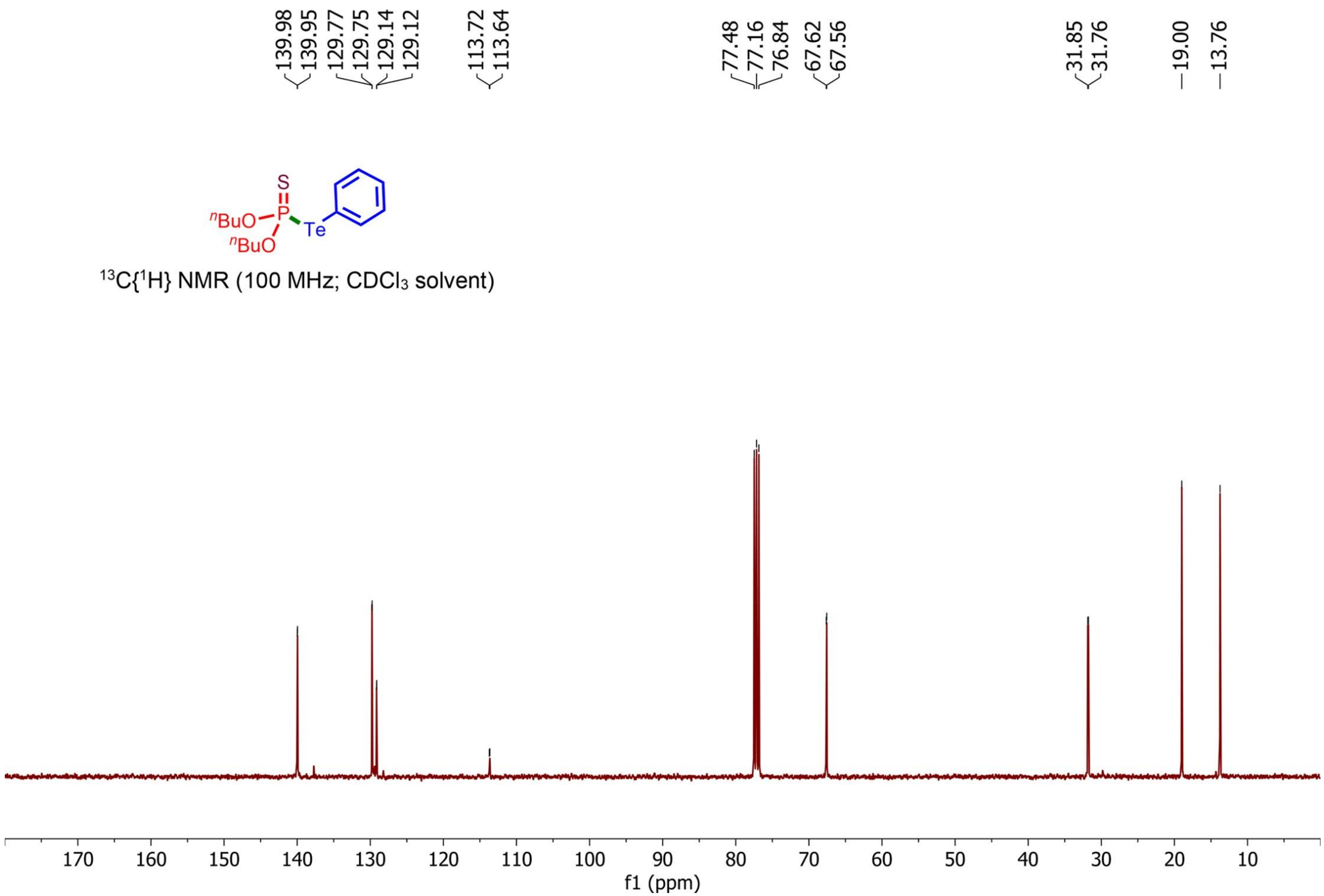

Figure S73. ^{13}C{^{1}H} NMR spectrum of *O*,*O*-dibutyl *Te*-phenyl phosphorotellurothioate (**7b**)

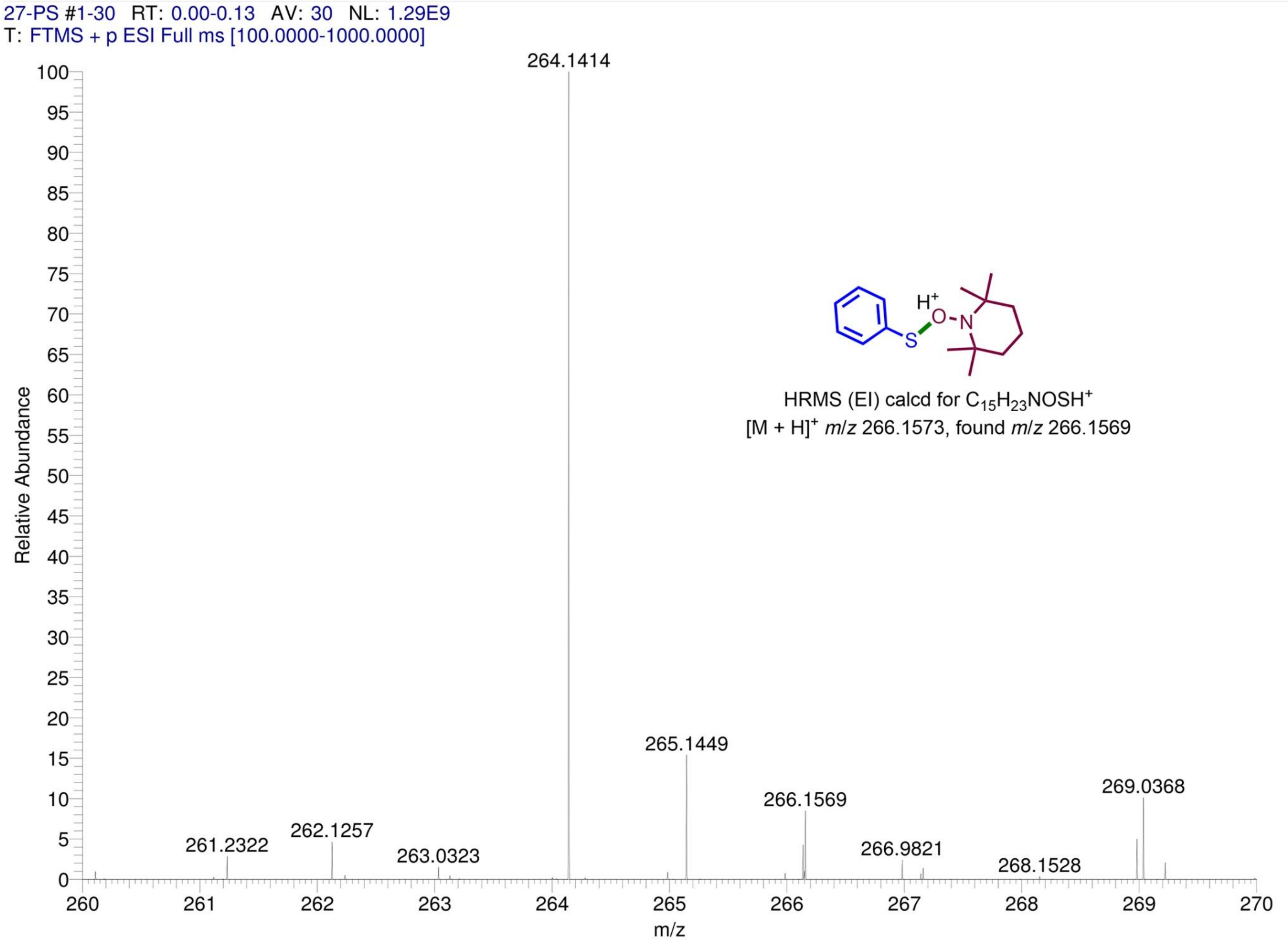

Figure S74. HRMS spectrum of adduct **9**

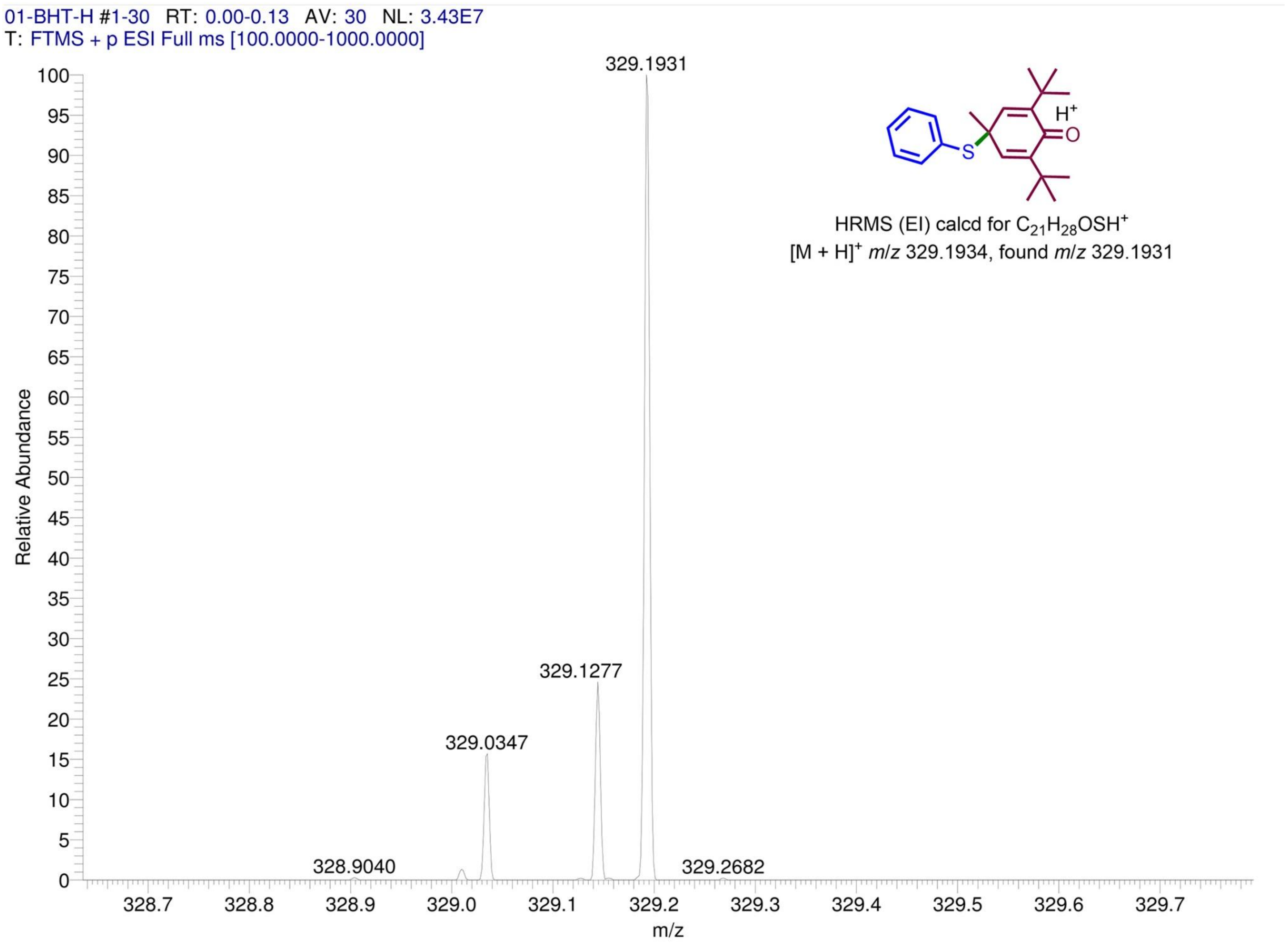

Figure S75. HRMS spectrum of adduct **10**
